# Evaluation of an interactive, case-based review session in teaching medical microbiology

**DOI:** 10.1186/1472-6920-9-56

**Published:** 2009-08-27

**Authors:** Earl L Blewett, Jennifer L Kisamore

**Affiliations:** 1 Dept of Biochemistry and Microbiology, Oklahoma State University-Center for Health Sciences, 1111 West 17th Street, Tulsa, Oklahoma, 74107, USA; 2 Department of Psychology, University of Oklahoma-Tulsa, 4502 East 41st Street-3J06, Tulsa, Oklahoma, 74135, USA

## Abstract

**Background:**

Oklahoma State University-Center for Health Sciences (OSU-CHS) has replaced its microbiology wet laboratory with a variety of tutorials including a case-based interactive session called Microbial Jeopardy!. The question remains whether the time spent by students and faculty in the interactive case-based tutorial is worthwhile? This study was designed to address this question by analyzing both student performance data and assessing students' perceptions regarding the tutorial.

**Methods:**

Both quantitative and qualitative data were used in the current study. Part One of the study involved assessing student performance using archival records of seven case-based exam questions used in the 2004, 2005, 2006, and 2007 OSU-CHS Medical Microbiology course. Two sample t-tests for proportions were used to test for significant differences related to tutorial usage. Part Two used both quantitative and qualitative means to assess student's perceptions of the Microbial Jeopardy! session. First, a retrospective survey was administered to students who were enrolled in Medical Microbiology in 2006 or 2007. Second, responses to open-ended items from the 2008 course evaluations were reviewed for comments regarding the Microbial Jeopardy! session.

**Results:**

Both student performance and student perception data support continued use of the tutorials. Quantitative and qualitative data converge to suggest that students like and learn from the interactive, case-based session.

**Conclusion:**

The case-based tutorial appears to improve student performance on case-based exam questions. Additionally, students perceived the tutorial as helpful in preparing for exam questions and reviewing the course material. The time commitment for use of the case-based tutorial appears to be justified.

## Background

Many medical schools, including Oklahoma State University – Center for Health Sciences (OSU-CHS), have discontinued the Microbiology wet laboratory in light of cost, space issues, and curricular changes [1]. Initially, to circumvent potential deficiencies in student learning as a result of wet lab removal, faculty retained the lab's time block and instead presented material typically conveyed in the laboratory session through a variety of mixed media presentations, lectures and physician-led case discussions. Student feedback was overwhelmingly negative in response to the lectures, mostly negative for the mixed media presentations and somewhat positive for the physician-led case discussions. Thus, to make more effective use of this time block, the course instructors introduced an interactive case-based tutorial in the Medical Virology module. Case-based learning has been shown to be effective for dental and allopathic medical programs in the U.S., Europe, and South America [2-5]. Problem-based learning was also considered but discarded because its use would require significantly greater faculty manpower. The interactive case-based session was designed to show students the value of basic science information in clinical applications, to provide a review of course material and to familiarize the students with differential diagnosis. Given that the majority of questions on board exams are case-based, faculty believed that such a session would also better prepare students to answer the types of questions they would encounter on board exams. The interactive case based tutorials have been used at OSU-CHS since 2006.

In 2008, OSU-CHS began a major curriculum review of its Osteopathic Physician Program. The review is on-going and includes consideration of questions such as, "How do didactic teaching, problem-based learning, case-based learning, systems approaches and other educational methods fit into the new model of health education?" OSU-CHS students already do very well on the first level Comprehensive Osteopathic Medical Licensing Examination (COMLEX) exam, which tests biomedical science knowledge. Because students typically achieve the top or near top pass rate and class average nationally, there is resistance to "fixing" how OSU-CHS faculty teach students, but there are continued calls in the medical education literature to update the curriculum [6,7]. These calls for changes correspond to upcoming changes in the certifications exams for physicians, including the United States Medical Licensing Examination™ (USMLE) and COMLEX, which will involve a reduction of biomedical science content and an increase in physical exams and clinical problems. Thus, curricular changes are necessary, but specifically how the Microbiology curriculum should be changed remains unanswered.

The current study addresses a small part of this issue. Removal of the wet laboratories from the OSU-CHS Medical Microbiology course and subsequent replacement with a mixture of lecture and tutorial periods resulted in a clear, negative response by students. Following the change, a group of students met with the faculty requesting more hands-on experience and greater student – teacher interaction. In response to this request, the course coordinator (the senior author) for the Medical Microbiology course developed an interactive, case-based session built upon a Microbial Jeopardy!™ idea from MicrobeLibrary.org [8] and a Jeopardy! PowerPoint template obtained at . Student worked in teams to demonstrate their knowledge of microbiological concepts. The session encouraged students to review and integrate basic science material in a more clinical approach. While feedback from students has generally been favourable about this component of their microbial education, the question remained "Does it do any good?" The Microbial Jeopardy!™ session is time-consuming for faculty to develop and administer and occupies a two-hour lecture block. This study was conducted to test the efficacy of the interactive session.

### Hypothesis One

Students exposed to an interactive, case-based review session would perform significantly better on case-based exam questions than students who were not exposed to the interactive, case-based session.

### Hypothesis Two

Students would respond favourably to the interactive, case-based session including indicating that the session was (a) enjoyable, (b) useful for preparing to answer case-based exam questions in the current and other classes, and (c) facilitated transfer of knowledge to realistic situations.

A two-part study was used to test these hypotheses and evaluate outcomes of this interactive, case-based review session. Part One assessed student performance; it was conducted to determine whether the interactive, case-based review session increased student performance on case-based exam questions. Part Two was conducted to examine students' perceptions of the Microbial Jeopardy! session using both a retrospective survey and course evaluations. The OSU-CHS Institutional Review Board reviewed and approved this research protocol (#2007037).

## Methods

### Participants

#### Student Performance

For Part One of the study, cohort performance data on case-based exam questions for students enrolled in the Medical Microbiology and Immunology course at OSU-CHS during spring 2004 (*n *= 98), 2005 (*n *= 98), 2006 (*n *= 97), and 2007 (*n *= 97) were obtained. The data were based on a total of 390 students across the four cohorts. More than 90% of the students have a baccalaureate degree. Across all four cohorts, students were predominantly male (55.1%, 56.1%, 56.7%, and 58.8%, respectively). For Part Two of the study, there were two separate sets of participants, those that completed the retrospective survey and those that completed course evaluations.

#### Survey of Student Perceptions

Students enrolled in the Medical Microbiology and Immunology course at OSU-CHS during spring 2006 (*n *= 97) and 2007 (*n *= 97) were recruited to complete a retrospective survey. An information sheet for consent to participate in the study was provided to all potential participants (Appendix A). Students were predominantly male in each of the cohorts (56.7%, and 58.8%, respectively). A total of 28 (28.9%) students from the spring 2006 cohort and 34 (35.1%) students from the spring 2007 cohort returned the surveys yielding an overall response rate of 32.0%.

#### Course Evaluations

Course evaluation data were collected in fall 2008 from students enrolled in the Medical Microbiology course and reviewed to assess student reactions to the Microbial Jeopardy! session offered in fall 2008. Course evaluations were only reviewed for fall 2008 because course evaluations were made compulsory at that time. In previous years, completing the evaluations was optional for students and the response rates were always less than 15%.

### Case-based Review Sessions

Case-based review sessions were available for students from 2006 onwards. Students in these cohorts self-selected into teams of eight. Prior to the session, all teams were provided with 20 to 24 cases and a series of questions about each case. The quiz show format was modelled on the game show Jeopardy!™ in which contestants try to demonstrate their knowledge of trivia across a variety of categories in order to win cash. In the game show, contestants are given "answers" and must supply the correct "question" in order to receive points. In the Microbial Jeopardy! session, student worked in teams rather than individually to demonstrate their knowledge of microbiological concepts; their responses did not have to take the form of a question; and they participated to earn course credit rather than money. Questions were grouped according to similar diagnostic cases and included categories such as: "yellow-eyed people", "nasty rashes" and "dizzy woodsmen" (see Additional File 1).

During the tutorial period, each student was expected to answer one question on behalf of his or her team. If a team answered five questions correctly, each team member received full credit for the exercise. To encourage preparation and increase interest, the first team that answered 6 questions correctly earned a 1% bonus. Students whose responses were judged to be only partially correct were allowed to solicit help from teammates or answer a follow up question from the instructor. In the first year this case-based session was offered (cohort 2006), teams competed for the chance to answer questions based on whose hand was raised first. At the request of students, to reduce competition, the procedure was changed in the second year (cohort 2007) such that a team number was pulled from a hat and a member of that team had to answer the question correctly to receive credit. In both cohorts, each student was only permitted to answer one question. The fall 2008 Microbial Jeopardy! session for which student course evaluation data were reviewed was administered in the same non-competitive format as the 2007 session.

### Measures

#### Student Performance

To assess effectiveness of the session, students' performance on case-based multiple-choice exam questions were analyzed in Part One of the study. To be included in the analysis, exam questions must have been identically worded across years, used on exams for at least two cohorts, and used with at least one cohort that was (i.e., 2006 and 2007 cohorts) and at least one cohort that was not (i.e., 2004 and 2005 cohorts) exposed to the interactive, case-based session. Exams are not returned to students and many exam questions are modified from year to year to maintain test security. Seven multiple-choice exam questions met all requirements for study inclusion.

#### Survey of Student Perceptions

In Part Two of the study, a 10-item retrospective survey that was developed for the current study (Appendix B) was used to assess student perceptions' regarding the Microbial Jeopardy! session. Items were combined to form 3 scales: satisfaction, utility of the session, and facilitation of knowledge transfer. The *satisfaction *scale was composed of 3 items (items 1, 2, and 3) which assessed the extent to which participants perceived the session to be engaging and enjoyed the session. The *utility *scale was composed of 3 items (items 4, 5, and 7) designed to assess the extent to which participants perceived the session was a useful way to prepare for case-based questions on the Medical Microbiology and Immunology exam as well as case-based exam questions in other courses. Finally, the *facilitation of knowledge transfer *scale was a 2 item scale (items 6 and 8) designed to assess the extent to which the cases used in the session enhanced application of basic science information in clinical settings. Reponses to scale items were made on a 4-point Likert-type scale ranging from *strongly disagree *(1) to *strongly agree *(4). No neutral response category was included although participants were able to choose "not applicable" as a possible response option. Cronbach's alpha [9], a measure of internal consistency reliability, was computed for each scale based on the total sample of respondents. Internal consistency reliability coefficients for the three scales were acceptable and are provided in parentheses along the diagonal in Table 1. Two additional questions were included in which participants indicated their grade for the course and the percentage of class sessions which they attended. Means, standard deviations, and correlation values are reported in Table 1. Means and standard deviations separated by cohort, also provided at the bottom of the table.

**Table 1:** Descriptive Statistics and Correlations among Variables

Variable				1	2	3	4	5
1.	Satisfaction	*M *=	2.43	(.95)				
		*SD *=	0.87	*N *= 62				
2.	Utility	*M *=	2.49	.77**	(.88)			
		*SD *=	0.80	*N *= 58	*N *= 58			
3.	Knowledge	*M *=	3.02	.50**	.64**	(.69)		
	Transfer	*SD *=	0.65	*N *= 56	*N *= 55	*N *= 56		
4.	Course	*M *=	1.94	.13	.12	.14	--	
	Grade	*SD *=	0.90	*N *= 62	*N *= .39	*N *= 56		
5.	Attendance	*M *= *SD *=	4.18 1.09	.32* N = 61	.27* N = 58	.34** N = 56	-.09 *N *= 61	--

2006 cohort only			*M *=	2.33	2.27	2.83	1.85	4.15
			*SD *=	0.88	0.81	0.72	0.93	1.26

2007 cohort only			*M *=	2.55	2.75	3.23	2.04	4.22
			*SD *=	0.86	0.72	0.49	0.88	0.85

*p < .05; ** *p *< .01

Note: Internal consistency reliability coefficients (alpha) are reported on the diagonal.

Course grades coded as: A = 1; B = 2; C = 3; U = 4

Attendance coded as: 0%–20% = 1; 21%–40% = 2; 41%–60% = 3; 61%–80% = 4; 81%–100% = 5

#### Course Evaluations

Part Two of the study also included assessment of student responses on the 2008 Medical Microbiology course evaluations. Quantitative responses to the question, "Laboratory sessions were useful" were assessed and students' written responses were also reviewed.

### Procedure

#### Student Performance

In Part One of the study, exam summary statistics and exam items were obtained from the course coordinator for Medical Microbiology and Immunology. Identical exam questions were matched across multiple study cohorts. A total of seven, identical case-based exam questions were identified. The proportion of students in each cohort who answered each of the seven questions correctly was recorded (Table 2). Multiple, two-sample t-tests of proportions were conducted consistent with procedures outlined by Lind, Marchal and Wathen [10]. Significant differences based on exposure to the interactive session were expected but no significant differences based on cohort group alone were expected.

**Table 2:** Proportion of correct responses by cohort

	2004	2005	2006	2007	**Results of two sample z tests of proportions**				
					**Tests of Differences based on Session**			**Tests of Cohort Differences**	
					
					Comparison	z	Direction	Comparison	z
					
1	0.51	--	--	0.69	2004 vs 2007	-2.52*	predicted	--	--
2	--	0.47	--	0.78	2005 vs 2007	-4.53**	predicted	--	--
3	0.50	--	--	0.56	2004 vs 2007	-0.79	predicted	--	--
4	0.70	--	0.85	--	2004 vs 2006	-2.36*	predicted	--	--
5	0.54	--	0.63	0.59	2004 vs 2006 2004 vs 2007	-1.25 -0.66	predicted predicted	2006 vs 2007	0.59, ns
6	0.64	0.88	--	0.71	2004 vs 2007 2005 vs 2007	-1.02 +2.87**	predicted opposite	2004 vs 2005	-3.85**
7	0.97	0.99	--	0.97	2004 vs 2007 2005 vs 2007	0.00 +1.02	equivalent opposite	2004 vs 2005	1.01, ns

z = +/-1.96 used as criterion for differences at 95% confidence (denoted by *)

z = +/-2.56 used as criterion for differences at 99% confidence (denoted by **)

Note: 2004 *n *= 98, 2005 *n *= 98, 2006 *n *= 98, 2007 *n *= 97

#### Survey of Student Perceptions

For Part Two of the study, the retrospective survey was distributed to participants via their campus email. Participants had 10 days to complete the survey and were sent a reminder email two days prior to the deadline. All completed surveys were turned into the Office of Educational Development rather than to the researcher in order to maintain participant anonymity.

#### Course Evaluations

Course evaluation data that were available to the senior author who was course coordinator for the Medical Microbiology course in 2008 were reviewed. Students completed the compulsory course evaluations prior to final exams.

## Results

### Student Performance

In Part One of the study, a total of 10 comparisons were made across the 7 questions. This was possible because three questions (questions 5, 6, and 7) were used in three separate years. Using a significance criterion of α = 05 results revealed that there were significant differences in performance as predicted based on the interactive session on three (1, 2 and 4) comparisons (Table 2). Additionally, trends in performance for an additional four comparisons (3, 5a, 5b, and 6a) were in the predicted direction. Cohort performance for another question (7) was equivalent in one comparison (7a) but slightly opposite of the direction predicted in another (7b). These results are most likely due to ceiling effects for that question in that most students answered the question correctly. A significant, contrary finding was noted in only one comparison (6b). This result could be due to the cohort difference noted below. Overall, Hypothesis One was supported.

Given that this was not a randomized study, we also tested for possible differences among pairs of cohorts when both cohorts were or were not exposed to the Microbial Jeopardy! session. Multiple, two-sample t-tests of proportions were conducted. The purpose of these tests were to ensure that cohorts were essentially equivalent, that is we tested to ensure that pre-existing differences across cohorts was not an issue that would confound results. Out of the three cohort tests run, only question 6 produced a significant result (2005 vs. 2007 comparison). This suggests that although some pre-existing differences existed between the cohorts, in general, cohorts that were treated the same performed essentially equivalently on the exam. The unexpected difference noted for comparison 6b could be the result of additional coverage of a specific concept for students in cohort 2005 such as increased discussion of the concept during class in response to a student question.

### Survey of Student Perceptions

In Part Two of the study, a total of 62 completed surveys were returned yielding an effective response rate of 32%. As described above, survey items were grouped into 3 scales: satisfaction, utility of the session, and facilitation of knowledge transfer. In general, students responded quite favourably regarding the Microbial Jeopardy!'s ability to facilitate the transfer of knowledge to clinical settings (Table 1). Students were less enthusiastic regarding their satisfaction with the session or the utility of the session for preparing them for the course exam. Due to differences in the administration of the session for members of the two cohorts, further analysis was conducted to test each cohort separately (bottom of Table 1). While students in both the 2006 (*M *= 2.83) and 2007 (*M *= 3.23) cohort assessed the session as beneficial for facilitating knowledge transfer, as indicated with a mean greater than 2.5, the students in the two cohorts differed regarding their assessments of satisfaction and utility. The 2006 cohort rated the session below neutral on these factors while the 2007 cohort rated the session above neutral on these factors. The differences may be due to removing the competitive aspects from the second session. After the session was used in 2006, students indicated that they did not like for teams to "compete against one another" thus the format was changed to a non-competitive drawing of a team name to determine who was eligible to answer the next question.

The demographic data indicated that 51.6% of the respondents attended 81% to 100% of class sessions while an additional 24.2% indicated attending between 61 and 80% of sessions. Exactly half of respondents indicated earning a grade of B in the course and 32.3% indicated earning a grade of A. No significant differences in self-reported grade or class attendance were noted for students in the 2006 and 2007 cohorts.

#### Course Evaluations

For the 2008 course evaluation data, a total of 93 students (93%) responded to the question "Laboratory sessions were useful." Of these respondents, 32 (34.4%) students disagreed with this statement while 30 (32.3%) strongly disagreed. Three respondents (3.2%) agreed and the remainder were neutral (30.1%) for a mean of 2.18. While this indicates that students generally did not find the laboratory sessions to be useful, written comments provide a different interpretation of the findings given that lab sessions included a variety of teaching modalities, only one of which was the Microbial Jeopardy! session. There were 29 written responses from the 93 students (31.2%). Of the written responses, 19 (65.5%) included negative comments about the laboratory sessions, mostly strongly negative. There were only 7 completely positive comments, six of which included comments about the case-based tutorial sessions and four of which included comments about the clinical interaction (13.8%). Some responses included both positive and negative comments. The case-based tutorials received no negative comments. Even 5 of the 19 individuals who provided overall negative comments about the laboratory sessions did include favourable comments about the case-based tutorial (26.3%). While responses indicated that students would like to move away from mixed media presentations and lectures in the laboratory session, their comments appear to support continuance of the case-based interactions.

## Discussion

### Summary

As OSU-CHS goes through curriculum review, the Microbiology faculty are trying to determine how the course can be re-structured following discontinuation of a wet laboratory. The current lectures, mixed media presentations and tutorials do not seem to be an effective use of the time. There has been positive feedback to clinician-led case discussions but this requires recruiting and scheduling many physicians and also is a financial burden on the department. There has also been positive feedback with the quiz show format and cased-based approach. Introducing more case-based tutorials would not require nearly as much faculty time and could be performed by departmental faculty at no incremental cost. This preliminary data suggest there would be benefit to continuing and introducing more interactive case-based tutorials in the OSU-CHS Medical Microbiology course. The Microbiology faculty plan to increase the number of interactive, case-based sessions used in the future to see if new cohorts also demonstrate improved performance compared to the 2004 and 2005 cohorts. Part Two of the study shows that student feedback further supports continued use of the case-based tutorials. Student responses on the most recent course evaluations reflect this as well.

### Study Limitations

There are a number of limitations of the current study. First, the response rate (32%) for the retrospective survey was low. This response rate, however, is consistent with typical response rates for course feedback surveys that that do not include personal contact at the time of survey dissemination. For example, Dommeyer, Baum, Hanna and Chapman [11] found a significant difference in response rates across eight course pairs; response rates for course evaluations administered online were significantly lower (*M *= 29%) than for course evaluations administered in class (*M *= 70%). Despite the differences in response rates for in their course evaluation study, however, Dommeyer et al. did not find differences in mean course evaluation ratings. For the current study, besides lack of personal contact during survey dissemination, the low response rate for the current study could be the result of the relatively short turnaround time provided for survey completion and lack of availability of students at the time the survey was administered as this was the time period when they were changing their clinical rotations. There is no way, however, to know whether the respondents who did complete the survey are truly representative of all the students in the cohorts exposed to the Microbial Jeopardy! sessions thus, conclusions drawn from the retrospective survey should be considered with caution until more research can be conducted that corroborates or refutes the current findings.

A second limitation of the current study is that only two cohorts of students who were exposed to the session were examined and they participated in the sessions somewhat differently. The second cohort had the competitive aspects reduced in the activity. Inclusion of additional cohorts of students who participated in the tutorial would be beneficial; however, OSU-CHS has incorporated use of the TurningPoint audience response system into the Medical Microbiology course which would have introduced a significant confounding factor on exam performance for students in the 2008 cohort. Also, exam questions for the 2008 and 2009 cohorts were changed and thus are not directly comparable to questions used for the 2004 through 2007 cohorts.

Third, assessments of knowledge in Part One of the study was based on only seven items total, with only a subset of the cohorts tested with each question. While the pattern of results is generally consistent across the items, the total number of items available for analysis was limited, creating concerns for content coverage. Further research is needed that includes evaluation of the performance of multiple cohorts that are and are not exposed to the Microbial Jeopardy! session using a greater number of comparable items.

### Directions for the Future

Results of the current study are consistent with the positive results demonstrated by other studies that examined case-based medical teaching. For example, interactive case-based teaching improved learning outcomes and increased student satisfaction in a Dermatology program [12]. The authors were able to show significant differences between students taking the case-based approach versus the standard lecture course. Results of that work may be clearer than those of the current study because the Dermatology study had more participants (*N *> 200), incorporated bedside teaching and used more sessions. In another study, an electronic, interactive, case-based cytopathology component was found to be useful in second year medical student teaching [13].

Future work is needed that further examines the potential and limitations for using the interactive, case-based Microbial Jeopardy! session as an alternative to wet lab instruction. It is possible that this session may serve as an impetus for inclusion of additional case-based exercises presented in different formats in the future. For example, in spring 2009, Bacterial Jeopardy! was added to the Medical Microbiology course.

Furthermore, it is conceivable that case-based questions and examples could be incorporated into a computer program such as the dermatology and venereology program described by Wahlgren, Edelbring, Fors, Hindbeck and Ståhl [14]. Such a program would be helpful in illustrating the linkage between basic science knowledge and clinical application of microbiological concepts. Finally, it has been suggested that the Medical Microbiology faculty incorporate problem-based learning as the interactive tutorial. This module could have been developed as a problem-based learning exercise and can easily be modified to this format. The case-based approach, however, only requires one faculty facilitator, can be applied to a large group, and is less susceptible to intra-group problems [15].

## Conclusion

Results of the current study appear to support continued use of the interactive case-based session. While prospective research is needed to best assess the utility of the session, the current study does provide support for session benefits.

## Competing interests

The authors declare that they have no competing interests.

## Authors' contributions

ELB designed the study, developed the Microbial Jeopardy! exercises, collected the archival data, and designed and disseminated the survey. JLK participated in the design of the study and survey, performed all statistical analyses, and wrote the initial draft of the paper. The authors worked collaboratively on manuscript revisions. Both authors read and approved the final manuscript.

## Authors' informations

ELB is an Associate Professor of Microbiology at Oklahoma State University and is course coordinator for the Medical Microbiology and Immunology course. He is a molecular virologist who works with herpes viruses of primates. His primary research interests are developing and assessing diagnostic assays for viral disease.

JLK is an Associate Professor of Psychology at the University of Oklahoma-Tulsa. She is a proponent of using active and experiential learning in the courses that she teaches. Her primary research interests deal with measurement, research methodology, and statistical techniques.

## Appendices

### Appendix A – Information Sheet for Consent to Participate in a Research Study

My name is Dr. Earl Blewett, and I am the Course Coordinator for the Medical Microbiology and Immunology course at OSU CHS-COM. I am requesting that you volunteer to participate in a research study titled Educational Efficacy of a Case-Based Interactive Instructional Session. You were selected as a possible participant because you were enrolled in the Medical Microbiology and Immunology course during Spring 2006 or 2007. Please read this information sheet and contact me to ask any questions that you may have before agreeing to take part in this study.

#### Purpose of the Research Study

The purpose of this study is to determine whether interactive, case-based sessions are an effective teaching tool for the Medical Microbiology and Immunology course.

#### Procedures

If you agree to be in this study, you will be asked to complete the attached survey and return it to the Office of Educational Development.

#### Risks and Benefits of Being in the Study

There are no intended risks of participating in the study. Benefits to participation include improving course-related teaching methods.

#### Compensation

You will not be compensated for your time and participation in this study.

#### Voluntary Nature of the Study

Participation in this study is voluntary. Your decision whether or not to participate will not result in penalty or loss of benefits to which you are otherwise entitled. If you decide to participate, you are free not to answer any question or discontinue participation at any time without penalty or loss of benefits to which you are otherwise entitled.

#### Length of Participation

Completion of the attached survey is expected to take less than 5 minutes.

#### Confidentiality

Do not put your name on the survey. The records of this study will be kept private and your supervisor will not have access to your responses. In published reports, there will be no information included that will make it possible to identify you as a research participant. Research data will be stored securely on a password protected computer. Paper copies of the survey will be secured in a locked cabinet. Only approved researchers which include myself and a data analyst will have access to the records.

#### Contacts and Questions

If you have concerns or complaints about the research, the researcher(s) conducting this study can be contacted at Dr. Earl Blewett, 561–8405, earl.blewett@okstate.edu or Jennifer Kisamore (data analyst) 660–3603. In the event of a research-related injury, contact the researcher(s). You are encouraged to contact the researcher(s) if you have any questions. If you have any questions, concerns, or complaints about the research and wish to talk to someone other than the individuals on the research team, or if you cannot reach the research team, you may contact the Oklahoma State University-CHS Institutional Review Board.

*Please keep this information sheet for your records. By completing and returning this questionnaire, you are agreeing to participate in this study*.

### Appendix B – Microbial Jeopardy Evaluation Survey

Dear Student,

As a first year student, you took the *Medical Microbiology and Immunology *course. During that course an interactive session entitled "Microbial Jeopardy" was presented. Please answer the following questions based on your experiences during the Microbial Jeopardy session.

In what semester/year did you take the *Medical Microbiology and Immunology *course? Spring __

**Please use the following scale to answer questions 1–8**. (see Table 3)

**Table 3:** Microbial Jeopardy Evaluation Survey

	**Strongly** **Disagree**	**Disagree**	**Agree**	**Strongly** **Agree**	**Not Applicable**
1. I enjoyed the Microbial Jeopardy session.	1	2	3	4	N/A
2. I would like to see similar sessions incorporated into other classes.	1	2	3	4	N/A
3. The Microbial Jeopardy session held my my interest.	1	2	3	4	N/A
4. The Microbial Jeopardy session was a useful way to prepare for the exam.	1	2	3	4	N/A
5. The case-based exam questions Prepared me to answer the case-based questions on the exam.	1	2	3	4	N/A
6. The cases presented in Microbial Jeopardy were realistic.	1	2	3	4	N/A
7. The case-based nature of the Microbial Jeopardy session helped to improve my ability to answer case-based exam questions in other classes.	1	2	3	4	N/A
8. Using case-based questions helps the application of knowledge in clinical courses.	1	2	3	4	N/A
9. What percent of lectures did you attend in the *Medical Microbiology and Immunology *course?	0–20	21–40	41–60	61–80	81–100
10. My course grade was an	A	B	C	U	Decline to Answer

To ensure anonymity, please return completed surveys to the Office of Educational Development

## Pre-publication history

The pre-publication history for this paper can be accessed here:



## Supplementary Material

Additional file 1 **Microbial Jeopardy**. One round of Microbial Jeopardy! slides and cases are provided in the attached PowerPoint file. Multiple rounds of cases were used for the 2006 and 2007 course sessions. Click here for file

## References

[B1] Baker N, Verran J (2004). The future of microbiology laboratory classes–wet, dry or in combination?. Nat Rev Microbiol.

[B2] Kopp V, Stark R, Fischer MR (2008). Fostering diagnostic knowledge through computer-supported, case-based worked examples: effects of erroneous examples and feedback. Med Educ.

[B3] Massonetto JC, Marcellini C, Assis PS, de Toledo SF (2004). Student responses to the introduction of case-based learning and practical activities into a theoretical obstetrics and gynaecology teaching programme. BMC Med Educ.

[B4] Patel VL, Arocha JF, Branch T, Karlin DR (2004). Relationship between small group problem-solving activity and lectures in health science curricula. J Dent Educ.

[B5] Wood DF (2003). Problem based learning. BMJ.

[B6] Gimpel JR (2007). Getting "beyond the barriers" in reforming osteopathic medical education. J Am Osteopath Assoc.

[B7] MacKinnon GE (2000). Preparing medical students for the changing healthcare environment in United States. J Am Osteopath Assoc.

[B8] Vigna J Microbial Jeopardy!™ Review and Assessment. http://www.microbelibrary.org.

[B9] Cronbach LJ (1951). Coefficient alpha and the internal structure of tests. Psychometrika.

[B10] Lind DA, Marchal WG, Wathen SA (2008). Statistical techniques in business and economics.

[B11] Dommeyer CJ, Baum P, Hanna RW, Chapman KS (2004). Gathering faculty teaching evaluations by in-class and online surveys: their effects on response rates and evaluations. Assessment & Evaluation in Higher Education.

[B12] Ochsendorf FR, Boehncke WH, Sommerlad M, Kaufmann R (2006). Interactive large-group teaching in a dermatology course. Med Teach.

[B13] Steinberg DM, Chan TY, Freedman JA, Grimm LA, Ling L, Lehmann HP, Burroughs FH, Rosenthal DL, Ali SZ (2002). Teaching cytopathology to second-year medical students. An interactive, case-based approach. Acta Cytol.

[B14] Wahlgren CF, Edelbring S, Fors U, Hindbeck H, Stahle M (2006). Evaluation of an interactive case simulation system in dermatology and venereology for medical students. BMC Med Educ.

[B15] Tarnvik A (2007). Revival of the case method: a way to retain student-centred learning in a post-PBL era. Med Teach.

