# Supplementary material for: Evaluation of an interactive, case-based review session in teaching medical microbiology
Source: BMC Med Educ. 2009 Aug 27;9:56. doi: 10.1186/1472-6920-9-56 (PMC2739197; doi:10.1186/1472-6920-9-56)
Supplement: Additional file 1 — Microbial Jeopardy. One round of Microbial Jeopardy! slides and cases are provided in the attached PowerPoint file. Multiple rounds of cases were used for the 2006 and 2007 course sessions. [file 1472-6920-9-56-S1.ppt]

## Slide 1
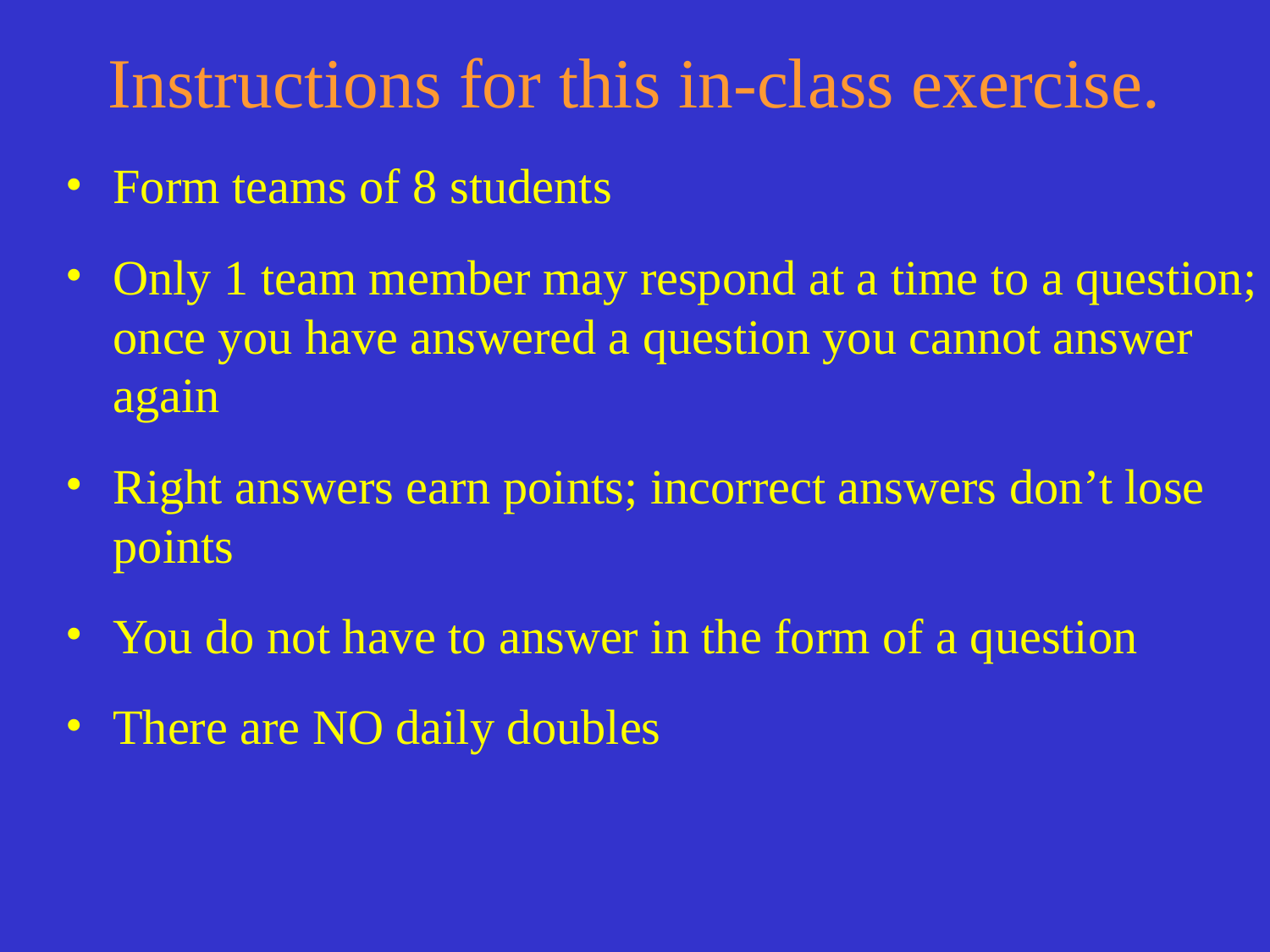

# Instructions for this in-class exercise.
Form teams of 8 students
Only 1 team member may respond at a time to a question; once you have answered a question you cannot answer again
Right answers earn points; incorrect answers don’t lose points
You do not have to answer in the form of a question
There are NO daily doubles

## Slide 2
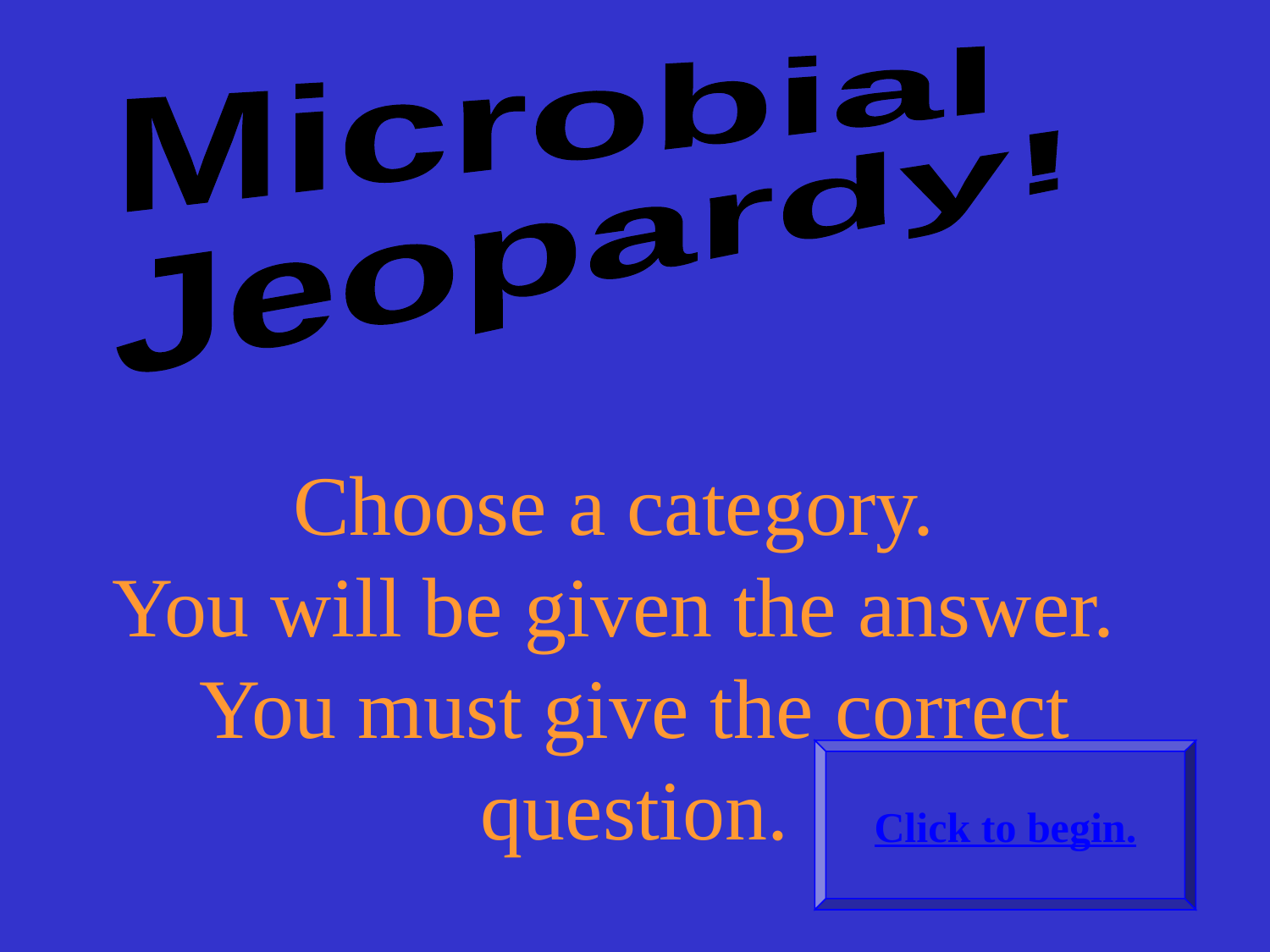

Microbial
Jeopardy!
Choose a category.
You will be given the answer.
You must give the correct question.
Click to begin.

## Slide 3
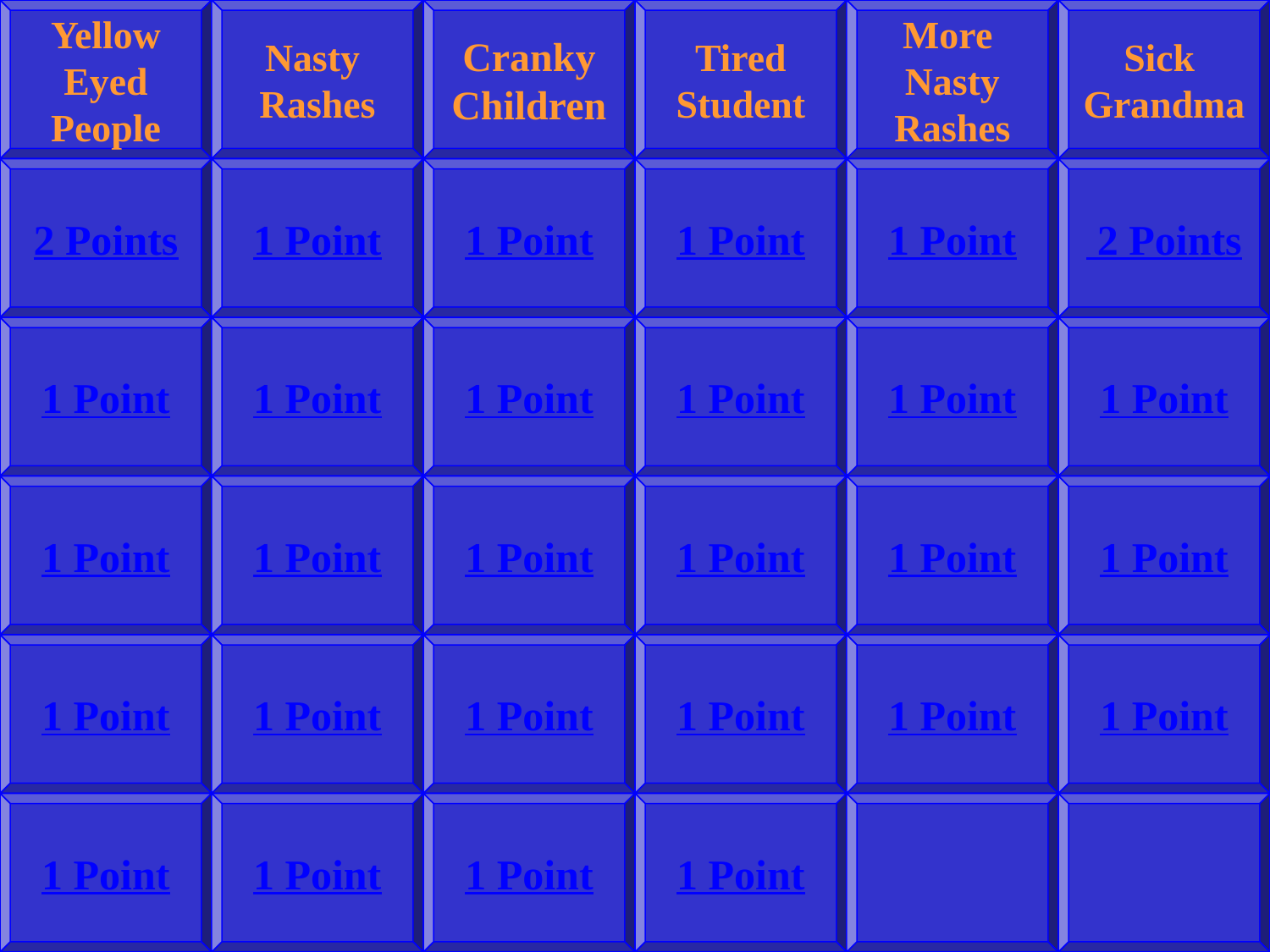

Yellow
Eyed
People
Nasty
Rashes
Cranky
Children
Tired
Student
More
Nasty
Rashes
Sick
Grandma
2 Points
1 Point
1 Point
1 Point
1 Point
 2 Points
1 Point
1 Point
1 Point
1 Point
1 Point
1 Point
1 Point
1 Point
1 Point
1 Point
1 Point
1 Point
1 Point
1 Point
1 Point
1 Point
1 Point
1 Point
1 Point
1 Point
1 Point
1 Point

## Slide 4
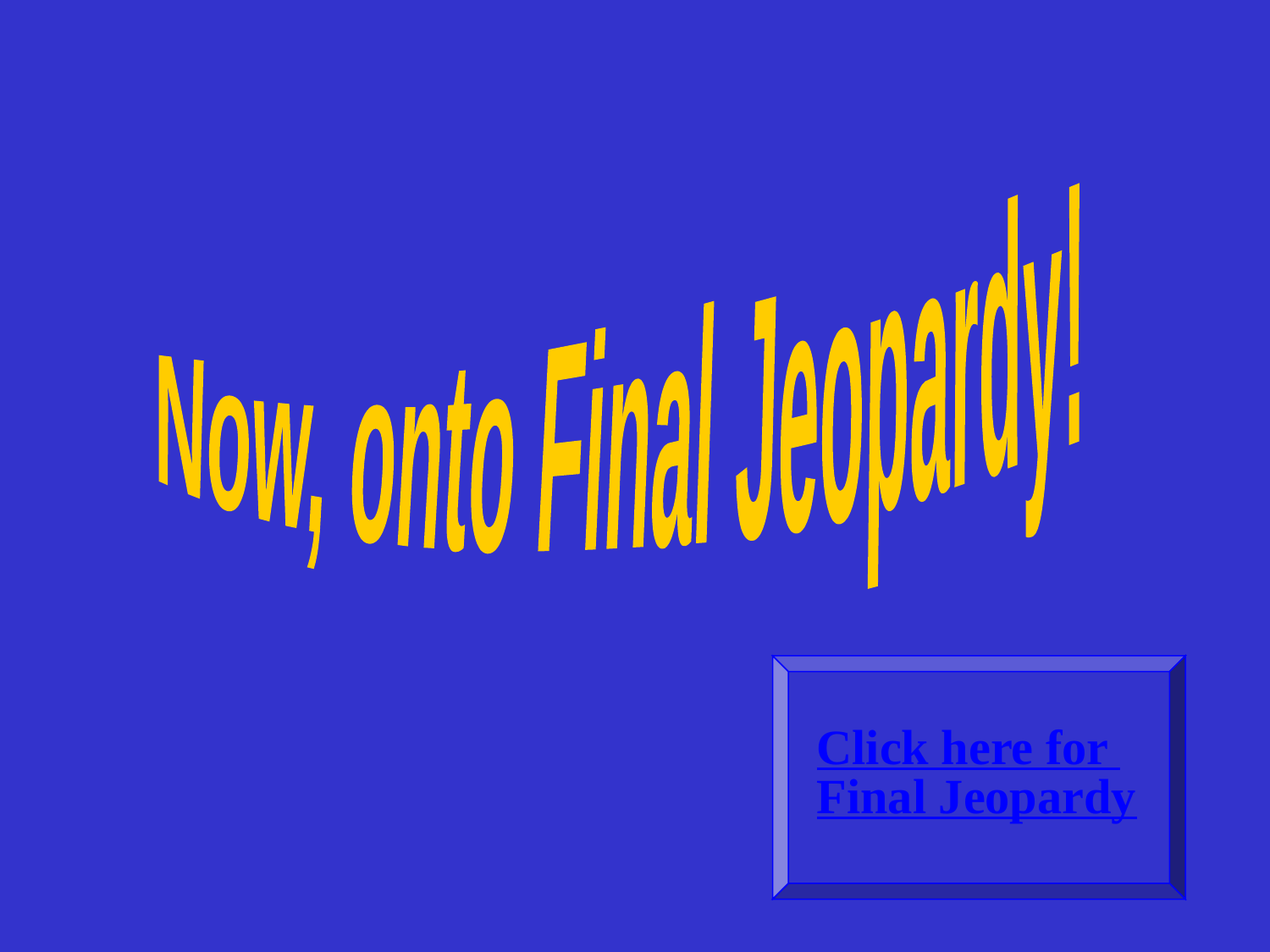

Now, onto Final Jeopardy!
Click here for Final Jeopardy

## Slide 5
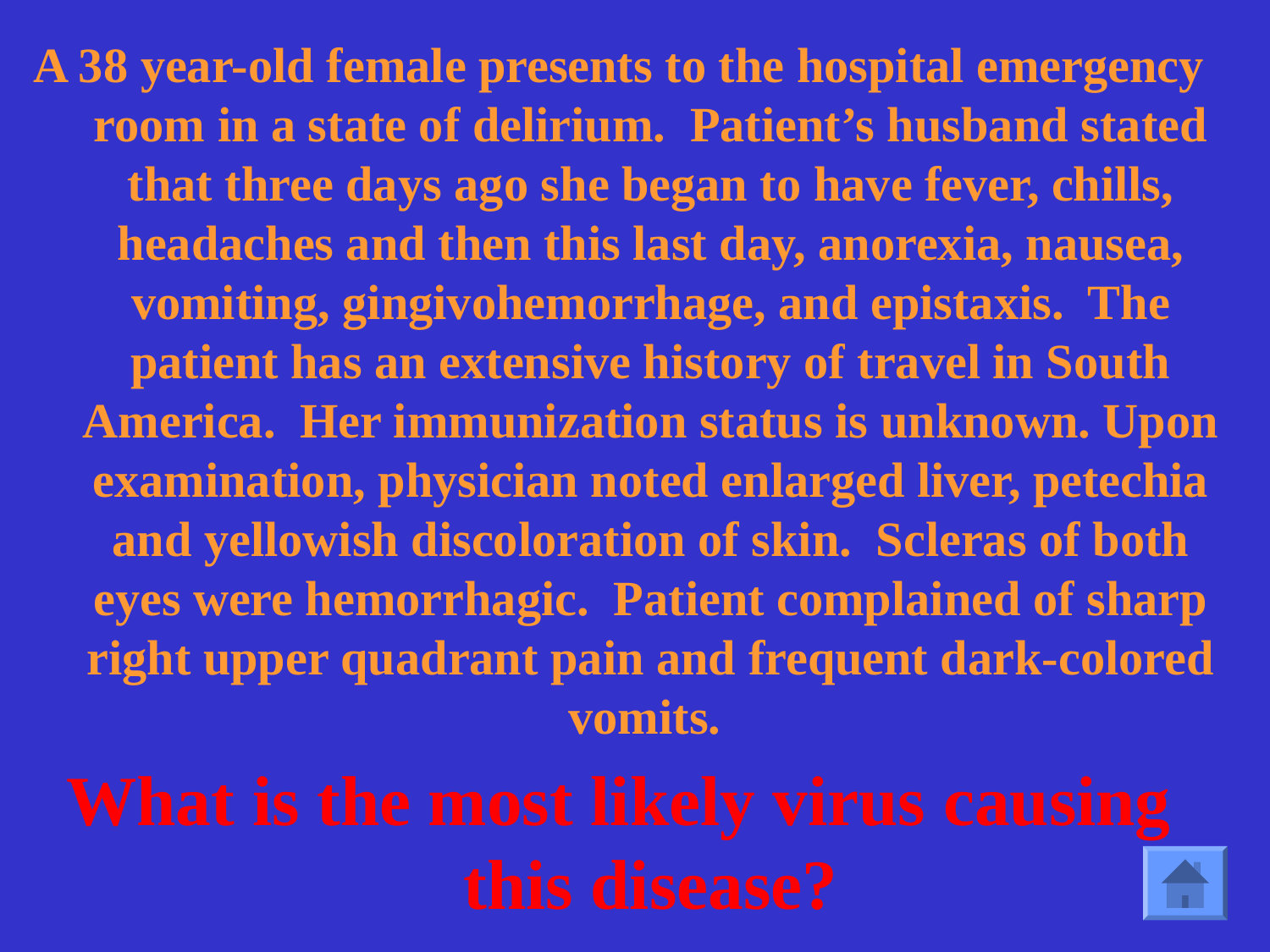

A 38 year-old female presents to the hospital emergency room in a state of delirium. Patient’s husband stated that three days ago she began to have fever, chills, headaches and then this last day, anorexia, nausea, vomiting, gingivohemorrhage, and epistaxis. The patient has an extensive history of travel in South America. Her immunization status is unknown. Upon examination, physician noted enlarged liver, petechia and yellowish discoloration of skin. Scleras of both eyes were hemorrhagic. Patient complained of sharp right upper quadrant pain and frequent dark-colored vomits.
What is the most likely virus causing this disease?

## Slide 6
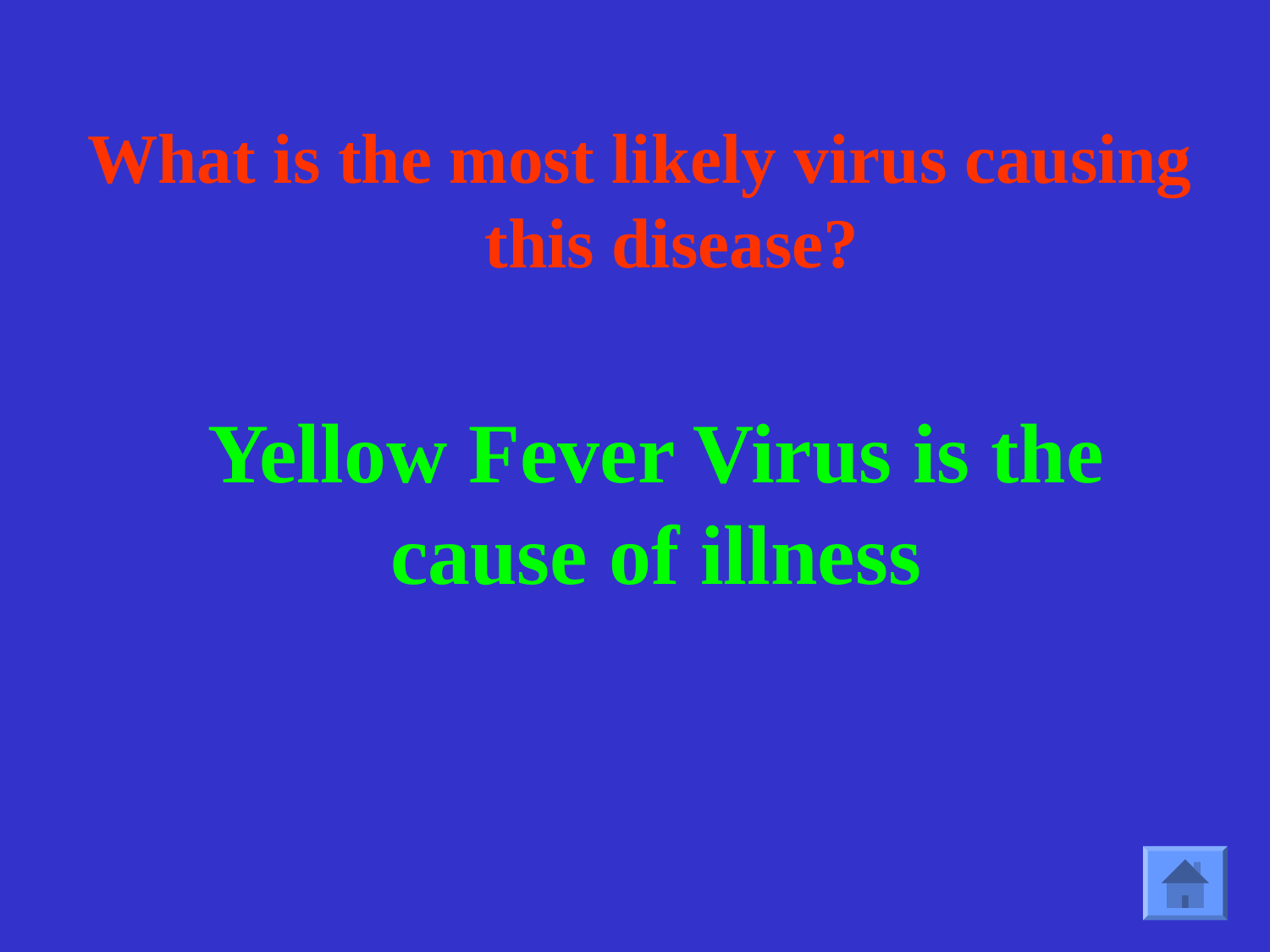

What is the most likely virus causing this disease?
Yellow Fever Virus is the cause of illness

## Slide 7
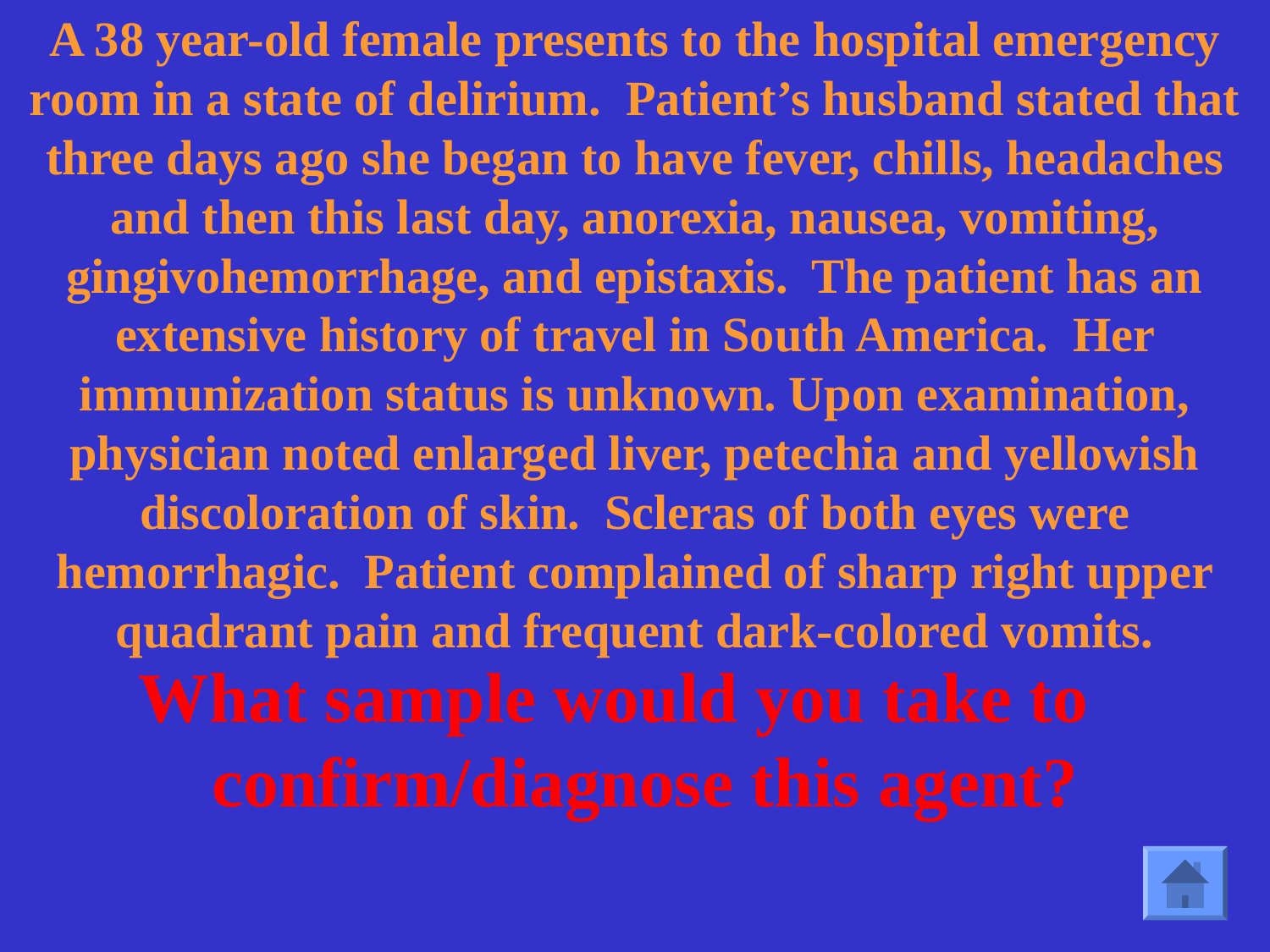

A 38 year-old female presents to the hospital emergency room in a state of delirium. Patient’s husband stated that three days ago she began to have fever, chills, headaches and then this last day, anorexia, nausea, vomiting, gingivohemorrhage, and epistaxis. The patient has an extensive history of travel in South America. Her immunization status is unknown. Upon examination, physician noted enlarged liver, petechia and yellowish discoloration of skin. Scleras of both eyes were hemorrhagic. Patient complained of sharp right upper quadrant pain and frequent dark-colored vomits.
What sample would you take to confirm/diagnose this agent?

## Slide 8
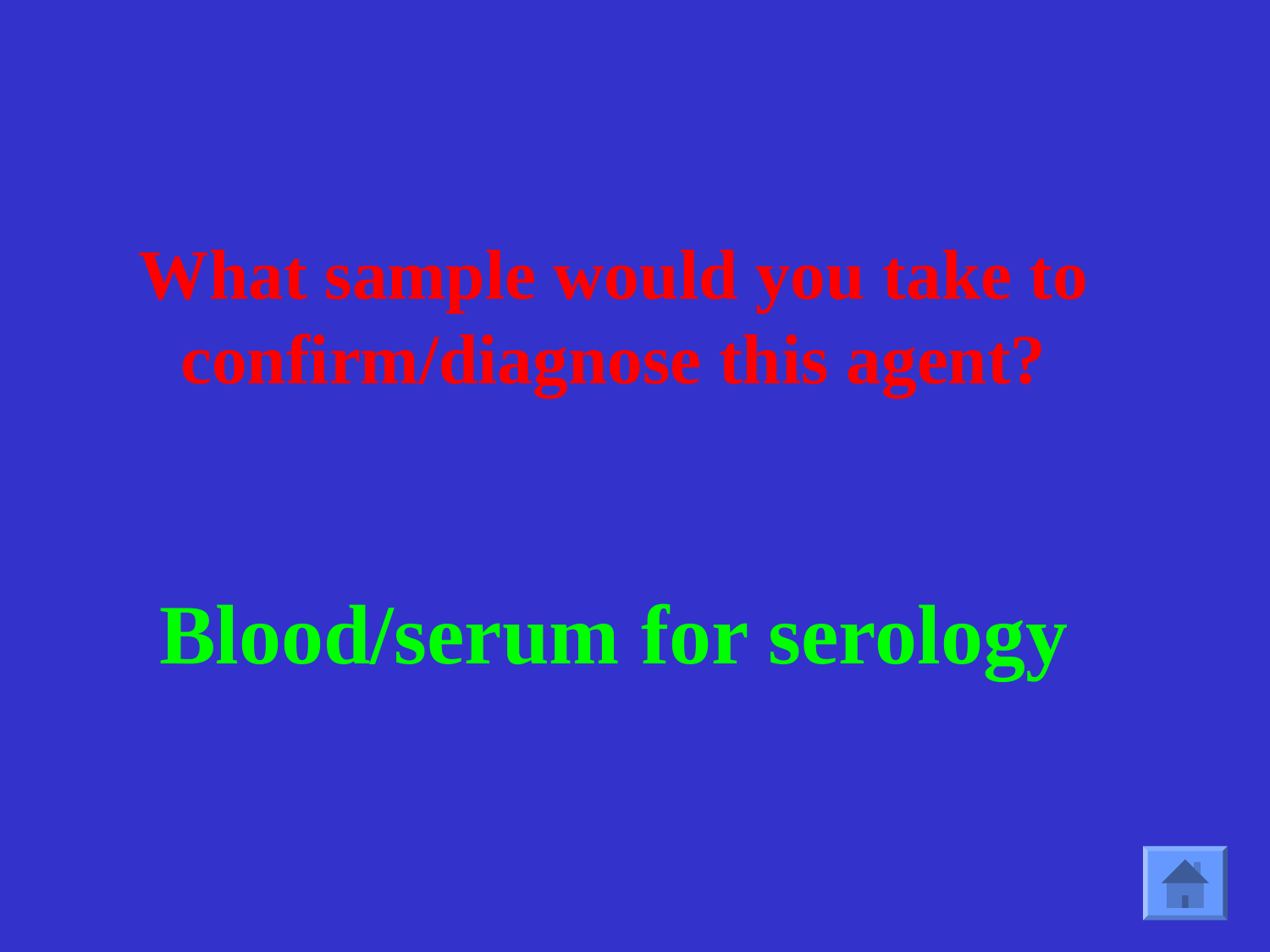

What sample would you take to confirm/diagnose this agent?
Blood/serum for serology

## Slide 9
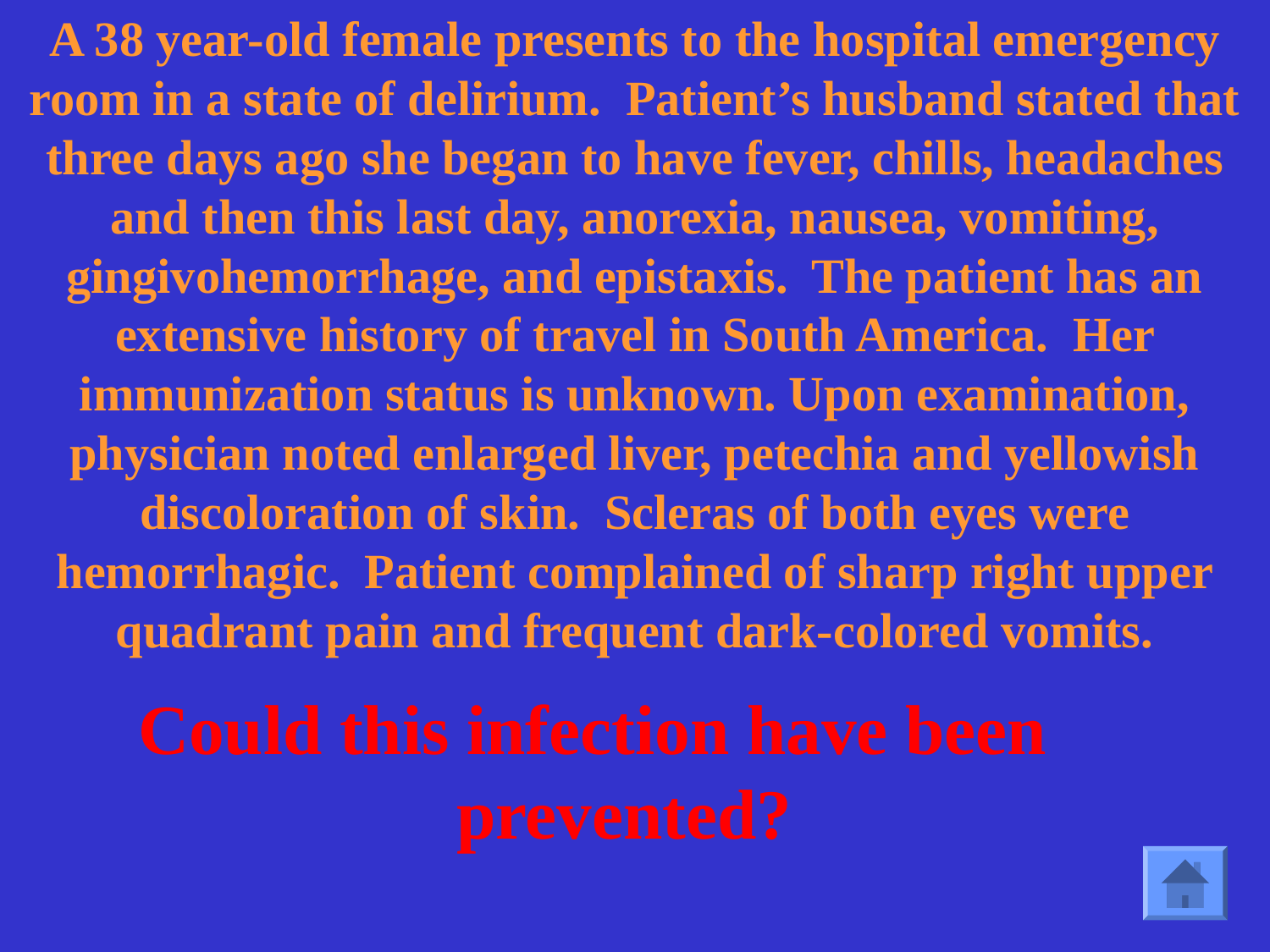

A 38 year-old female presents to the hospital emergency room in a state of delirium. Patient’s husband stated that three days ago she began to have fever, chills, headaches and then this last day, anorexia, nausea, vomiting, gingivohemorrhage, and epistaxis. The patient has an extensive history of travel in South America. Her immunization status is unknown. Upon examination, physician noted enlarged liver, petechia and yellowish discoloration of skin. Scleras of both eyes were hemorrhagic. Patient complained of sharp right upper quadrant pain and frequent dark-colored vomits.
Could this infection have been prevented?

## Slide 10
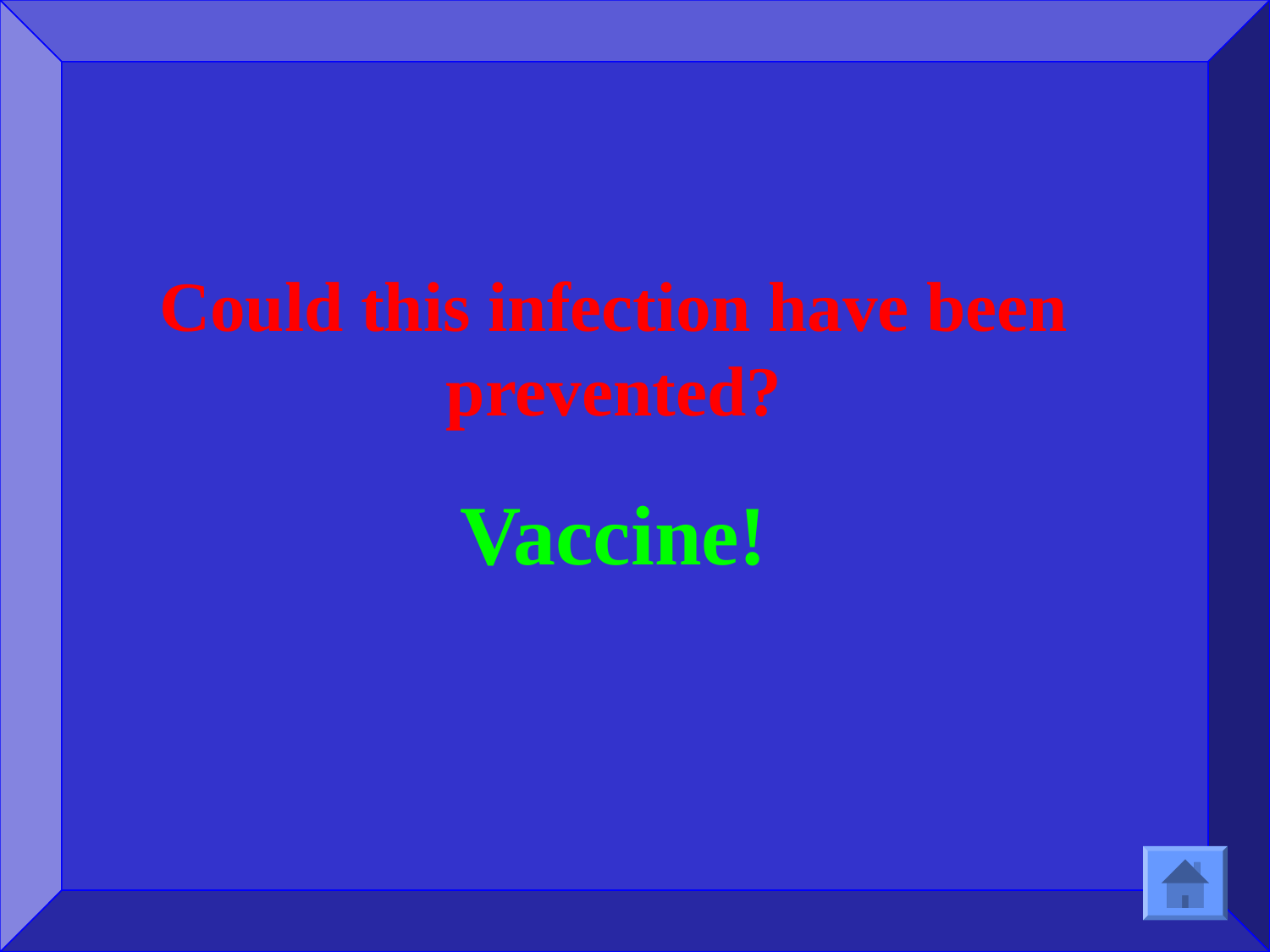

Could this infection have been prevented?
Vaccine!

## Slide 11
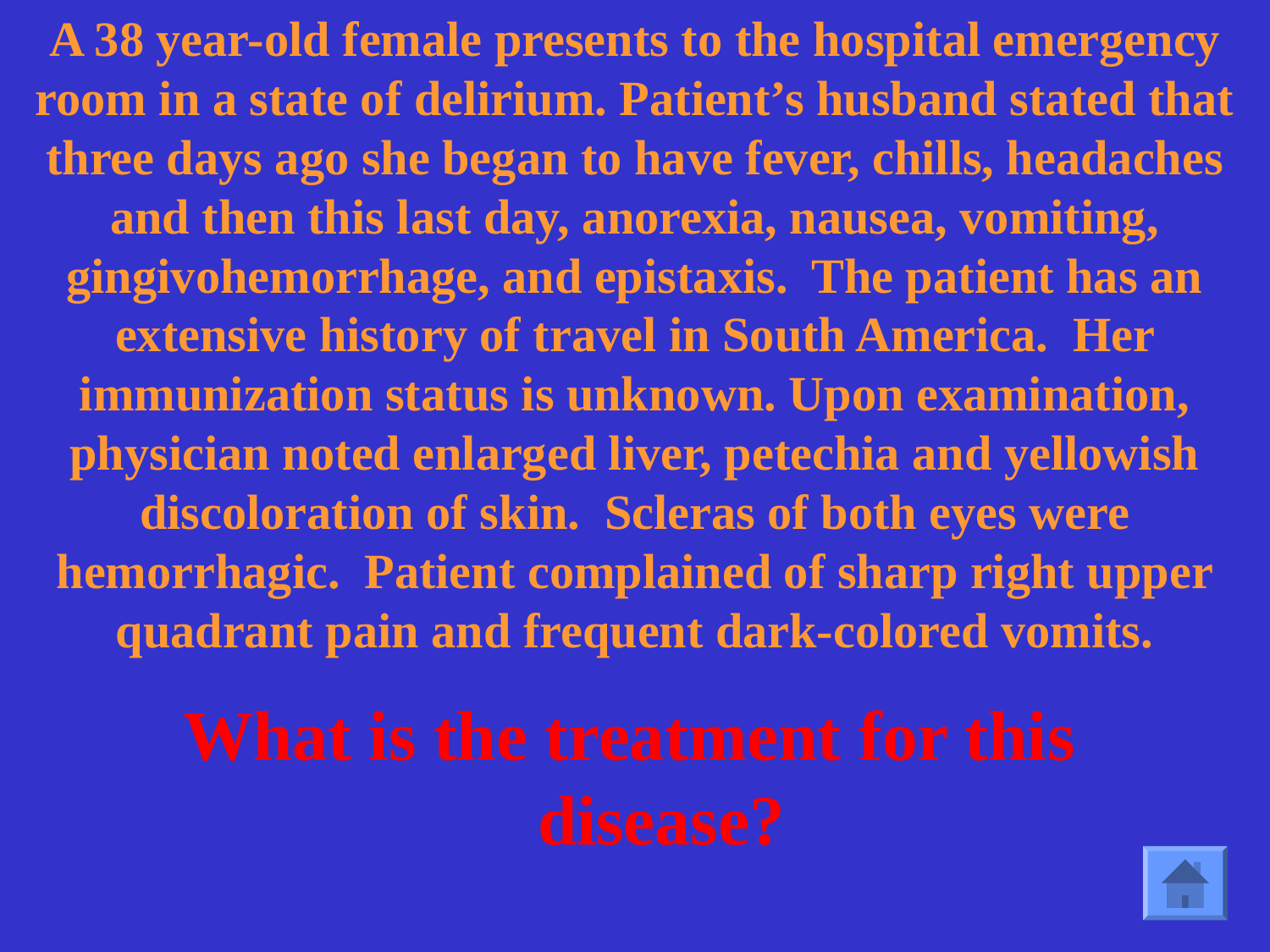

A 38 year-old female presents to the hospital emergency room in a state of delirium. Patient’s husband stated that three days ago she began to have fever, chills, headaches and then this last day, anorexia, nausea, vomiting, gingivohemorrhage, and epistaxis. The patient has an extensive history of travel in South America. Her immunization status is unknown. Upon examination, physician noted enlarged liver, petechia and yellowish discoloration of skin. Scleras of both eyes were hemorrhagic. Patient complained of sharp right upper quadrant pain and frequent dark-colored vomits.
What is the treatment for this disease?

## Slide 12
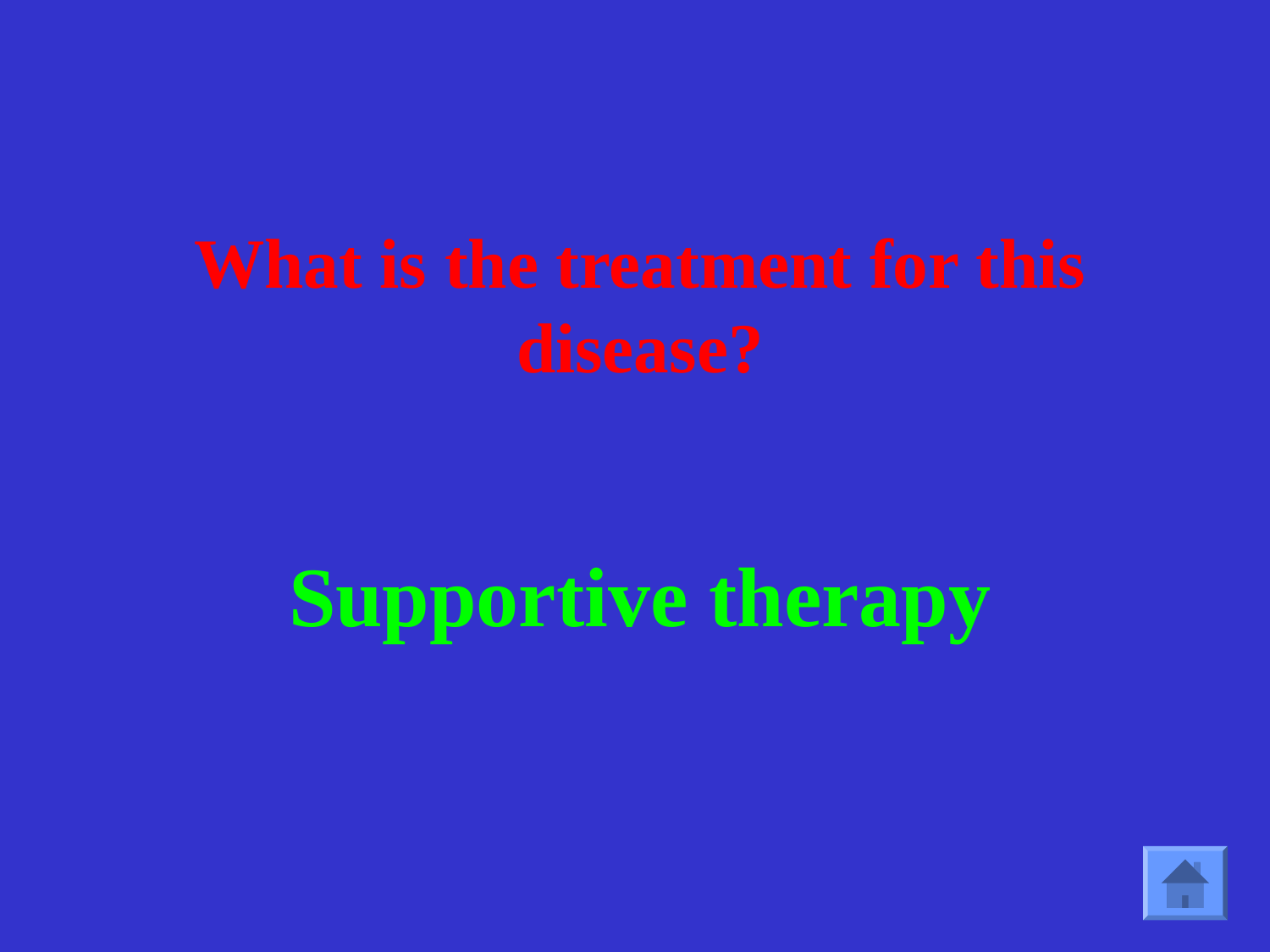

What is the treatment for this disease?
Supportive therapy

## Slide 13
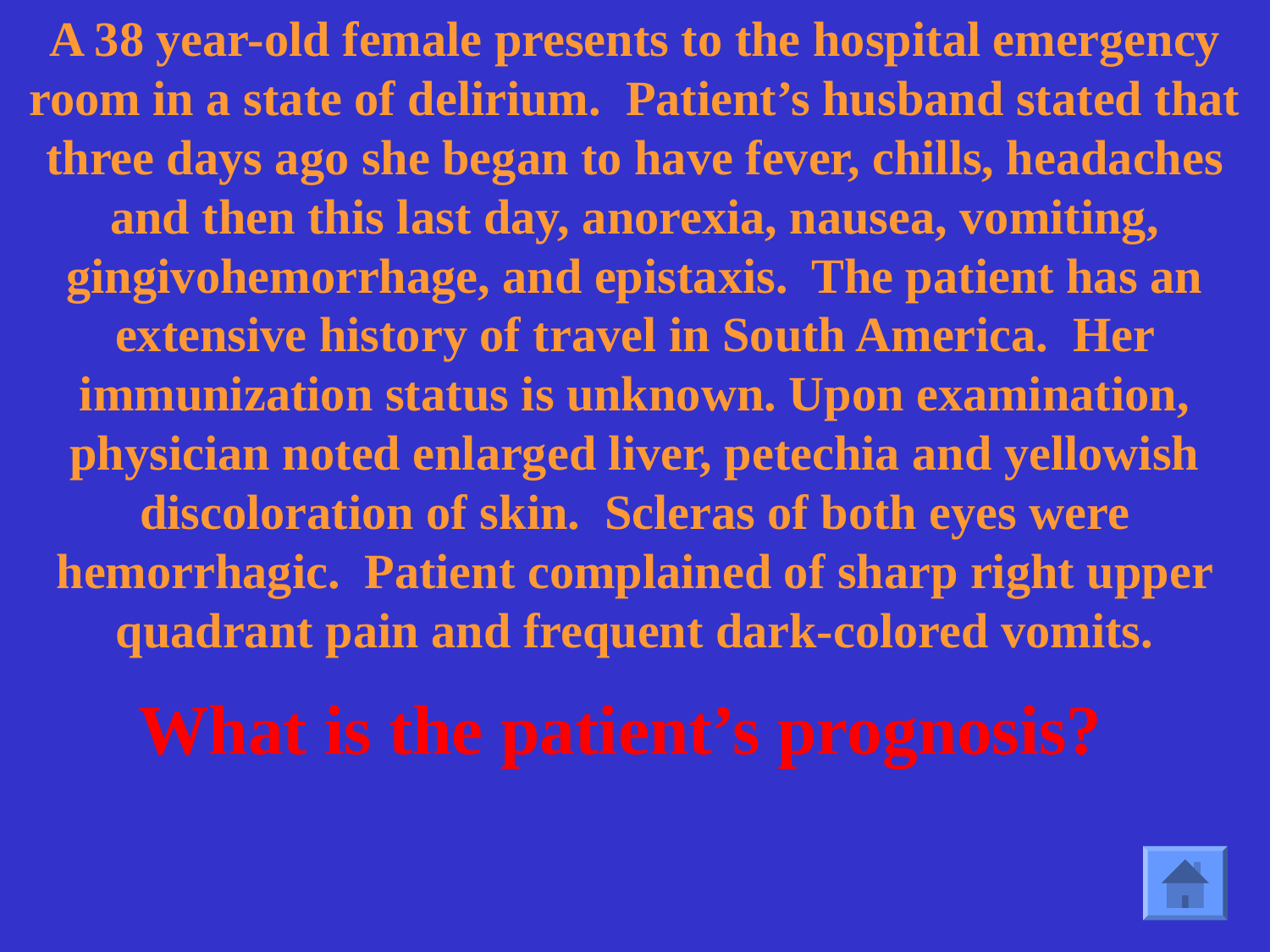

A 38 year-old female presents to the hospital emergency room in a state of delirium. Patient’s husband stated that three days ago she began to have fever, chills, headaches and then this last day, anorexia, nausea, vomiting, gingivohemorrhage, and epistaxis. The patient has an extensive history of travel in South America. Her immunization status is unknown. Upon examination, physician noted enlarged liver, petechia and yellowish discoloration of skin. Scleras of both eyes were hemorrhagic. Patient complained of sharp right upper quadrant pain and frequent dark-colored vomits.
What is the patient’s prognosis?

## Slide 14
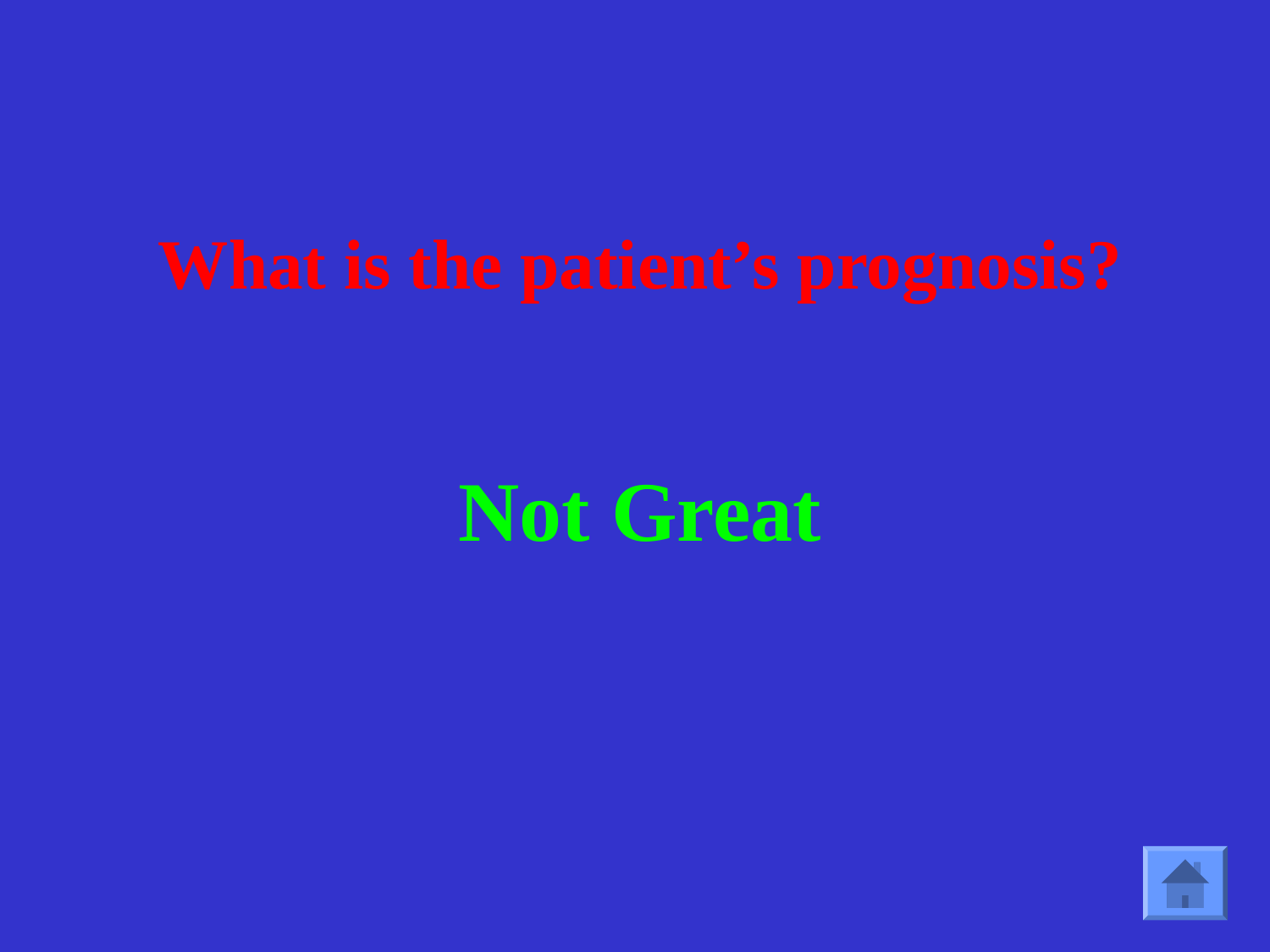

What is the patient’s prognosis?
Not Great

## Slide 15
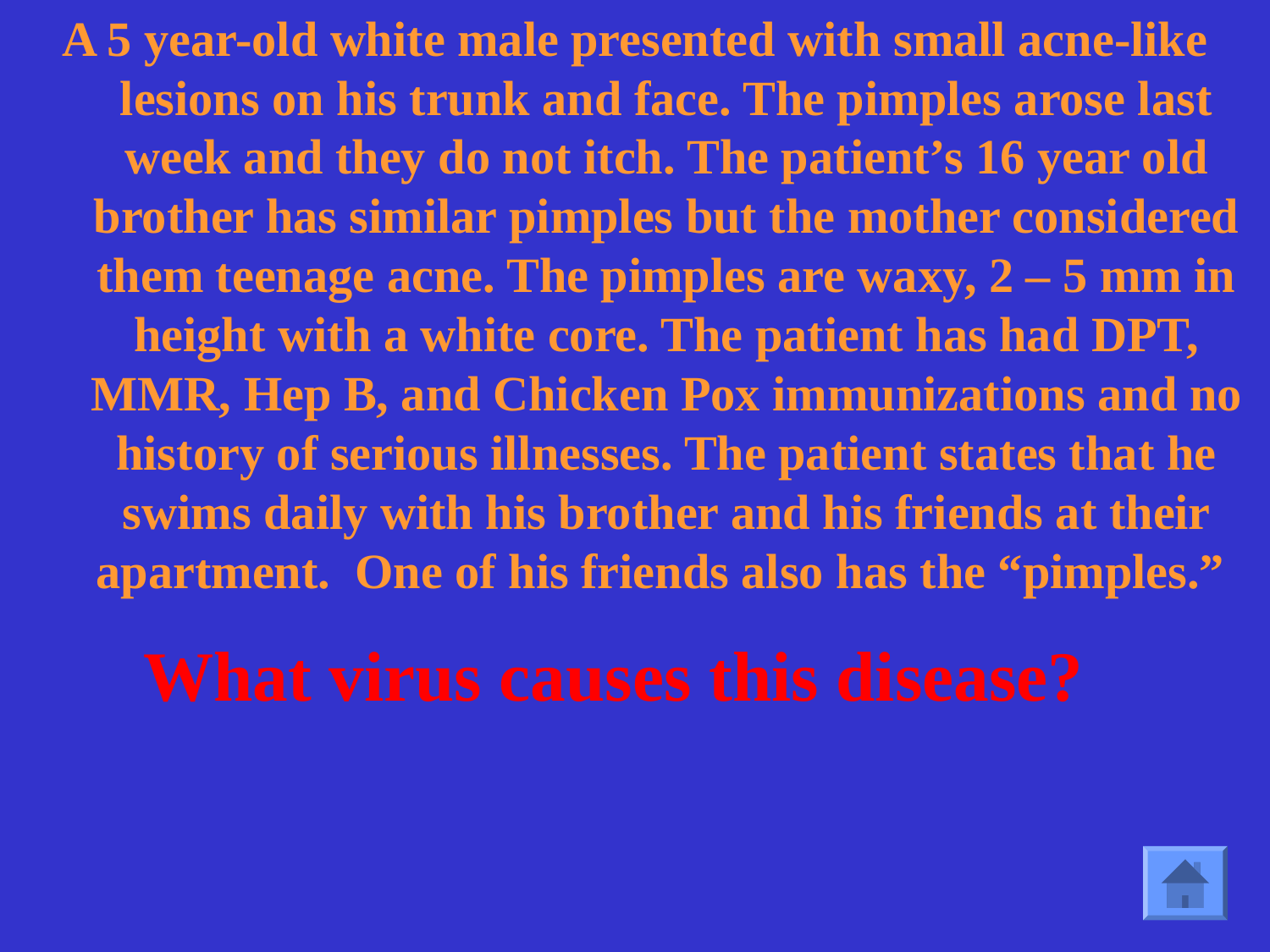

A 5 year-old white male presented with small acne-like lesions on his trunk and face. The pimples arose last week and they do not itch. The patient’s 16 year old brother has similar pimples but the mother considered them teenage acne. The pimples are waxy, 2 – 5 mm in height with a white core. The patient has had DPT, MMR, Hep B, and Chicken Pox immunizations and no history of serious illnesses. The patient states that he swims daily with his brother and his friends at their apartment. One of his friends also has the “pimples.”
What virus causes this disease?

## Slide 16
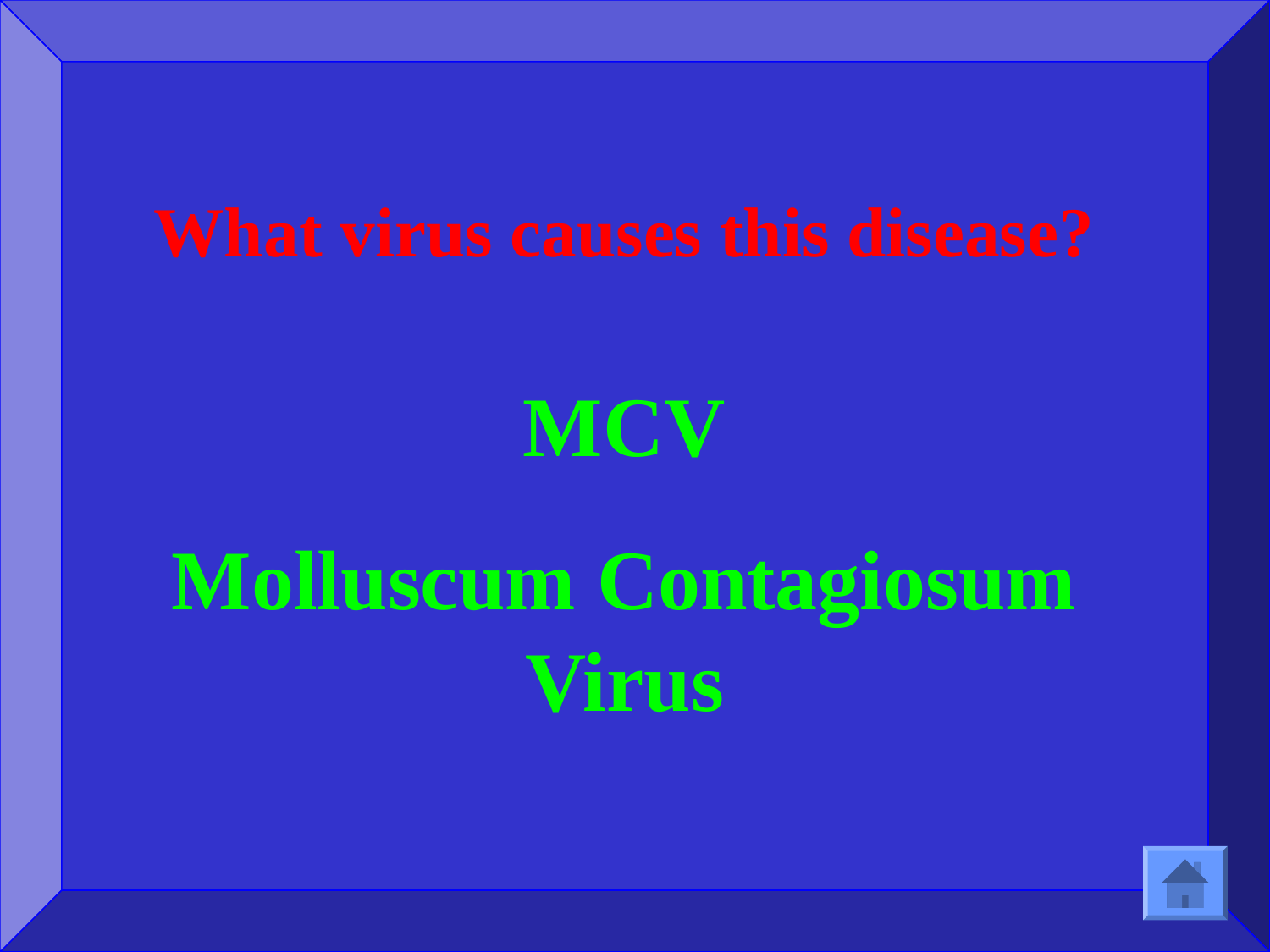

What virus causes this disease?
MCV
Molluscum Contagiosum Virus

## Slide 17
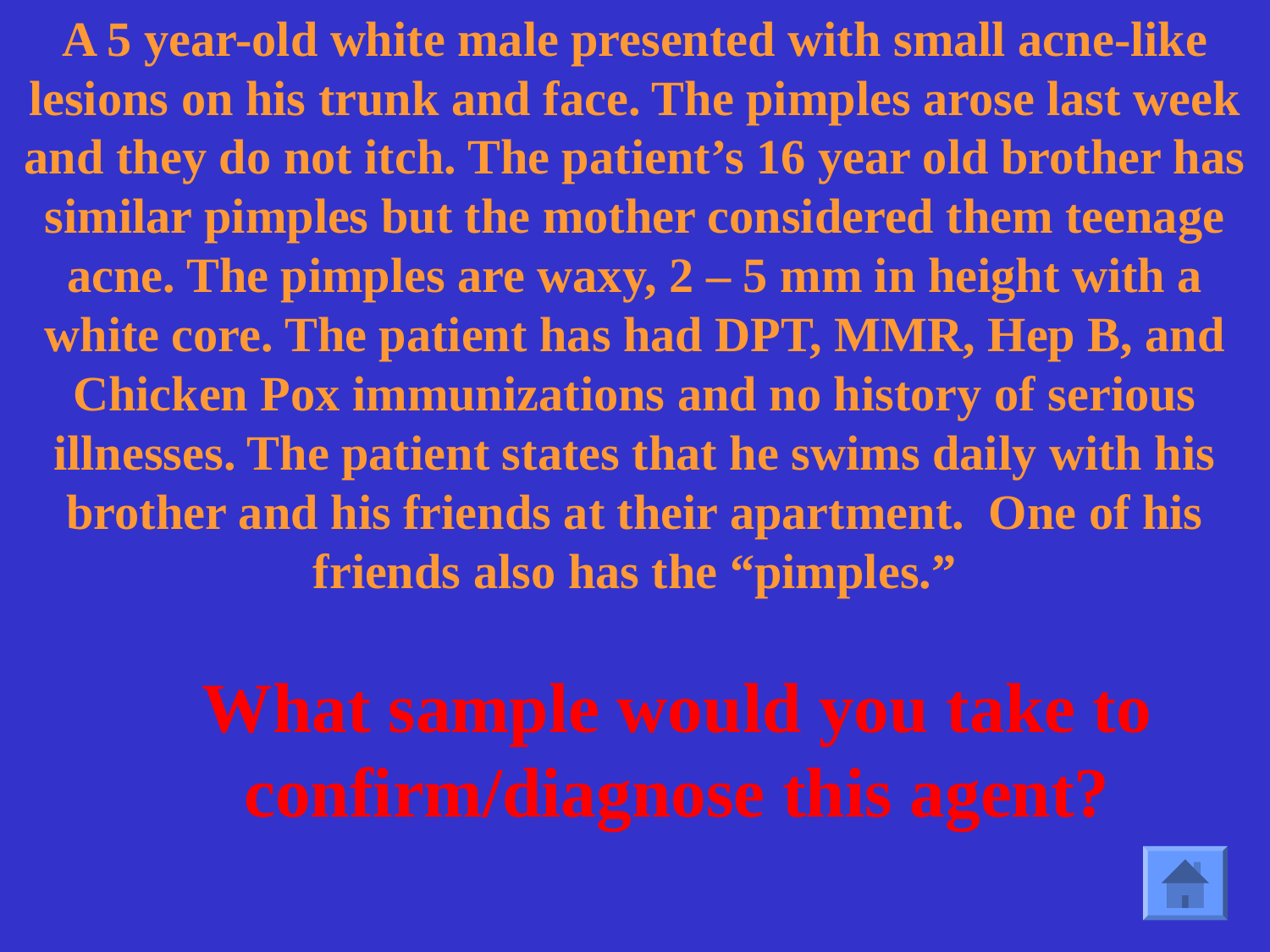

A 5 year-old white male presented with small acne-like lesions on his trunk and face. The pimples arose last week and they do not itch. The patient’s 16 year old brother has similar pimples but the mother considered them teenage acne. The pimples are waxy, 2 – 5 mm in height with a white core. The patient has had DPT, MMR, Hep B, and Chicken Pox immunizations and no history of serious illnesses. The patient states that he swims daily with his brother and his friends at their apartment. One of his friends also has the “pimples.”
	What sample would you take to confirm/diagnose this agent?

## Slide 18
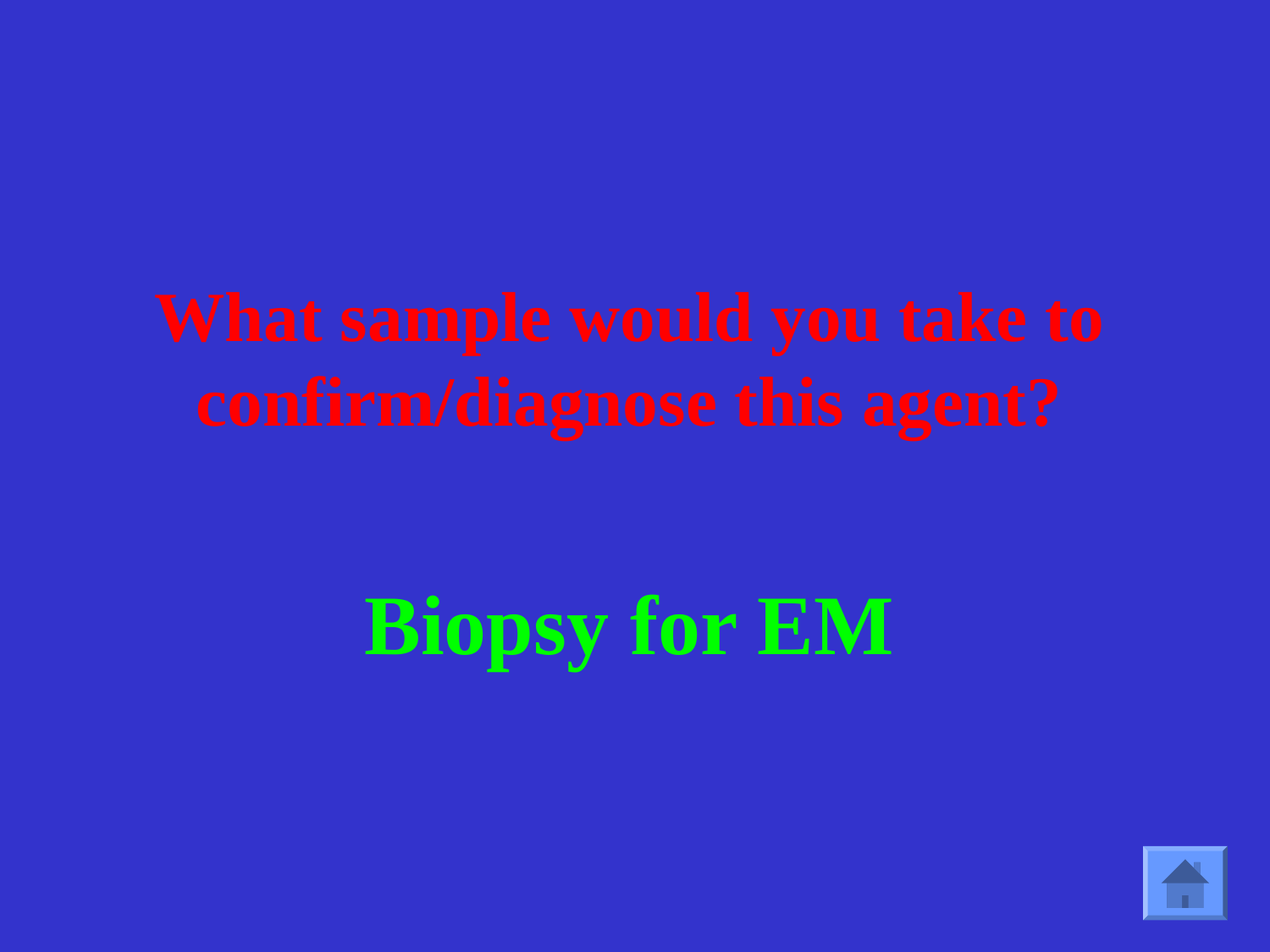

What sample would you take to confirm/diagnose this agent?
Biopsy for EM

## Slide 19
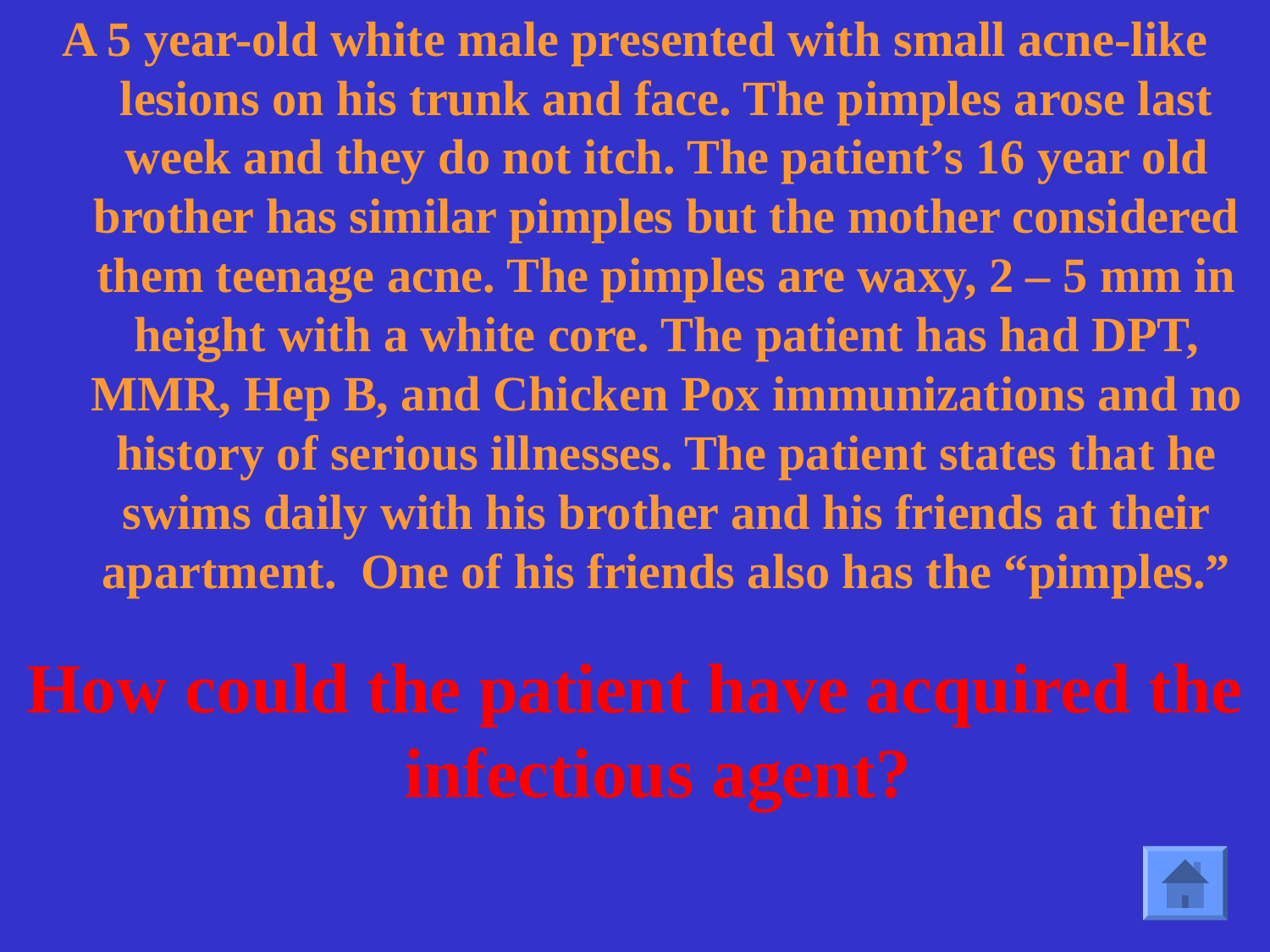

A 5 year-old white male presented with small acne-like lesions on his trunk and face. The pimples arose last week and they do not itch. The patient’s 16 year old brother has similar pimples but the mother considered them teenage acne. The pimples are waxy, 2 – 5 mm in height with a white core. The patient has had DPT, MMR, Hep B, and Chicken Pox immunizations and no history of serious illnesses. The patient states that he swims daily with his brother and his friends at their apartment. One of his friends also has the “pimples.”
How could the patient have acquired the infectious agent?

## Slide 20
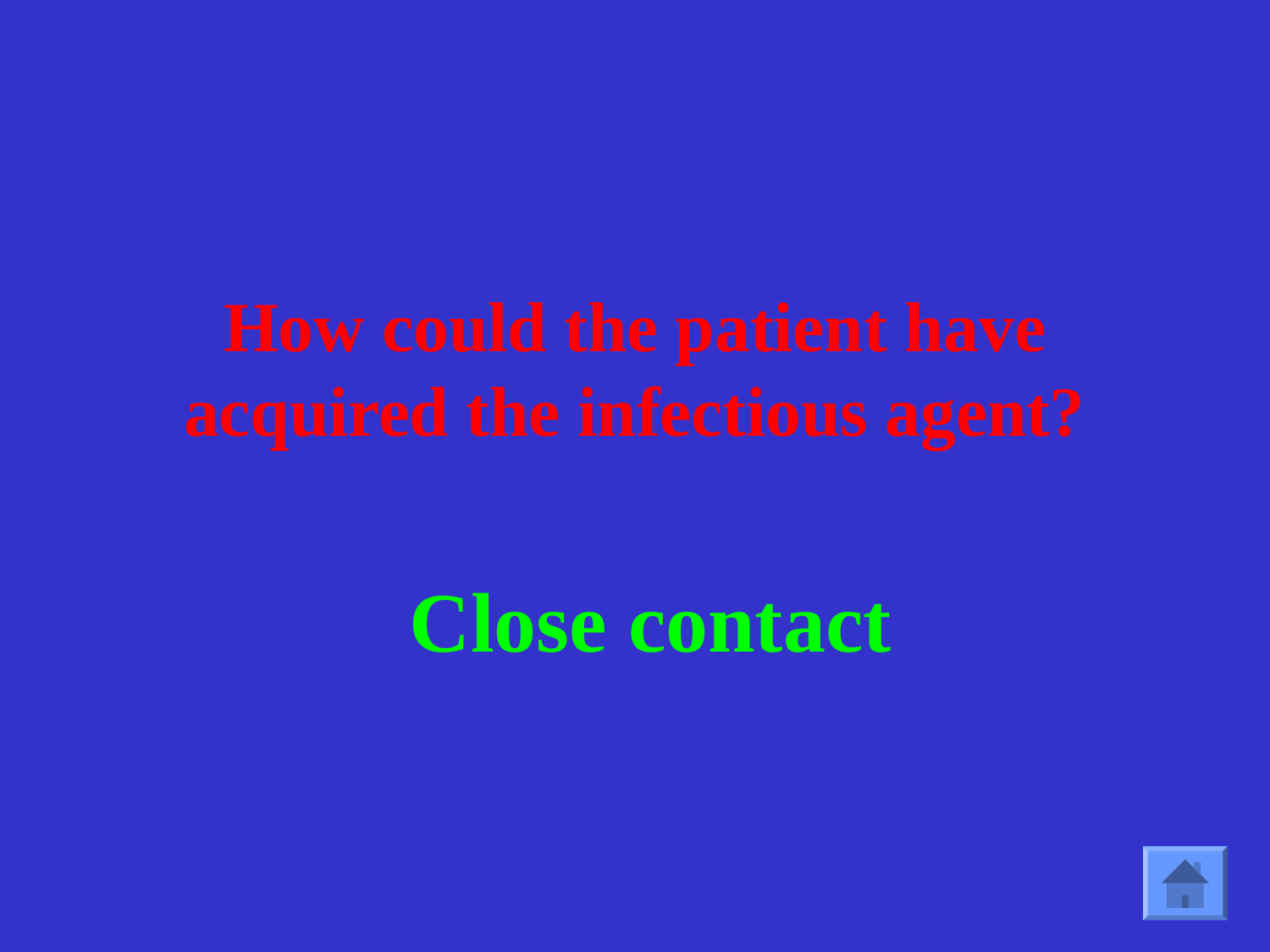

How could the patient have acquired the infectious agent?
Close contact

## Slide 21
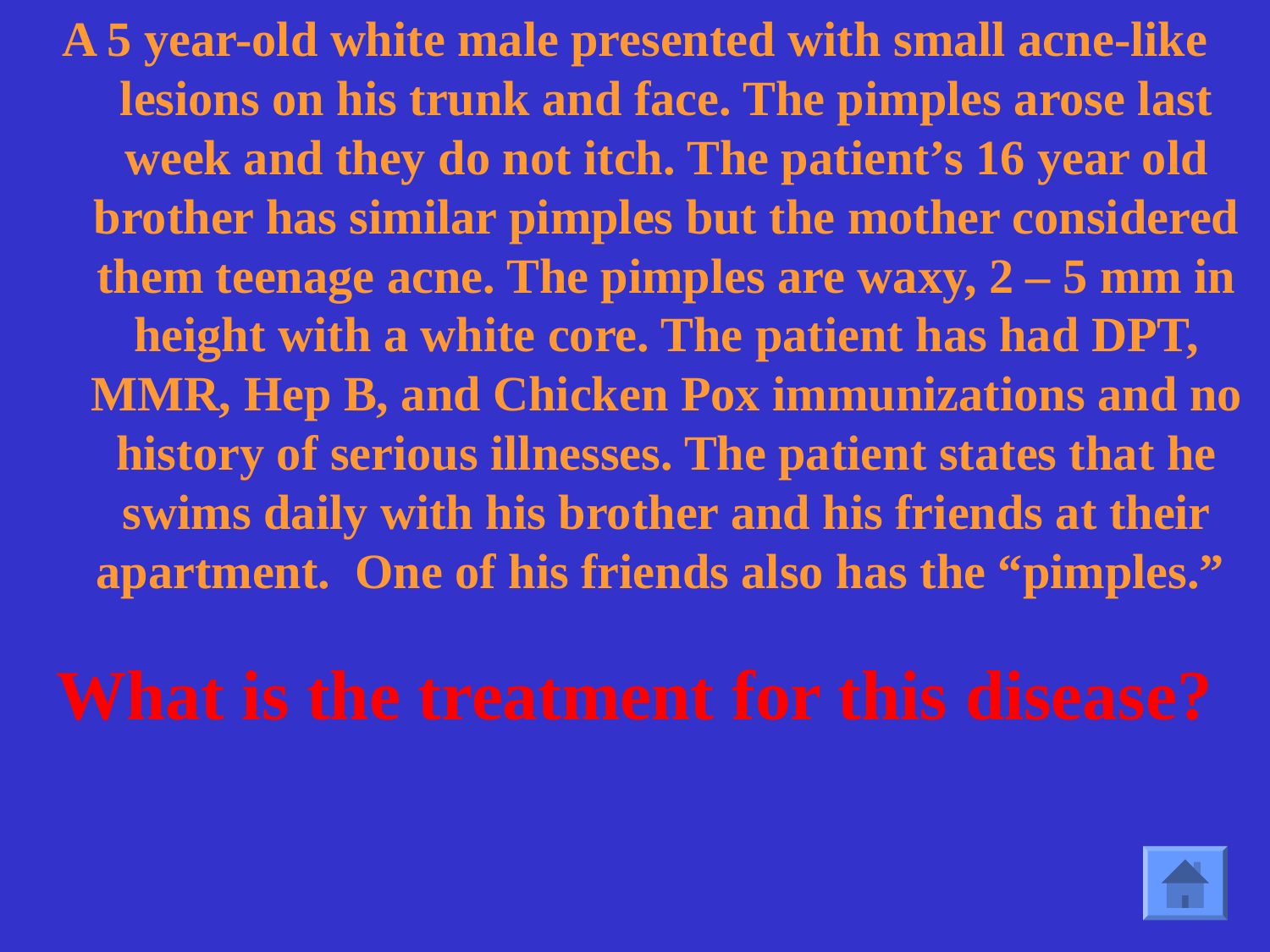

A 5 year-old white male presented with small acne-like lesions on his trunk and face. The pimples arose last week and they do not itch. The patient’s 16 year old brother has similar pimples but the mother considered them teenage acne. The pimples are waxy, 2 – 5 mm in height with a white core. The patient has had DPT, MMR, Hep B, and Chicken Pox immunizations and no history of serious illnesses. The patient states that he swims daily with his brother and his friends at their apartment. One of his friends also has the “pimples.”
What is the treatment for this disease?

## Slide 22
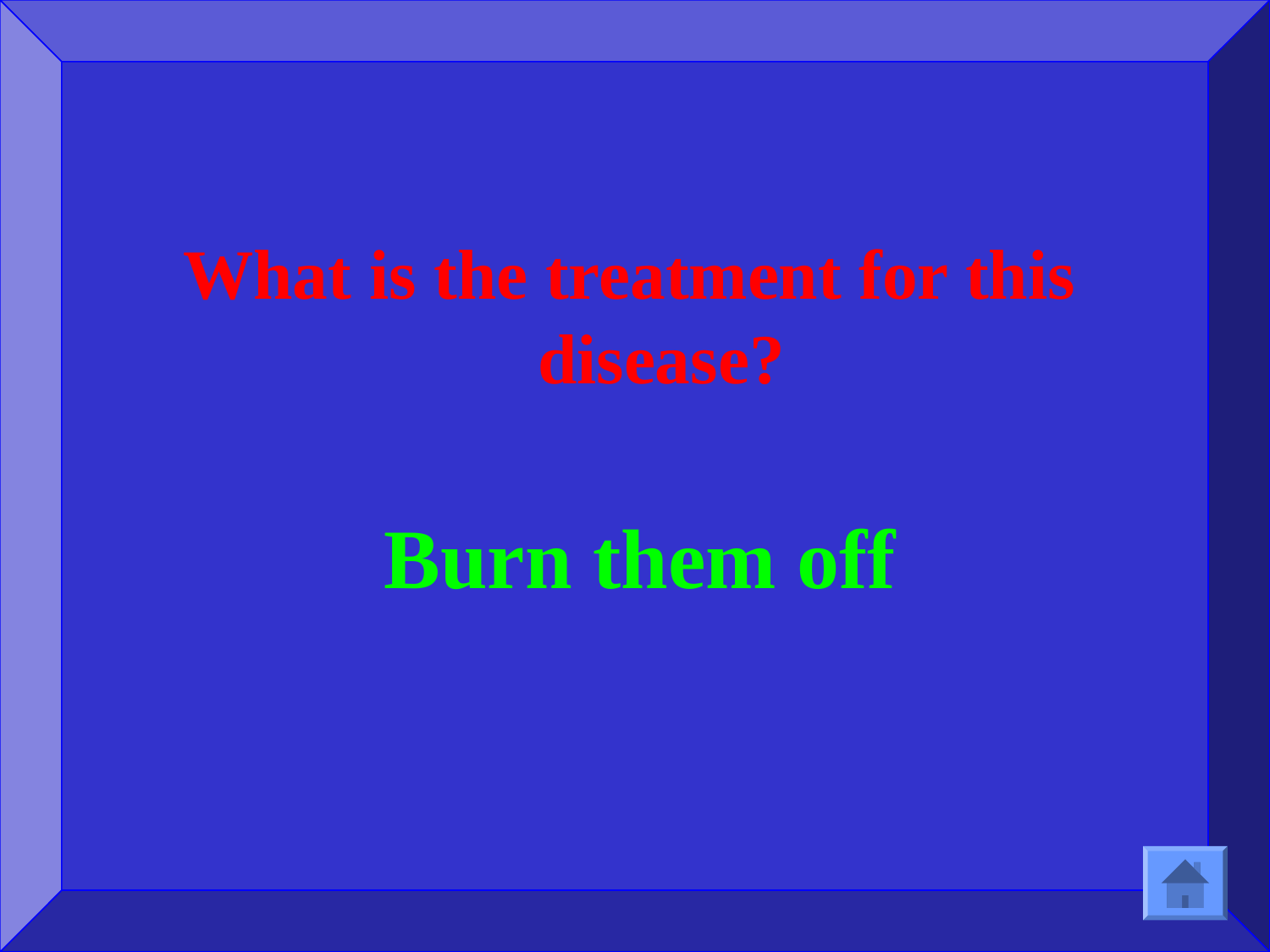

What is the treatment for this disease?
Burn them off

## Slide 23
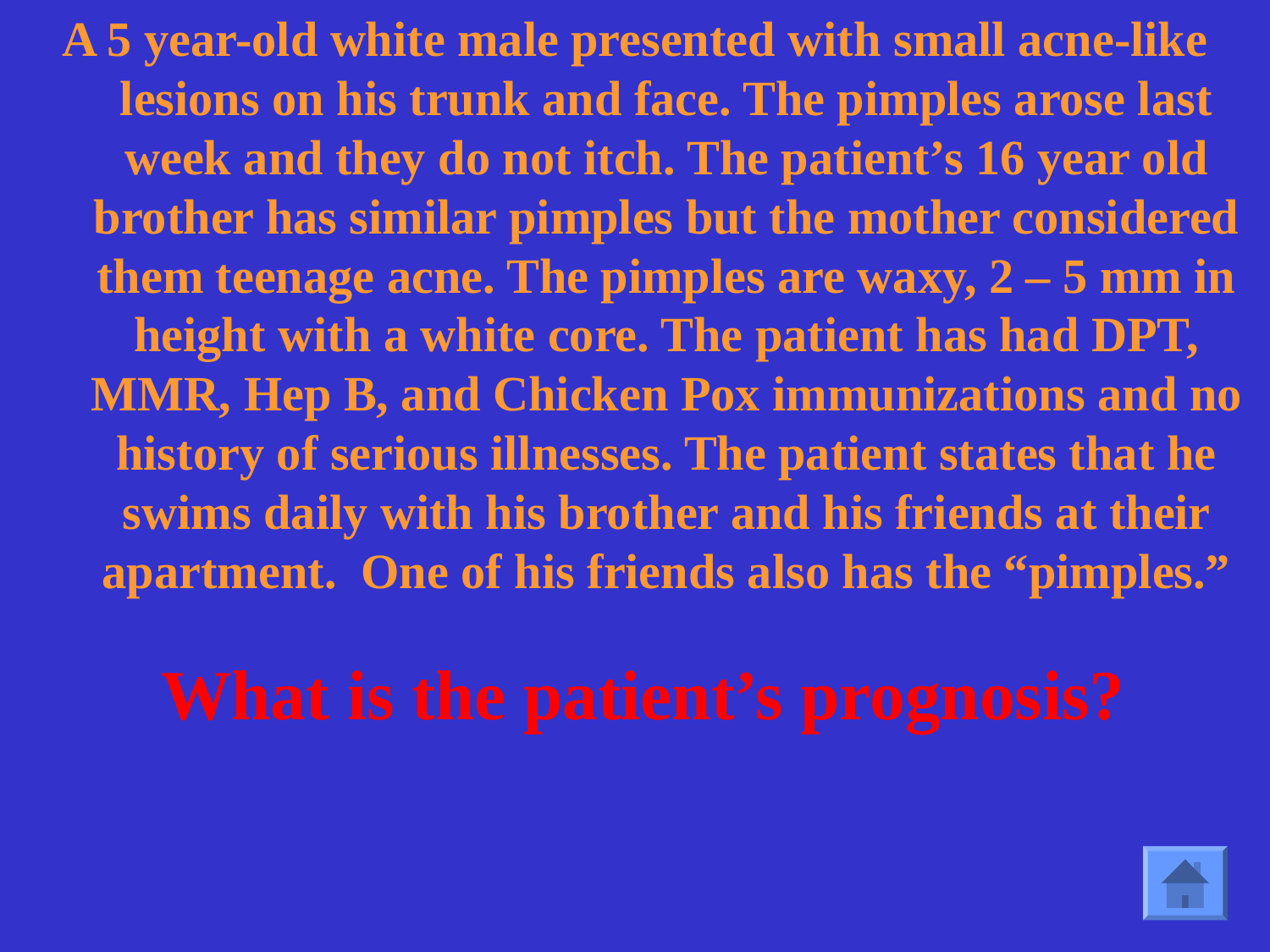

A 5 year-old white male presented with small acne-like lesions on his trunk and face. The pimples arose last week and they do not itch. The patient’s 16 year old brother has similar pimples but the mother considered them teenage acne. The pimples are waxy, 2 – 5 mm in height with a white core. The patient has had DPT, MMR, Hep B, and Chicken Pox immunizations and no history of serious illnesses. The patient states that he swims daily with his brother and his friends at their apartment. One of his friends also has the “pimples.”
 What is the patient’s prognosis?

## Slide 24
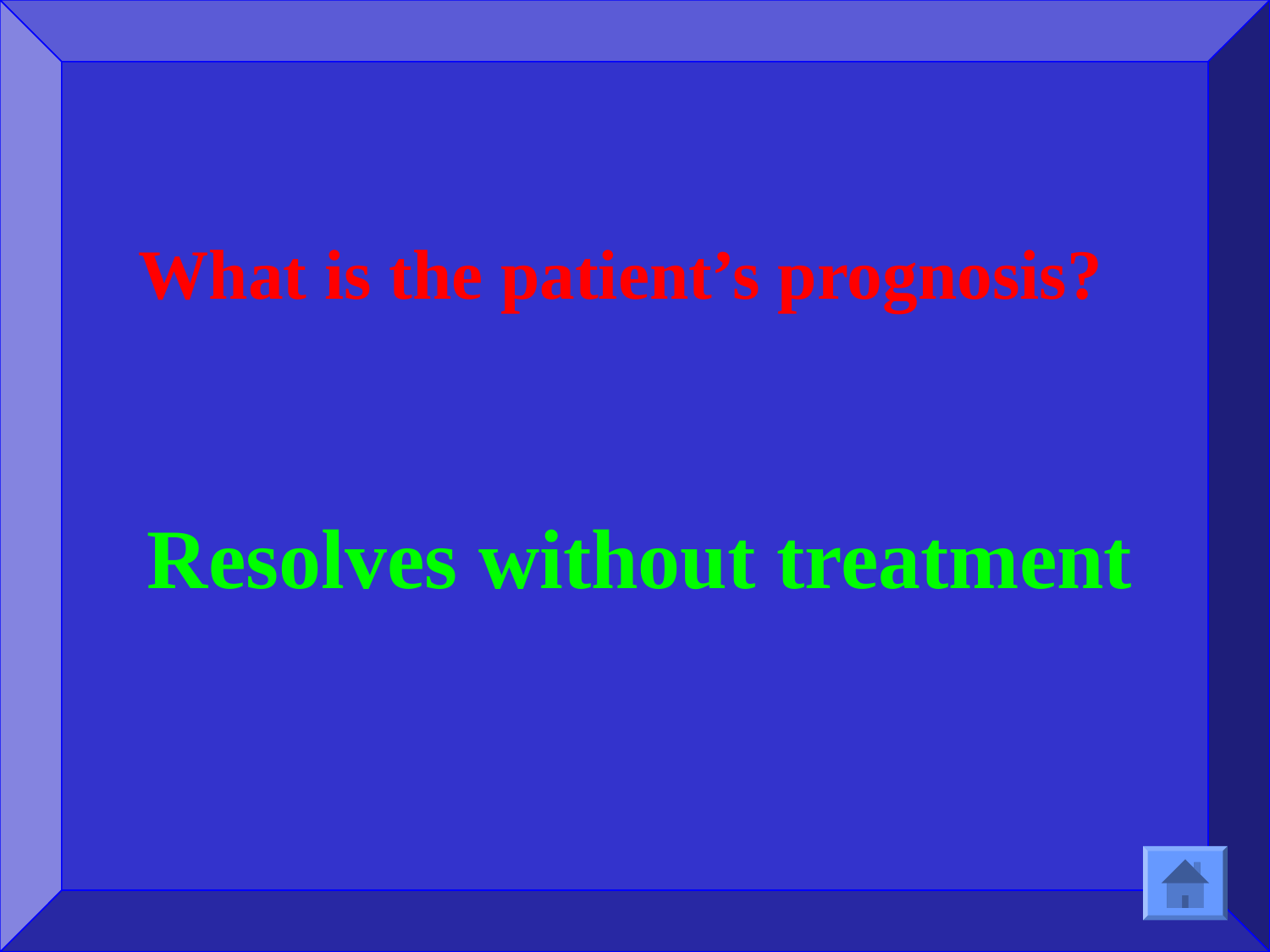

What is the patient’s prognosis?
Resolves without treatment

## Slide 25
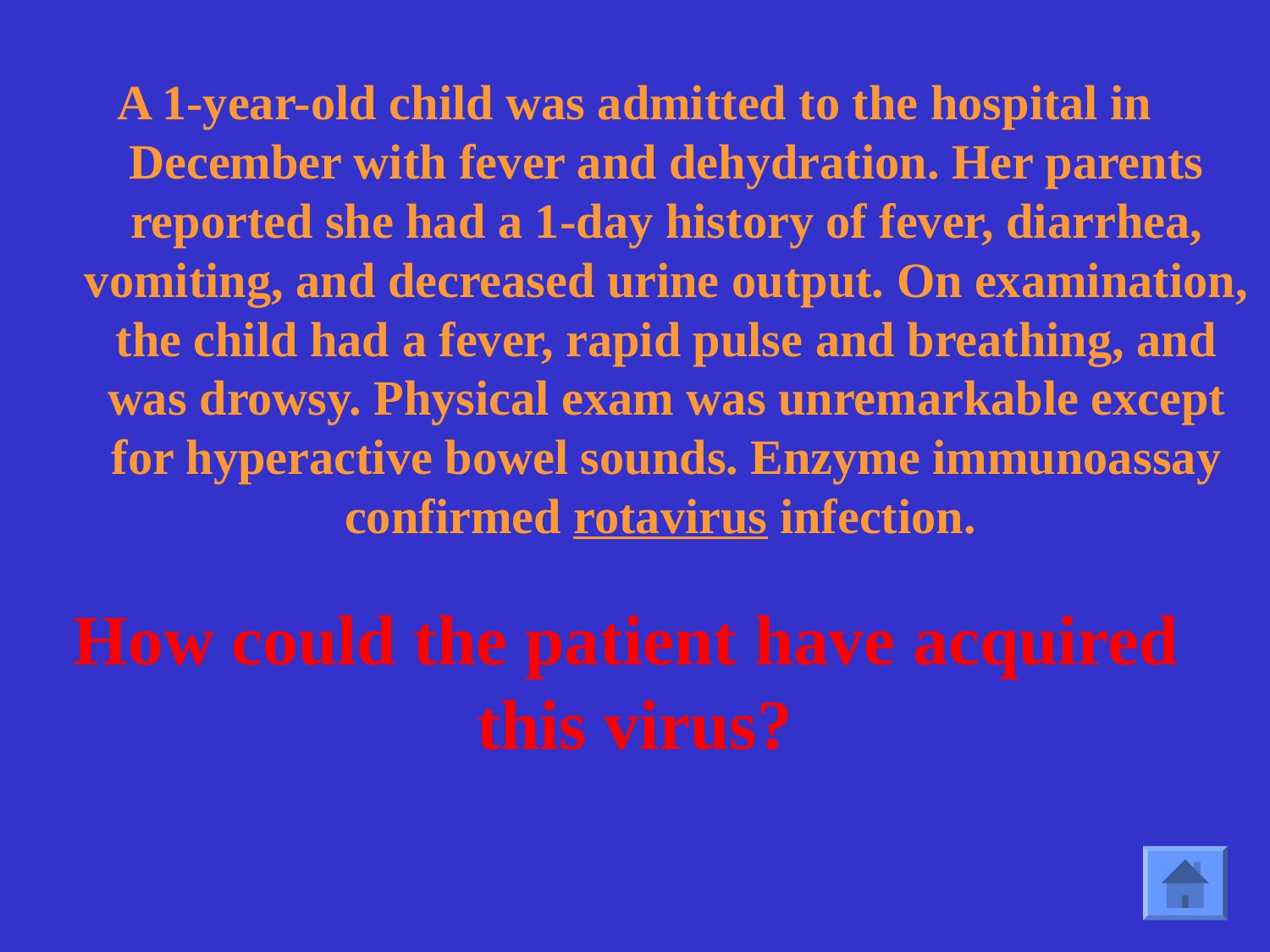

A 1-year-old child was admitted to the hospital in December with fever and dehydration. Her parents reported she had a 1-day history of fever, diarrhea, vomiting, and decreased urine output. On examination, the child had a fever, rapid pulse and breathing, and was drowsy. Physical exam was unremarkable except for hyperactive bowel sounds. Enzyme immunoassay confirmed rotavirus infection.
How could the patient have acquired
this virus?

## Slide 26
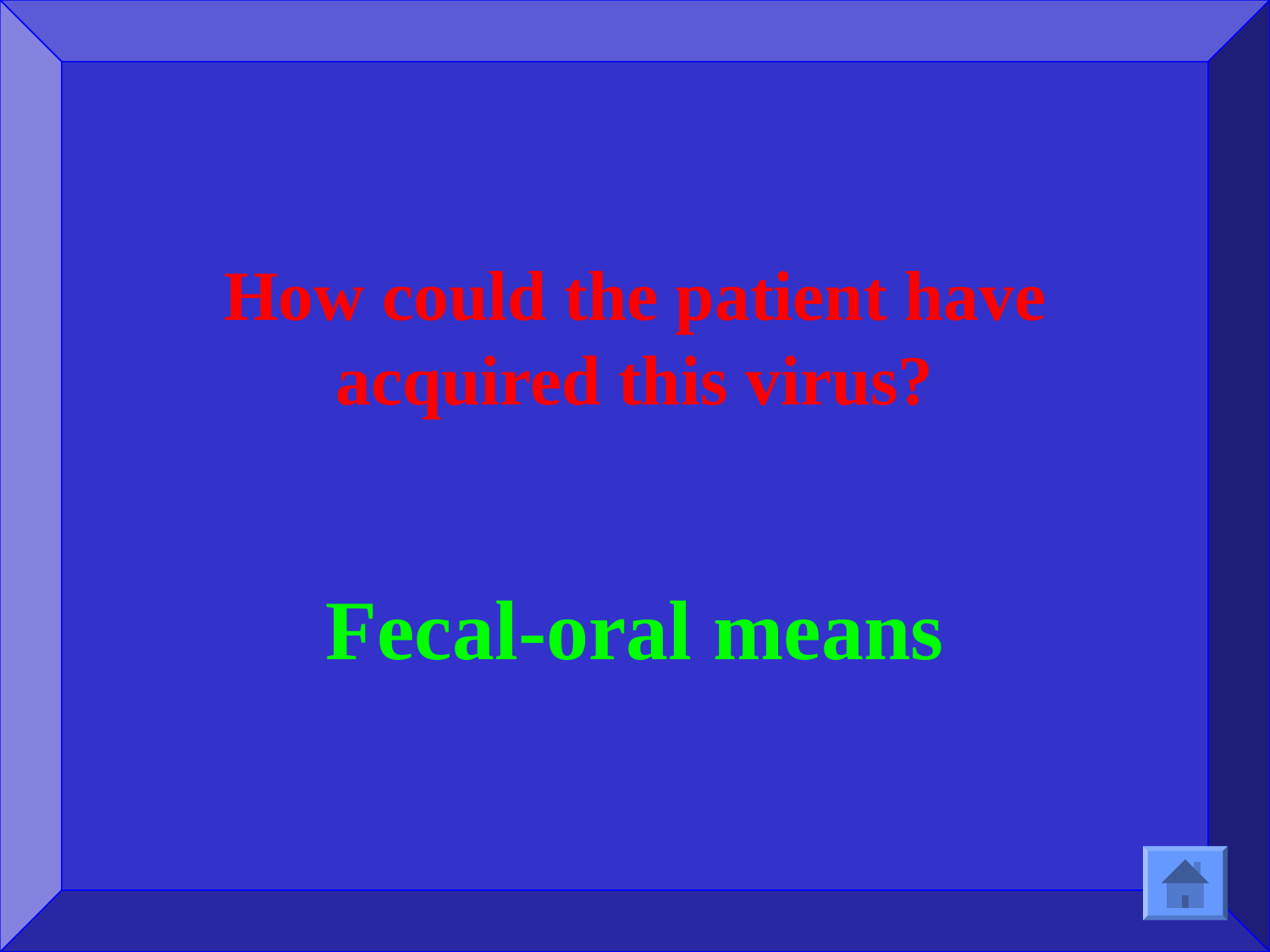

How could the patient have acquired this virus?
Fecal-oral means

## Slide 27
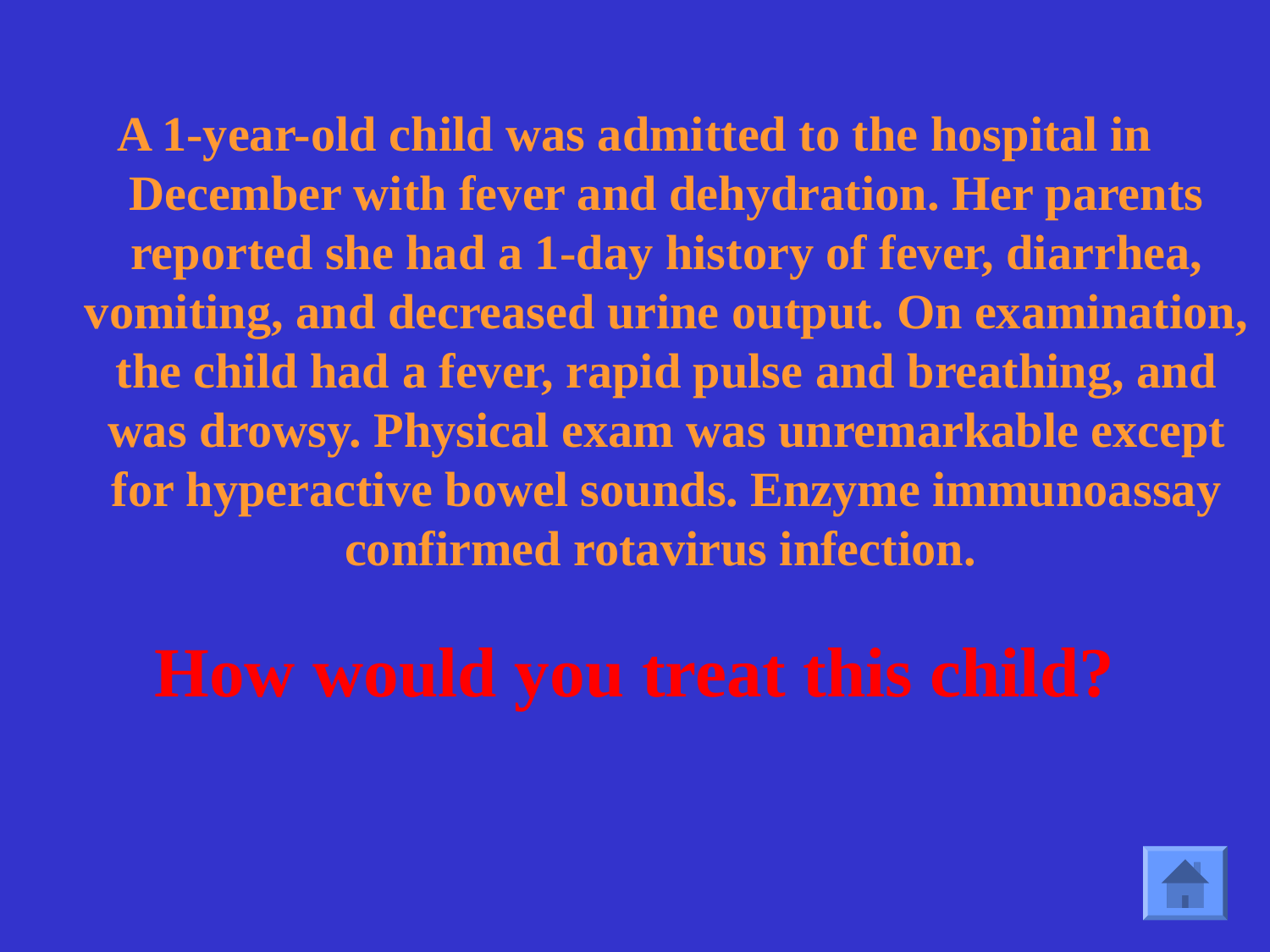

A 1-year-old child was admitted to the hospital in December with fever and dehydration. Her parents reported she had a 1-day history of fever, diarrhea, vomiting, and decreased urine output. On examination, the child had a fever, rapid pulse and breathing, and was drowsy. Physical exam was unremarkable except for hyperactive bowel sounds. Enzyme immunoassay confirmed rotavirus infection.
How would you treat this child?

## Slide 28
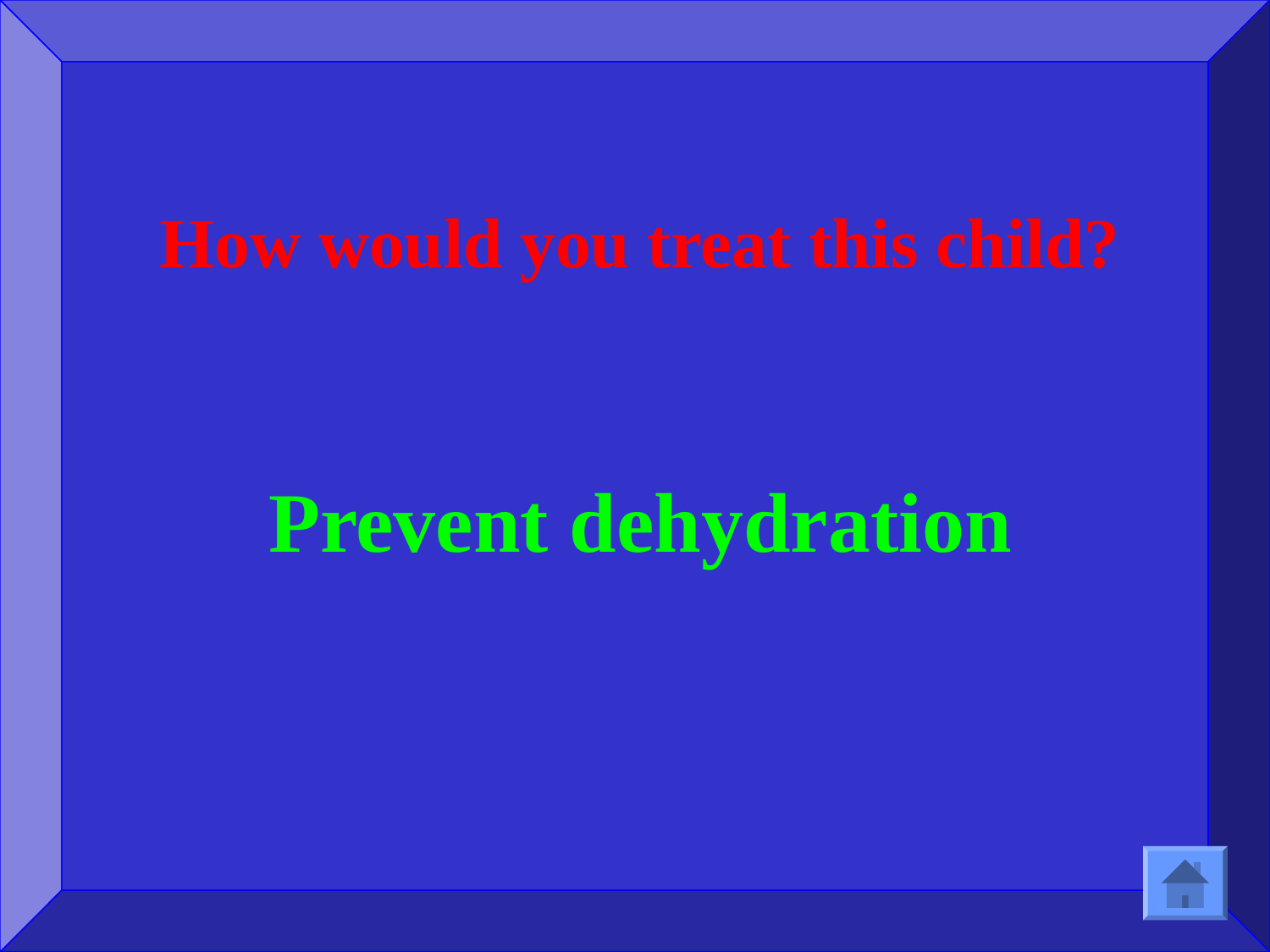

How would you treat this child?
Prevent dehydration

## Slide 29
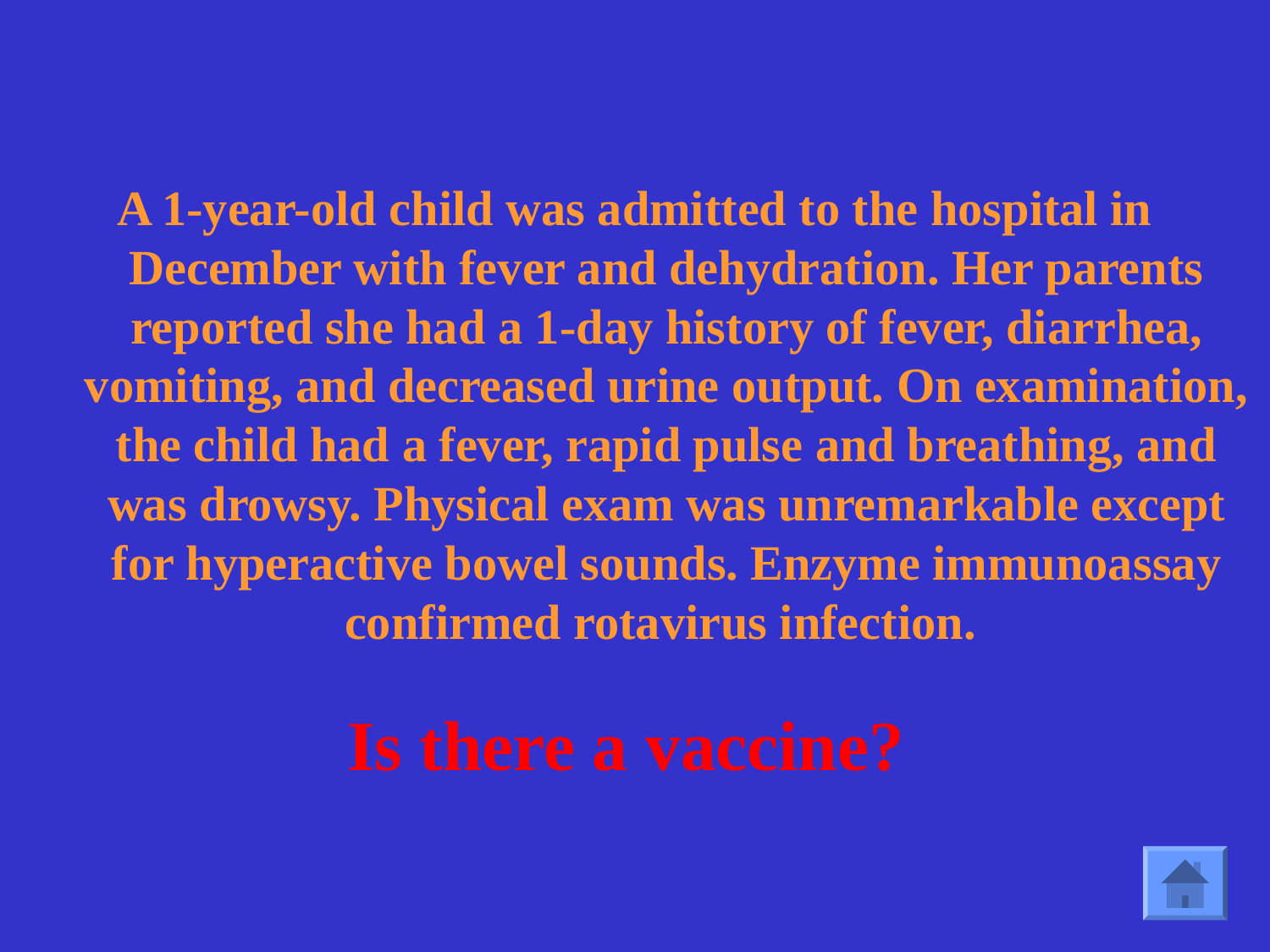

A 1-year-old child was admitted to the hospital in December with fever and dehydration. Her parents reported she had a 1-day history of fever, diarrhea, vomiting, and decreased urine output. On examination, the child had a fever, rapid pulse and breathing, and was drowsy. Physical exam was unremarkable except for hyperactive bowel sounds. Enzyme immunoassay confirmed rotavirus infection.
Is there a vaccine?

## Slide 30
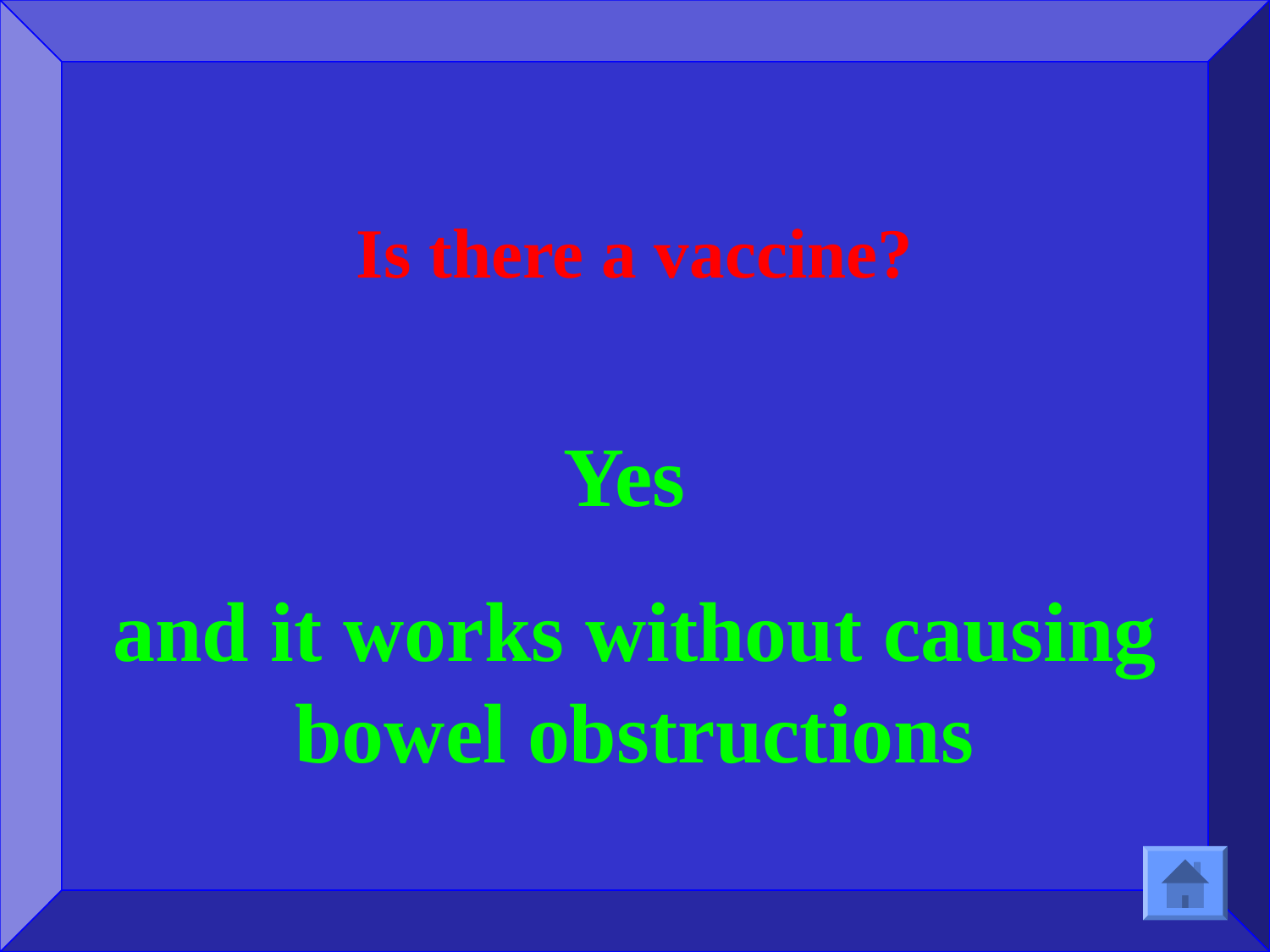

Is there a vaccine?
Yes
and it works without causing bowel obstructions

## Slide 31
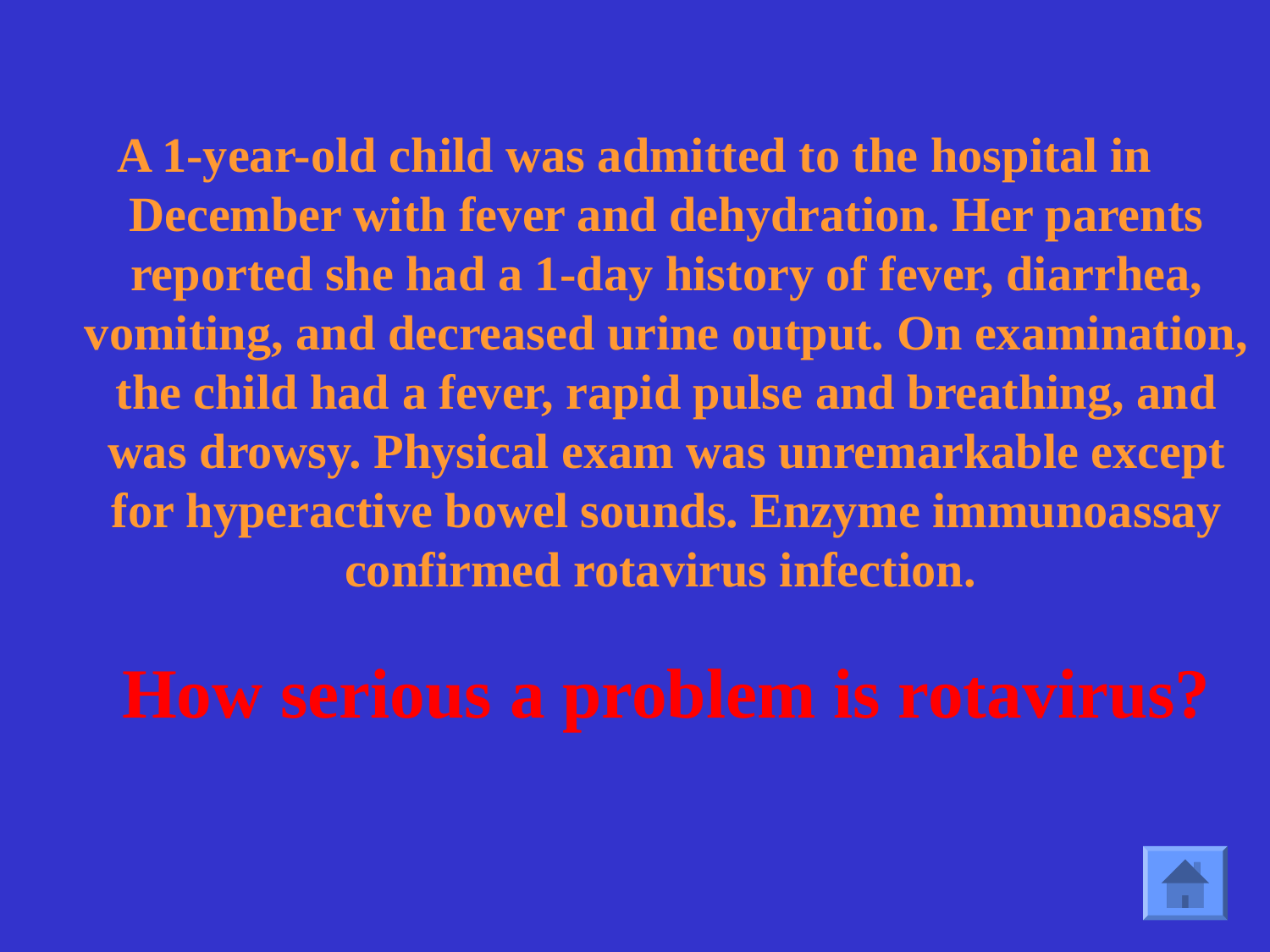

A 1-year-old child was admitted to the hospital in December with fever and dehydration. Her parents reported she had a 1-day history of fever, diarrhea, vomiting, and decreased urine output. On examination, the child had a fever, rapid pulse and breathing, and was drowsy. Physical exam was unremarkable except for hyperactive bowel sounds. Enzyme immunoassay confirmed rotavirus infection.
	How serious a problem is rotavirus?

## Slide 32
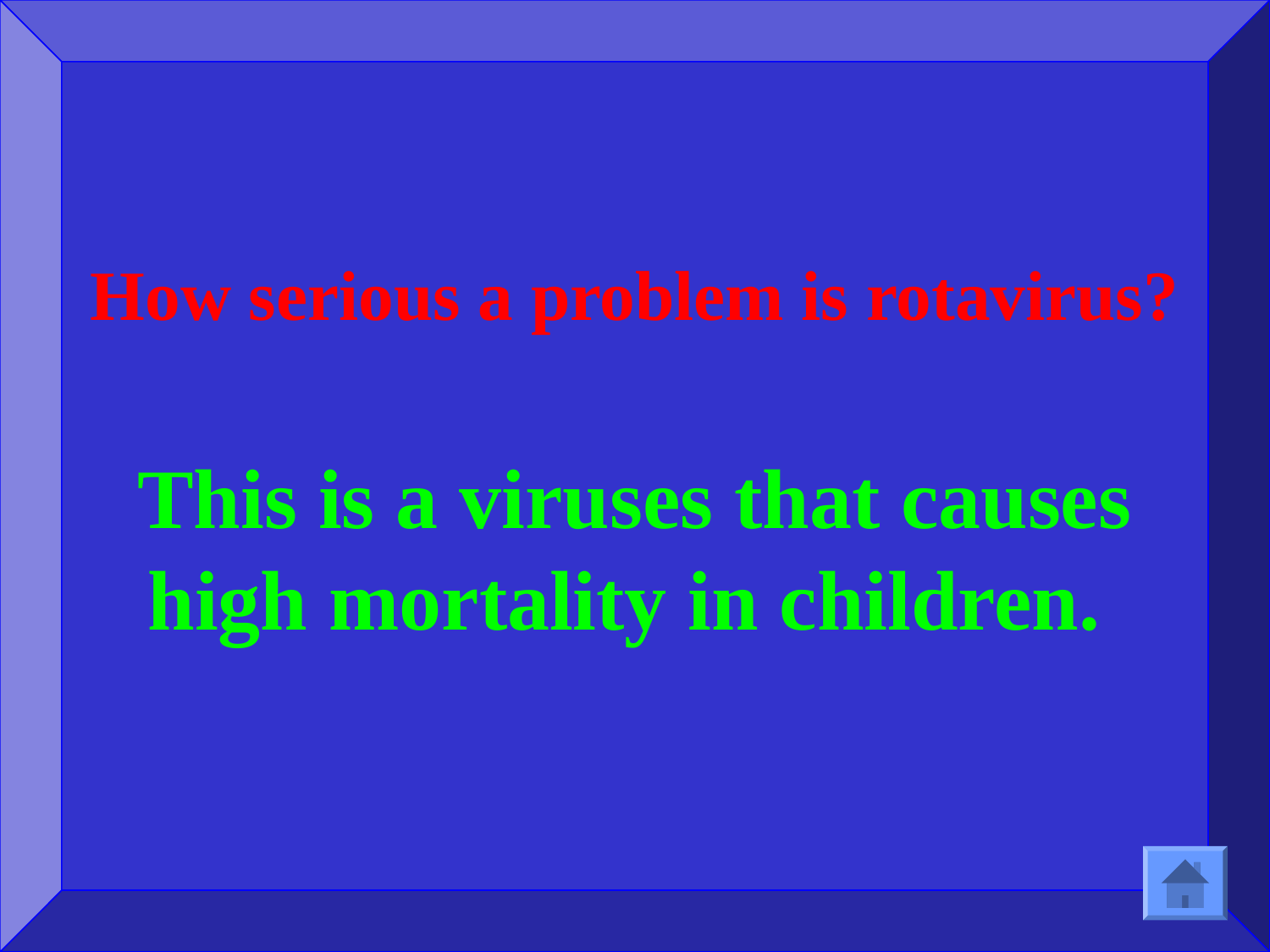

How serious a problem is rotavirus?
This is a viruses that causes high mortality in children.

## Slide 33
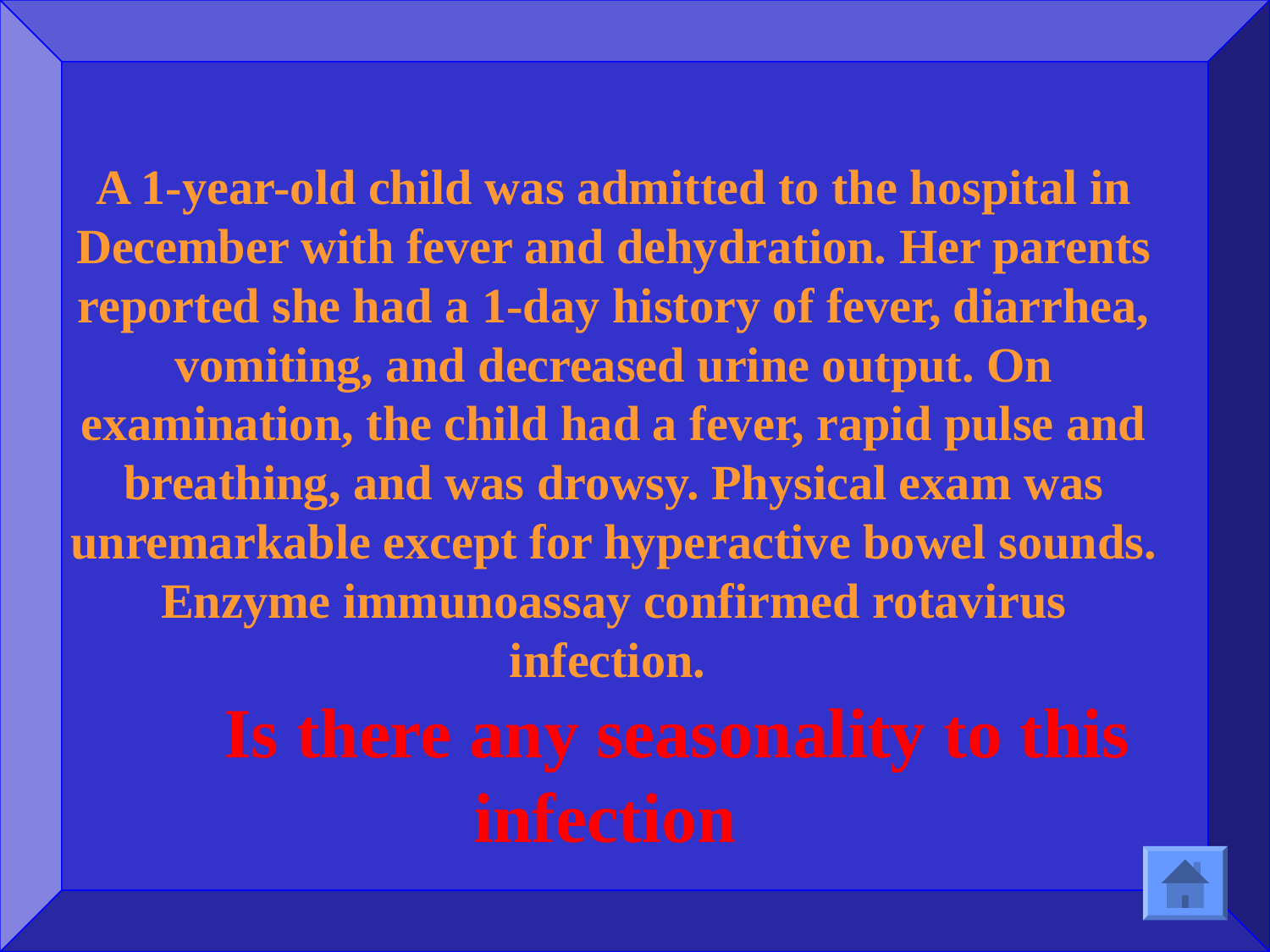

A 1-year-old child was admitted to the hospital in December with fever and dehydration. Her parents reported she had a 1-day history of fever, diarrhea, vomiting, and decreased urine output. On examination, the child had a fever, rapid pulse and breathing, and was drowsy. Physical exam was unremarkable except for hyperactive bowel sounds. Enzyme immunoassay confirmed rotavirus infection.
	Is there any seasonality to this infection

## Slide 34
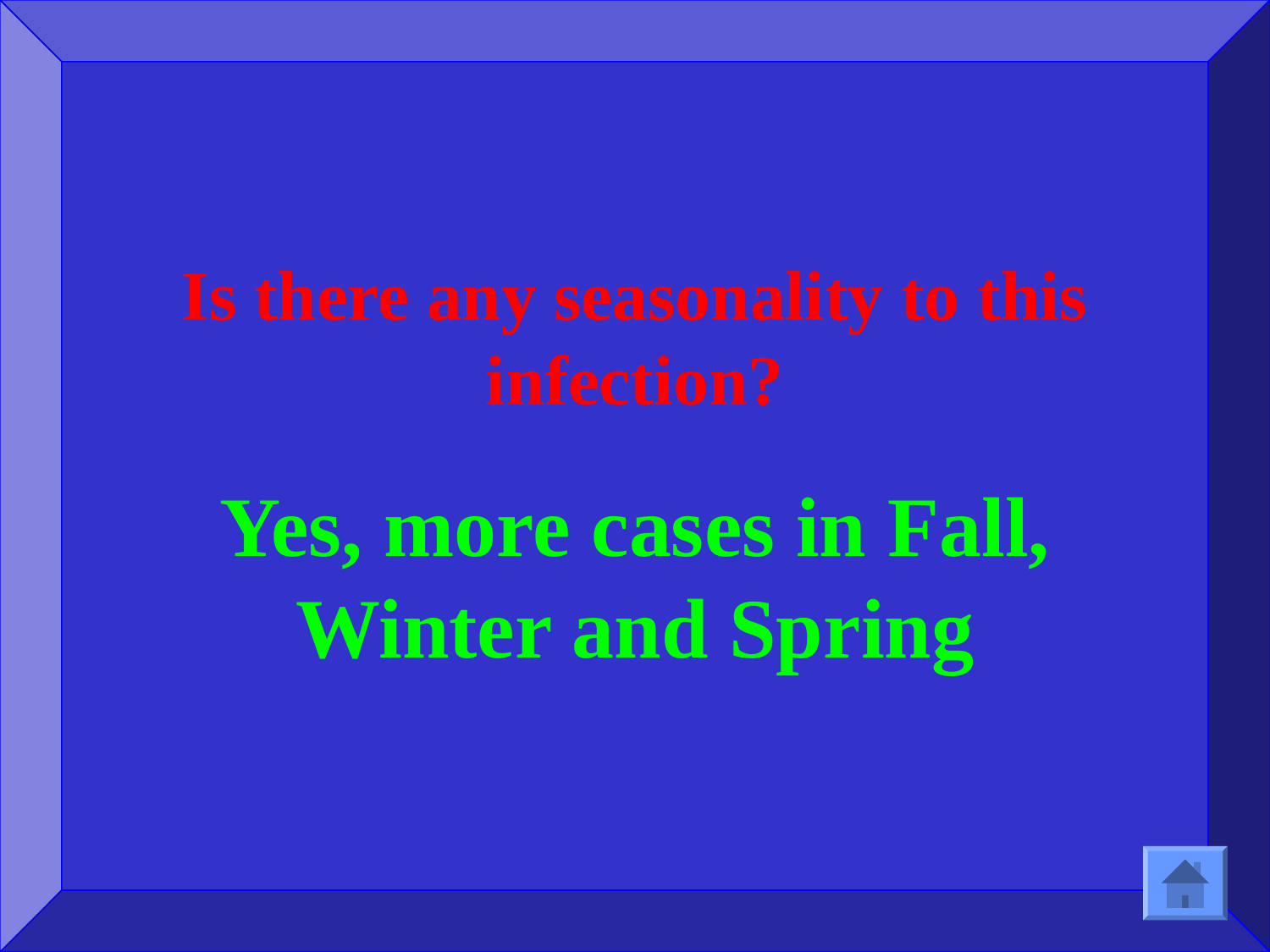

Is there any seasonality to this infection?
Yes, more cases in Fall, Winter and Spring

## Slide 35
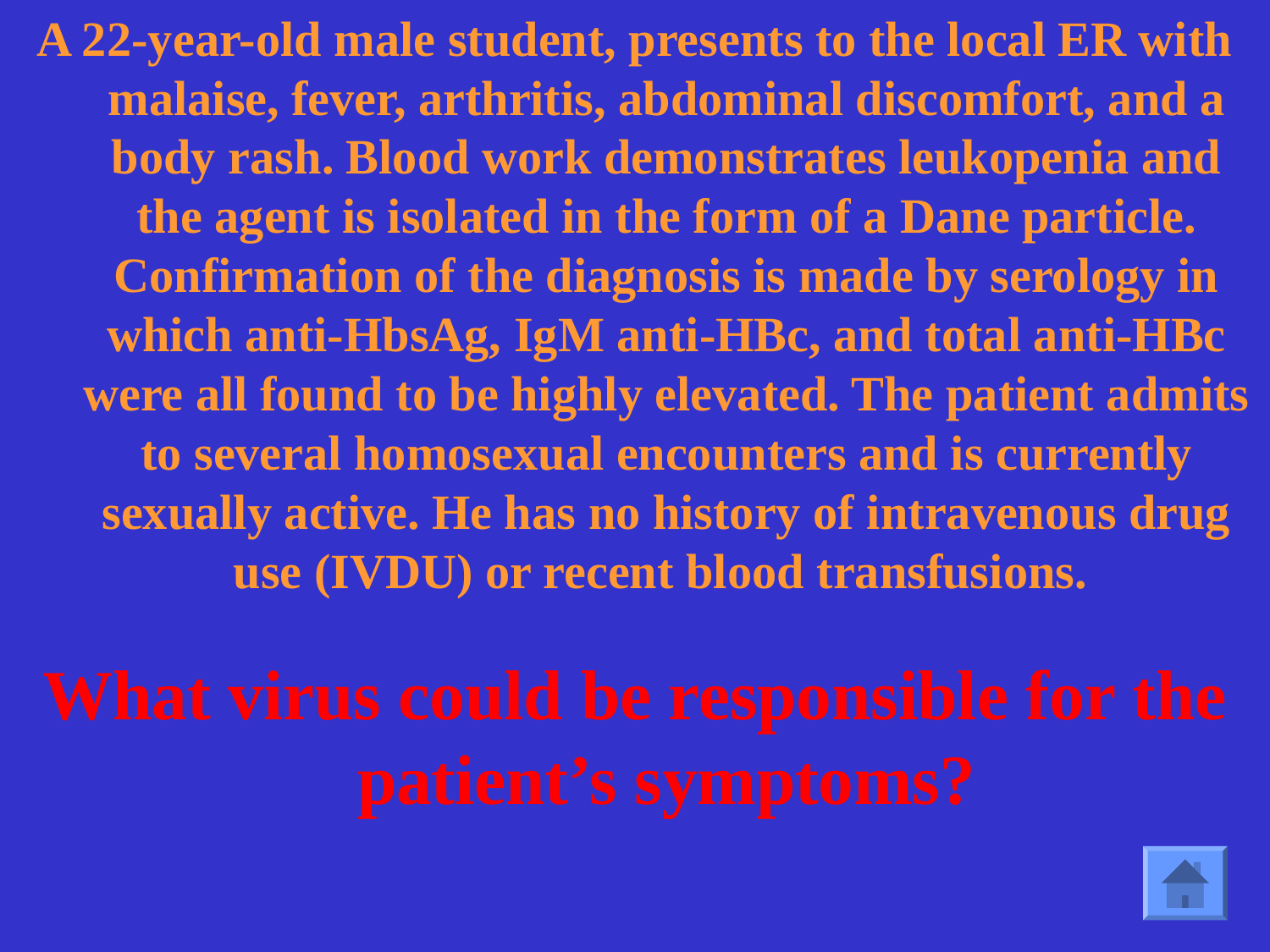

A 22-year-old male student, presents to the local ER with malaise, fever, arthritis, abdominal discomfort, and a body rash. Blood work demonstrates leukopenia and the agent is isolated in the form of a Dane particle. Confirmation of the diagnosis is made by serology in which anti-HbsAg, IgM anti-HBc, and total anti-HBc were all found to be highly elevated. The patient admits to several homosexual encounters and is currently sexually active. He has no history of intravenous drug use (IVDU) or recent blood transfusions.
What virus could be responsible for the patient’s symptoms?

## Slide 36
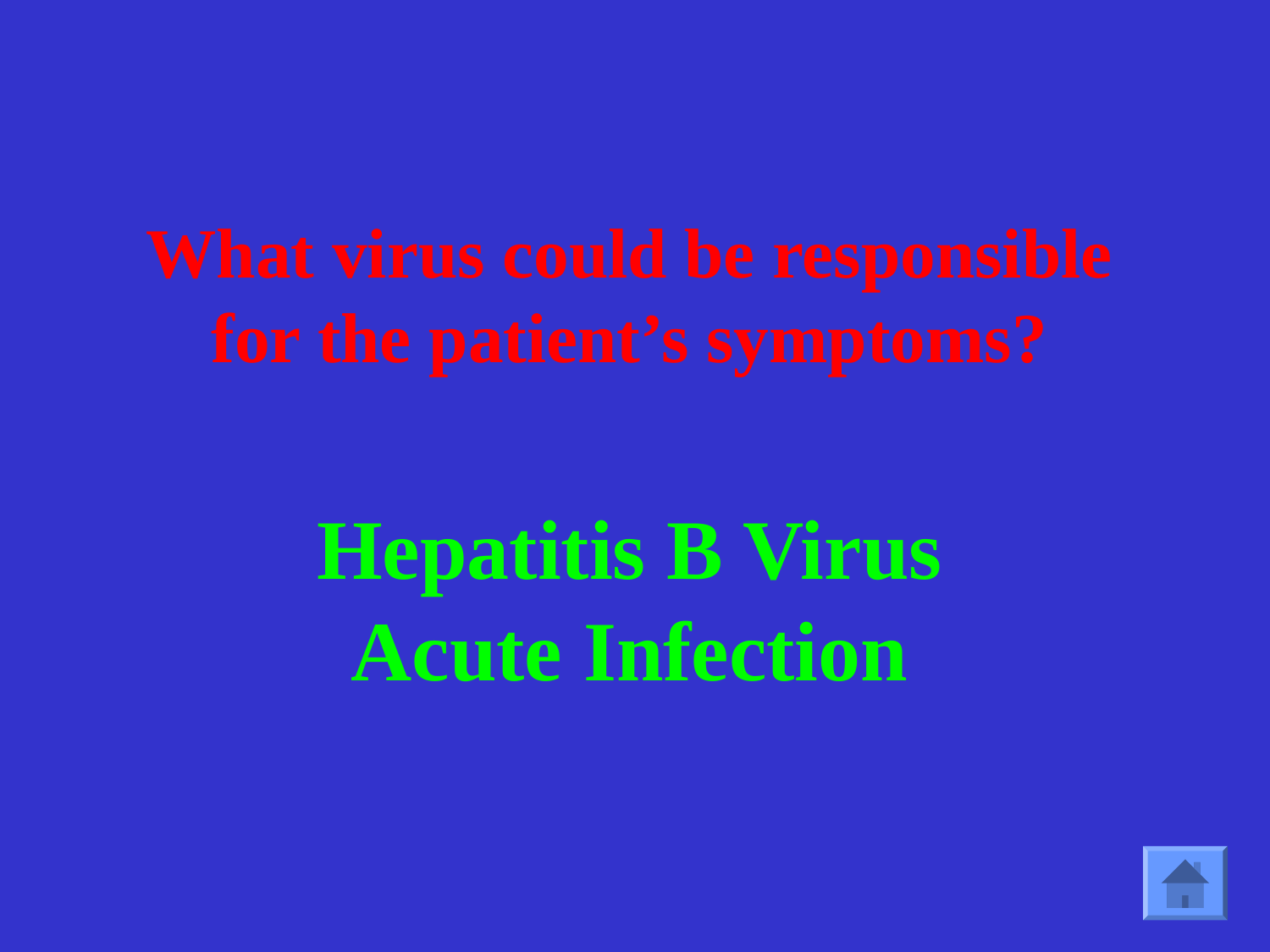

What virus could be responsible for the patient’s symptoms?
Hepatitis B Virus
Acute Infection

## Slide 37
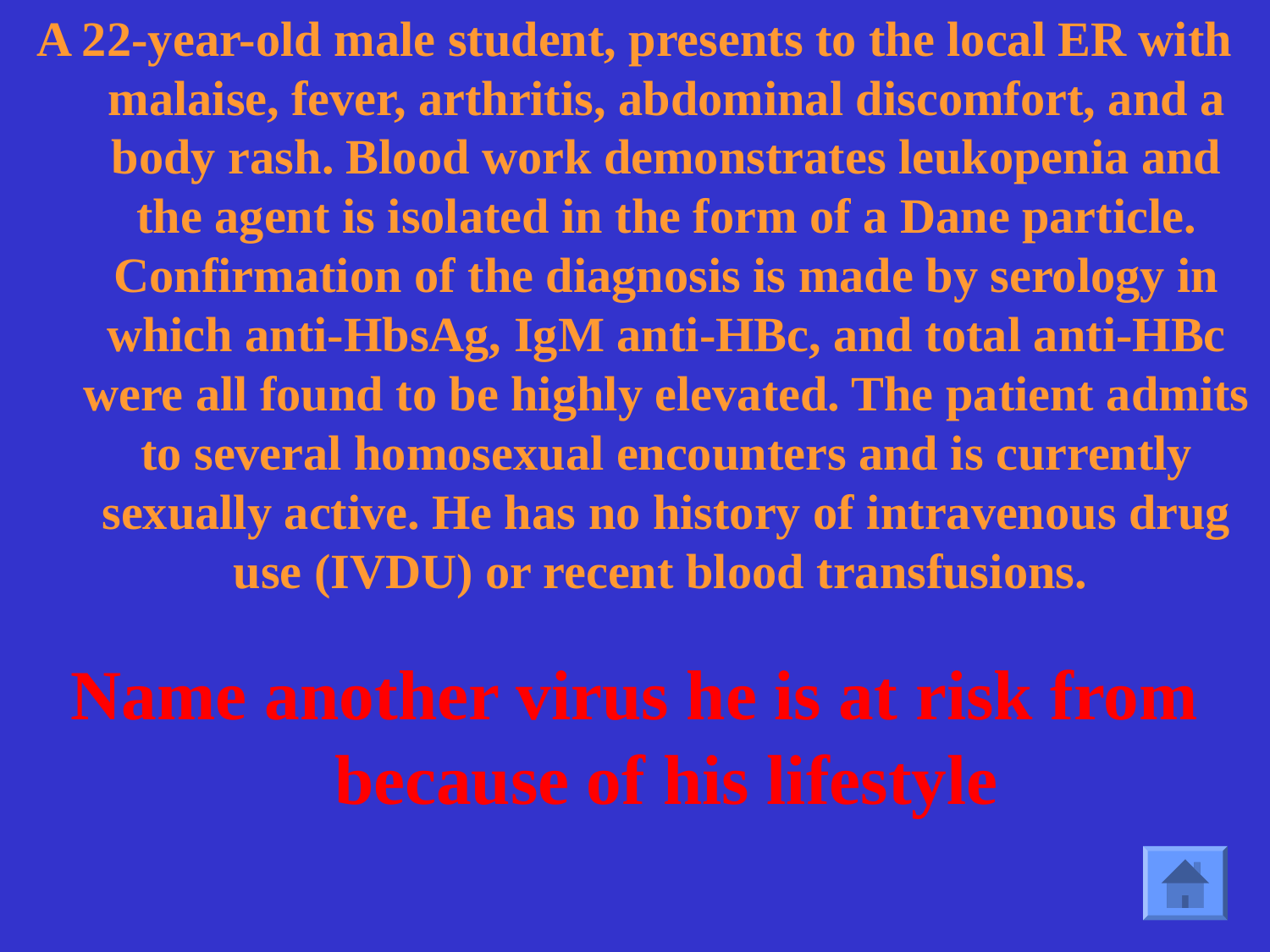

A 22-year-old male student, presents to the local ER with malaise, fever, arthritis, abdominal discomfort, and a body rash. Blood work demonstrates leukopenia and the agent is isolated in the form of a Dane particle. Confirmation of the diagnosis is made by serology in which anti-HbsAg, IgM anti-HBc, and total anti-HBc were all found to be highly elevated. The patient admits to several homosexual encounters and is currently sexually active. He has no history of intravenous drug use (IVDU) or recent blood transfusions.
Name another virus he is at risk from because of his lifestyle

## Slide 38
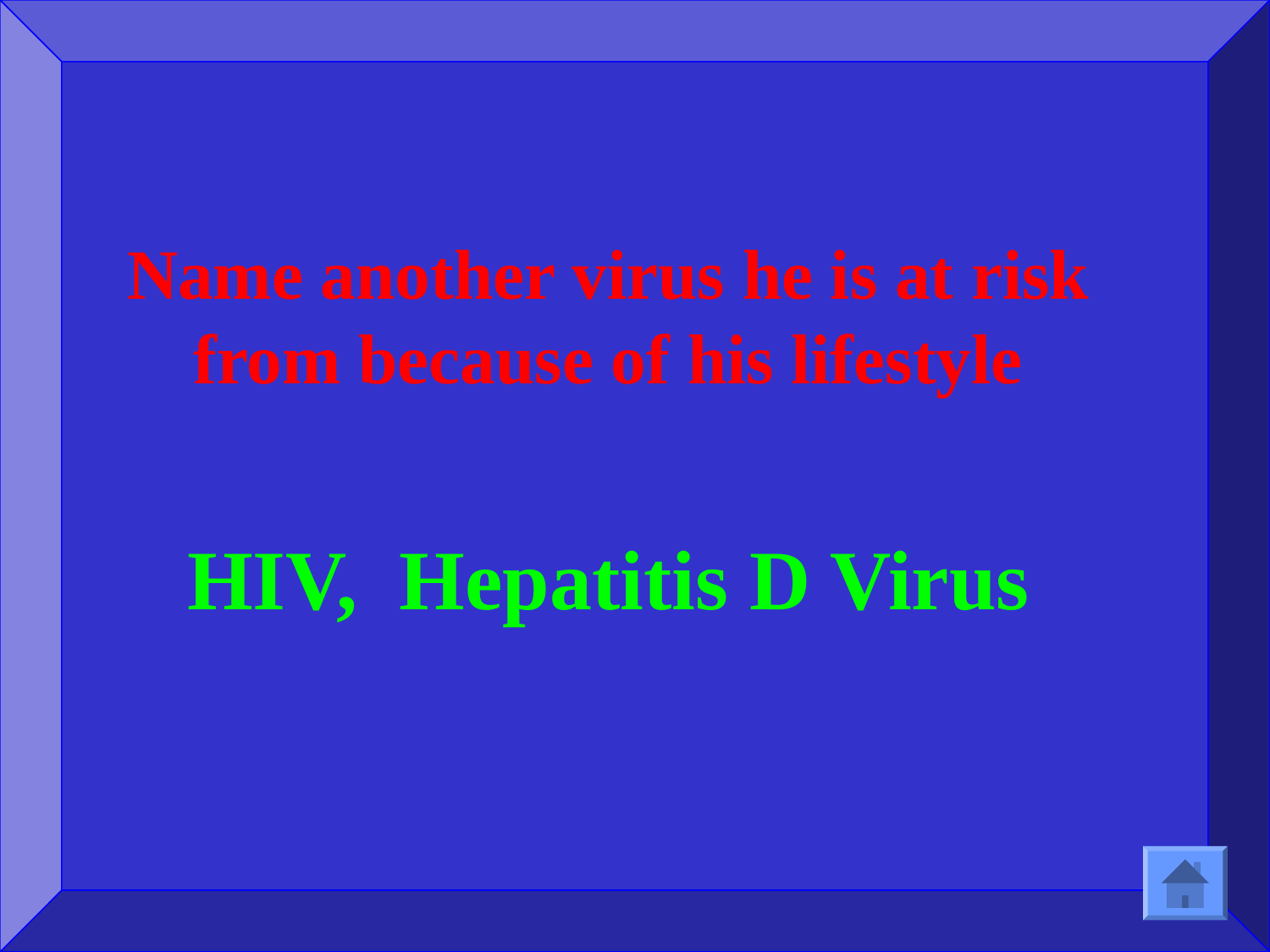

Name another virus he is at risk from because of his lifestyle
HIV, Hepatitis D Virus

## Slide 39
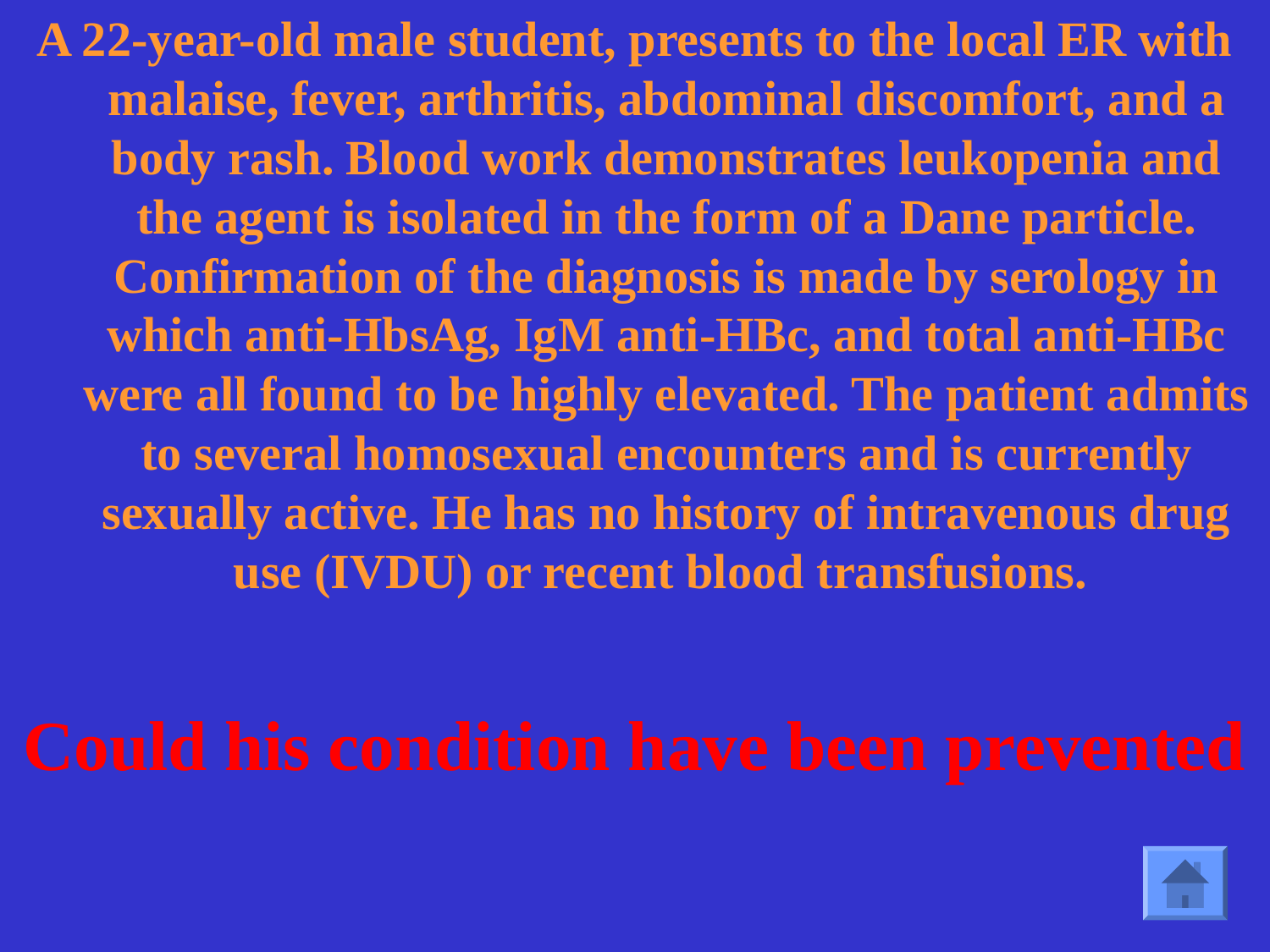

A 22-year-old male student, presents to the local ER with malaise, fever, arthritis, abdominal discomfort, and a body rash. Blood work demonstrates leukopenia and the agent is isolated in the form of a Dane particle. Confirmation of the diagnosis is made by serology in which anti-HbsAg, IgM anti-HBc, and total anti-HBc were all found to be highly elevated. The patient admits to several homosexual encounters and is currently sexually active. He has no history of intravenous drug use (IVDU) or recent blood transfusions.
Could his condition have been prevented

## Slide 40
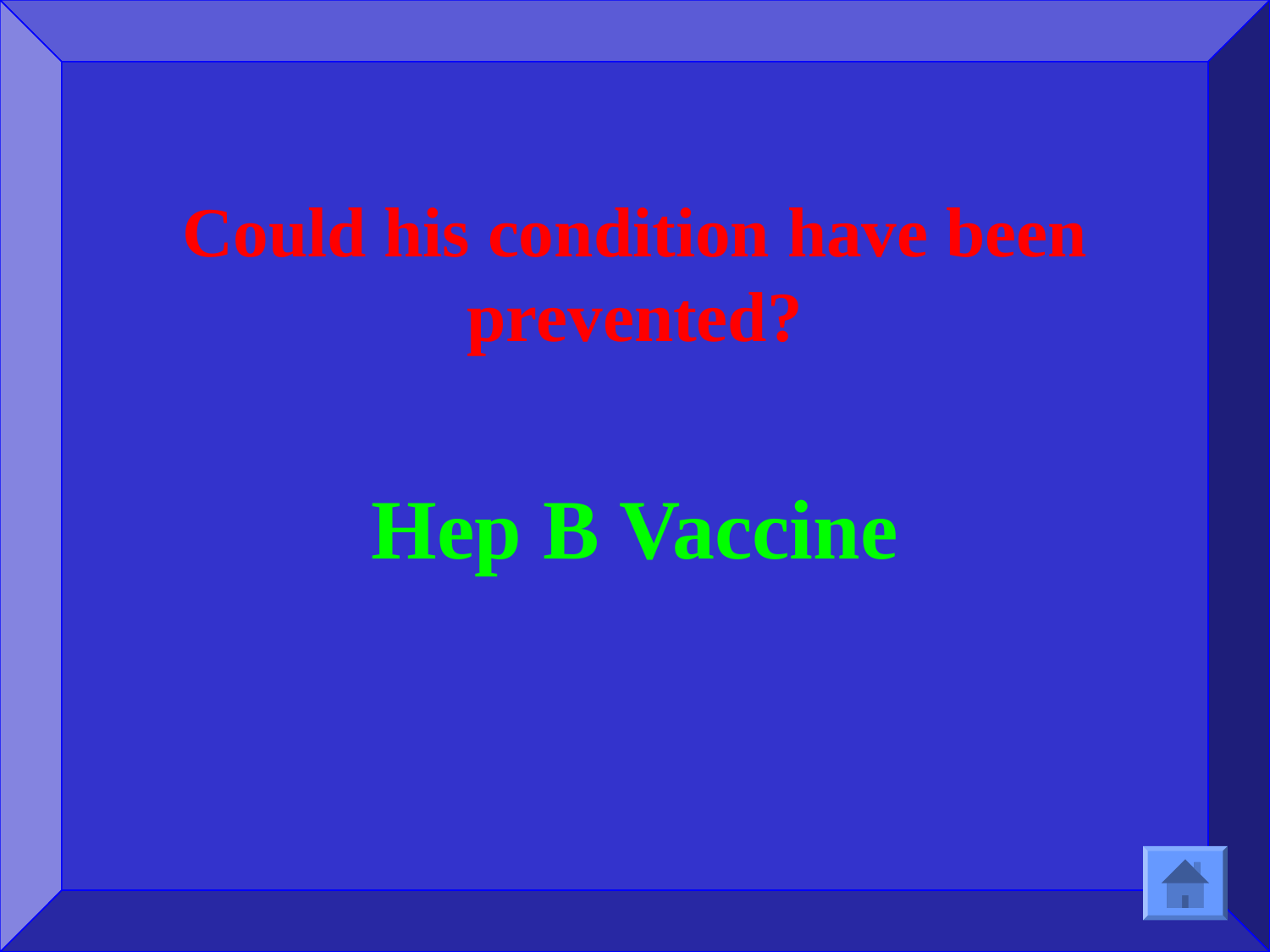

Could his condition have been prevented?
Hep B Vaccine

## Slide 41
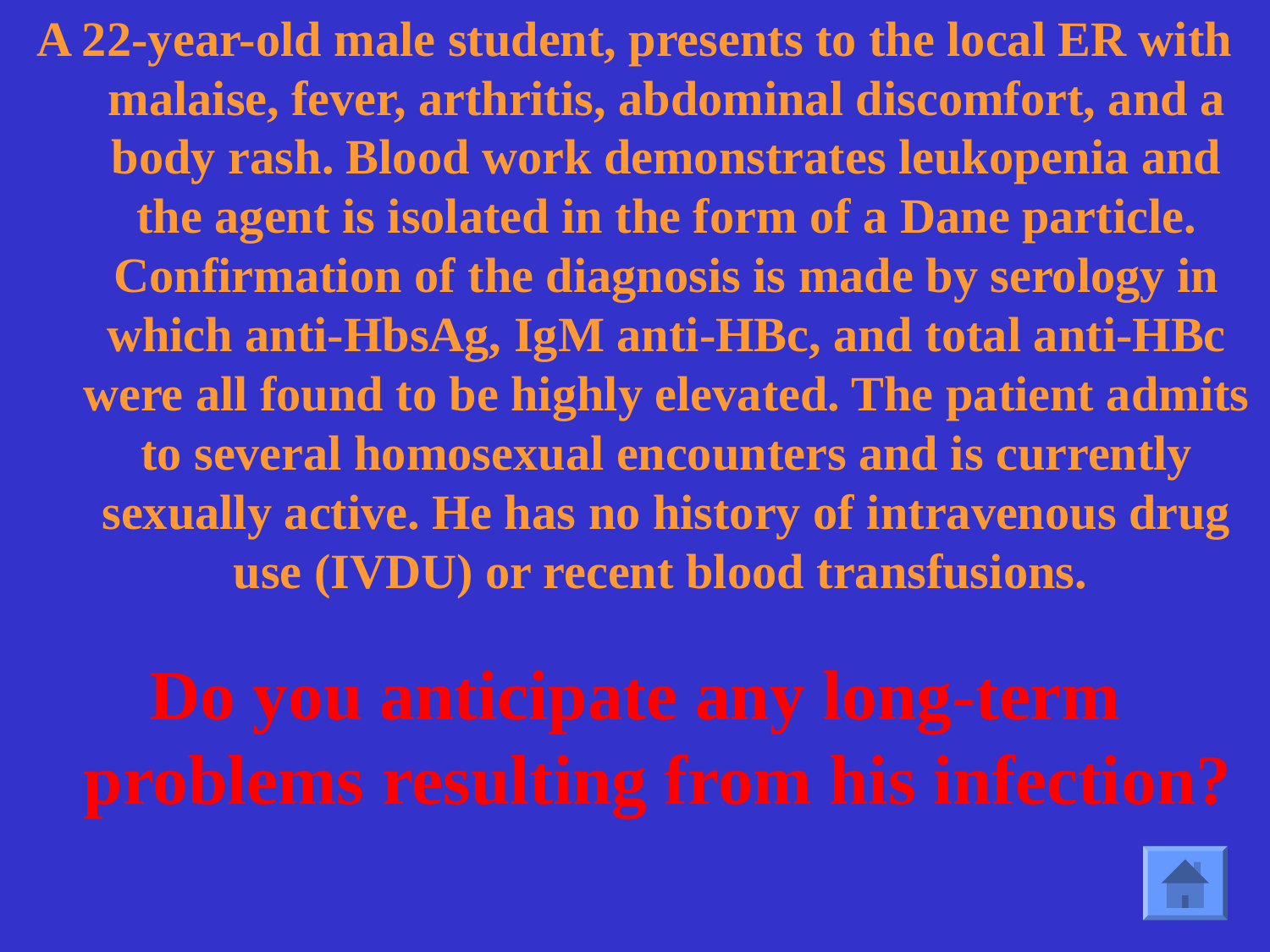

A 22-year-old male student, presents to the local ER with malaise, fever, arthritis, abdominal discomfort, and a body rash. Blood work demonstrates leukopenia and the agent is isolated in the form of a Dane particle. Confirmation of the diagnosis is made by serology in which anti-HbsAg, IgM anti-HBc, and total anti-HBc were all found to be highly elevated. The patient admits to several homosexual encounters and is currently sexually active. He has no history of intravenous drug use (IVDU) or recent blood transfusions.
Do you anticipate any long-term problems resulting from his infection?

## Slide 42
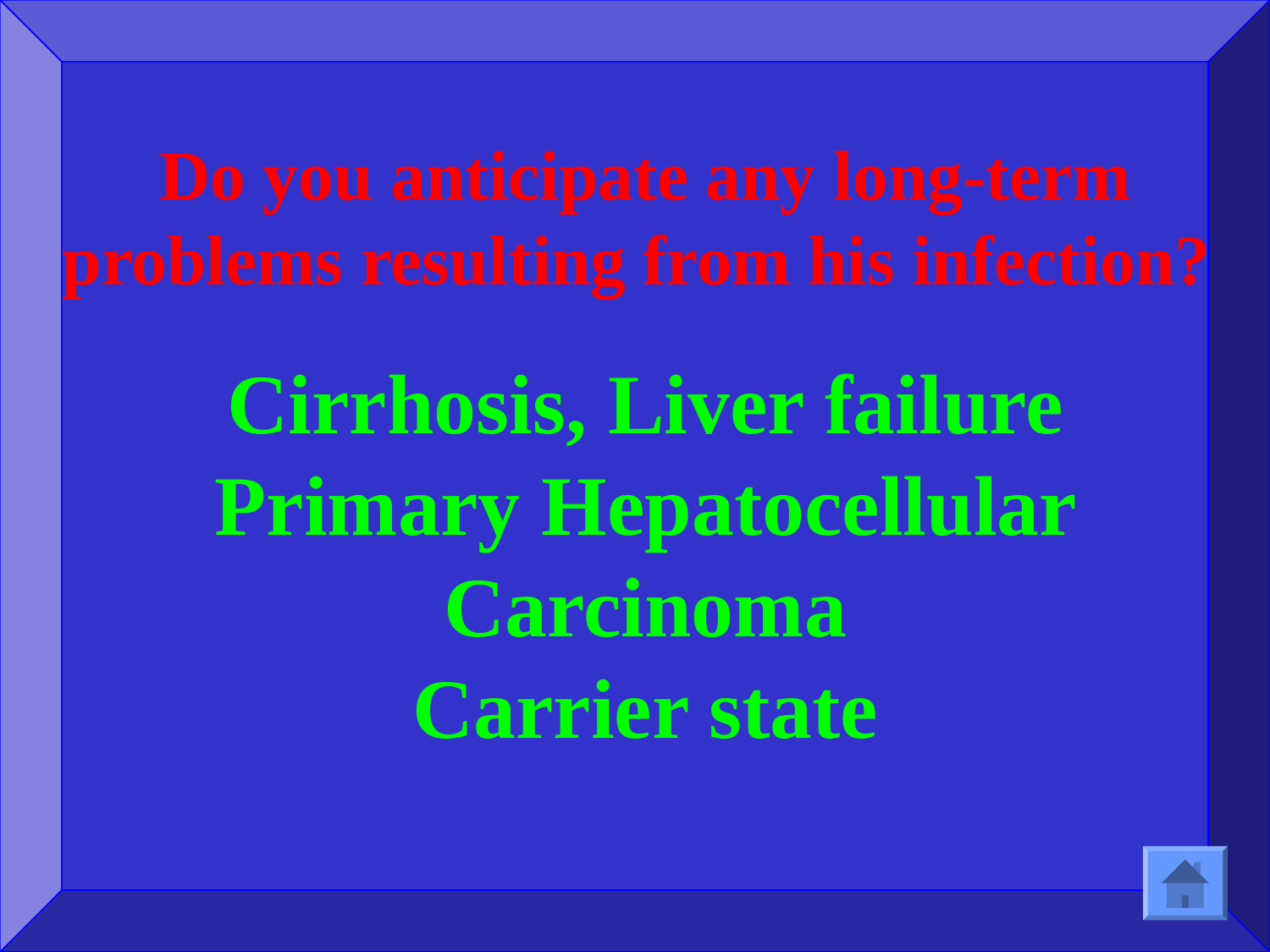

Do you anticipate any long-term problems resulting from his infection?
Cirrhosis, Liver failure
Primary Hepatocellular Carcinoma
Carrier state

## Slide 43
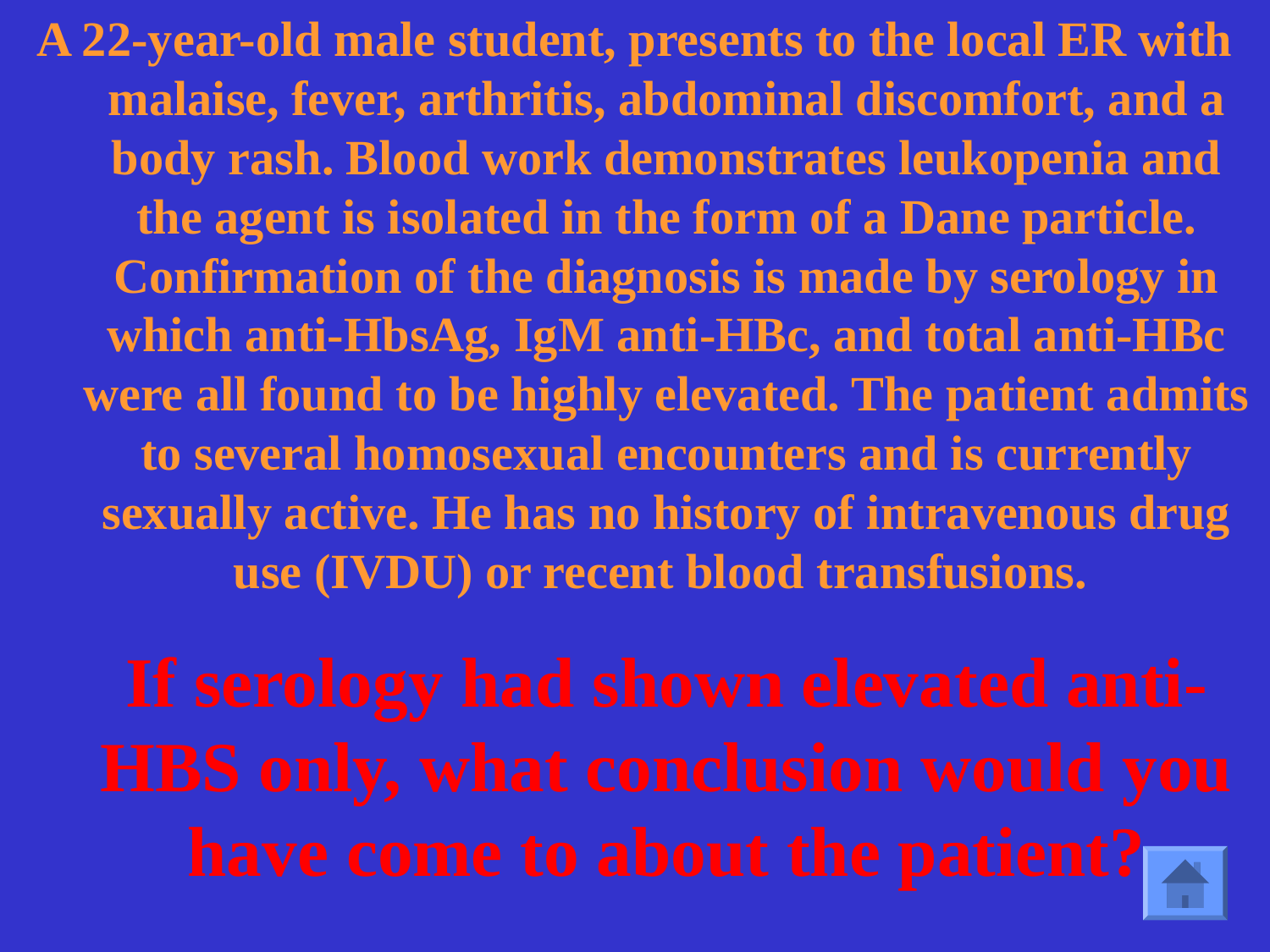

A 22-year-old male student, presents to the local ER with malaise, fever, arthritis, abdominal discomfort, and a body rash. Blood work demonstrates leukopenia and the agent is isolated in the form of a Dane particle. Confirmation of the diagnosis is made by serology in which anti-HbsAg, IgM anti-HBc, and total anti-HBc were all found to be highly elevated. The patient admits to several homosexual encounters and is currently sexually active. He has no history of intravenous drug use (IVDU) or recent blood transfusions.
	If serology had shown elevated anti-HBS only, what conclusion would you have come to about the patient?

## Slide 44
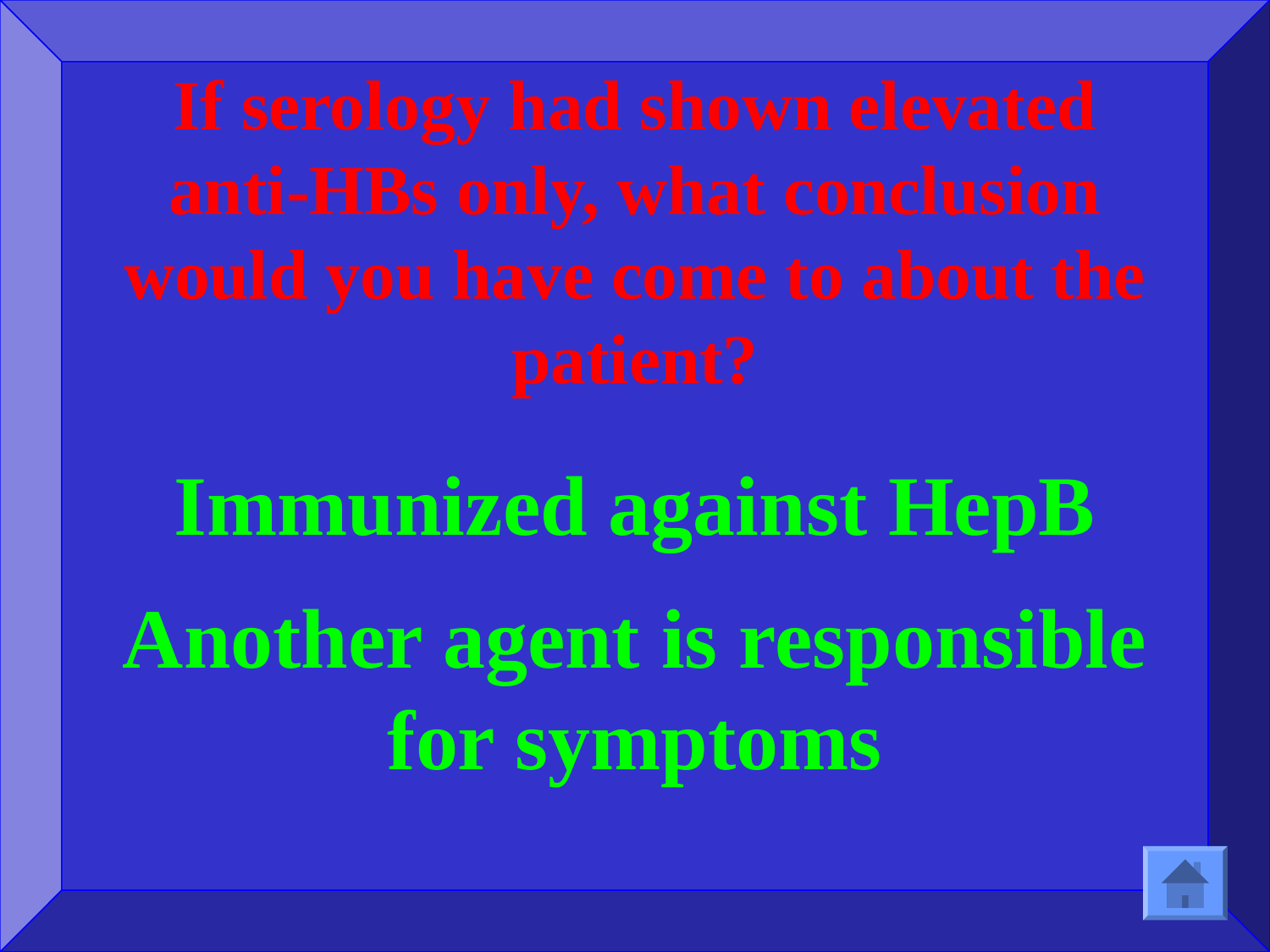

If serology had shown elevated anti-HBs only, what conclusion would you have come to about the patient?
Immunized against HepB
Another agent is responsible for symptoms

## Slide 45
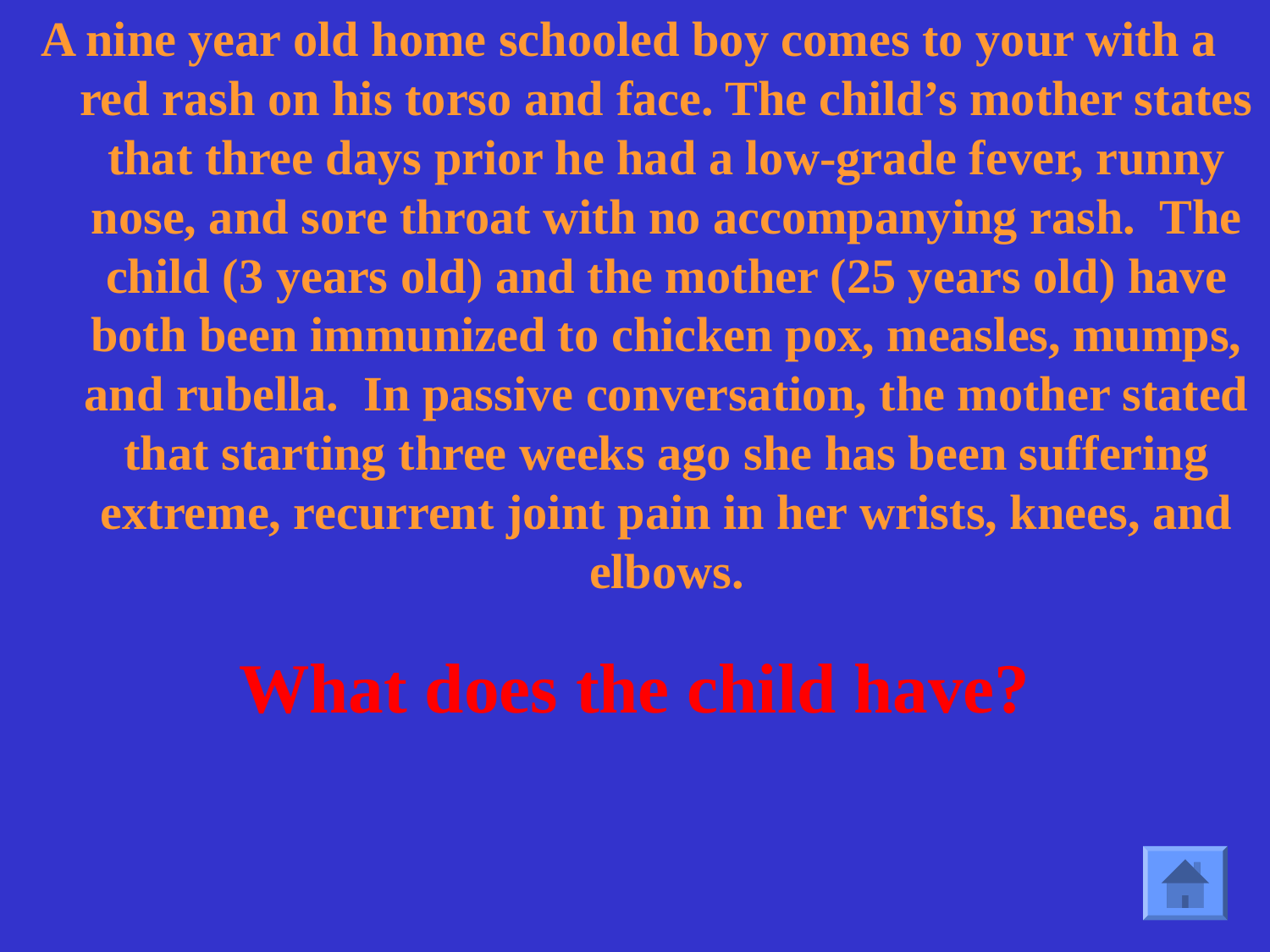

A nine year old home schooled boy comes to your with a red rash on his torso and face. The child’s mother states that three days prior he had a low-grade fever, runny nose, and sore throat with no accompanying rash. The child (3 years old) and the mother (25 years old) have both been immunized to chicken pox, measles, mumps, and rubella. In passive conversation, the mother stated that starting three weeks ago she has been suffering extreme, recurrent joint pain in her wrists, knees, and elbows.
What does the child have?

## Slide 46
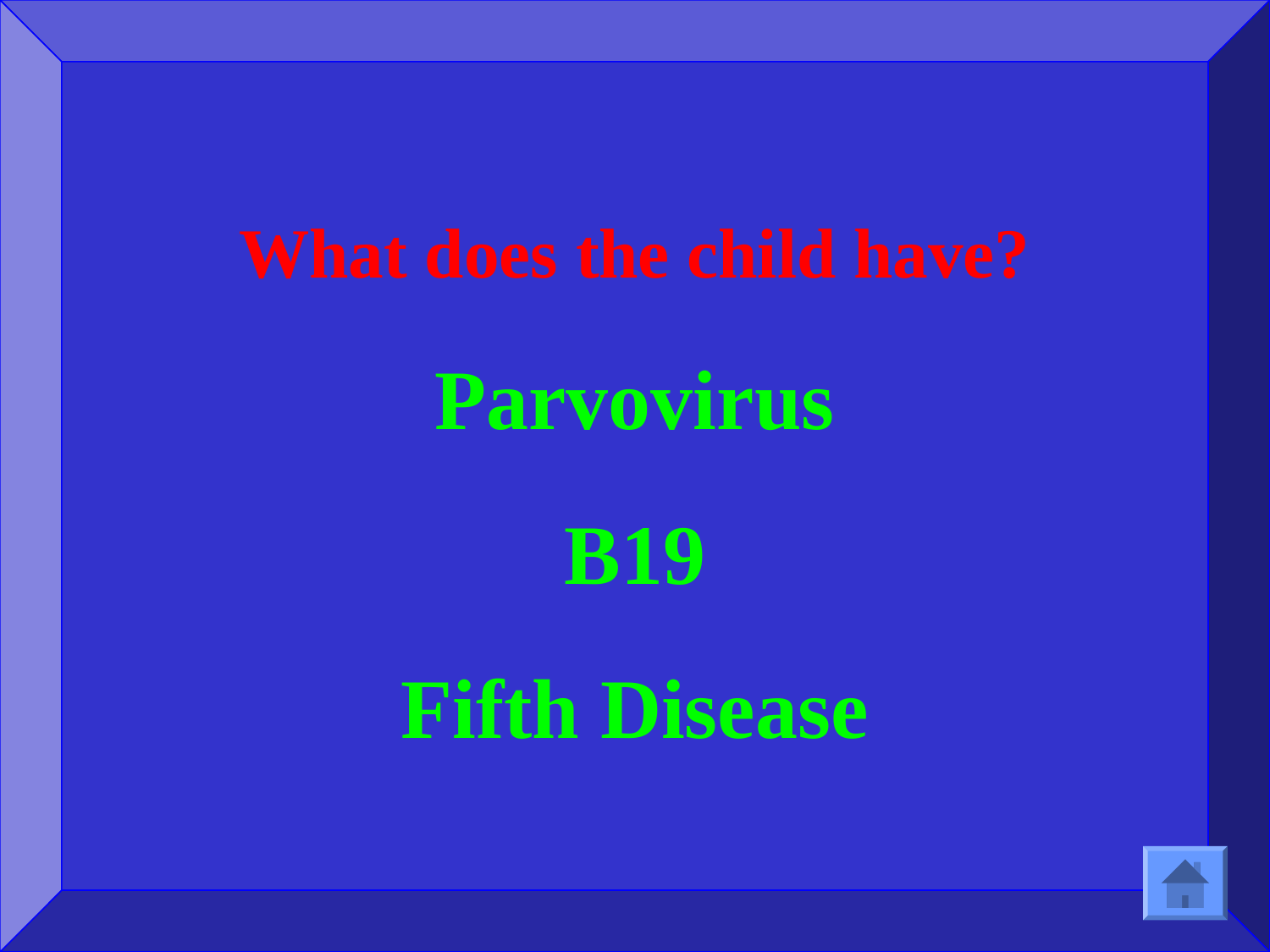

What does the child have?
Parvovirus
B19
Fifth Disease

## Slide 47
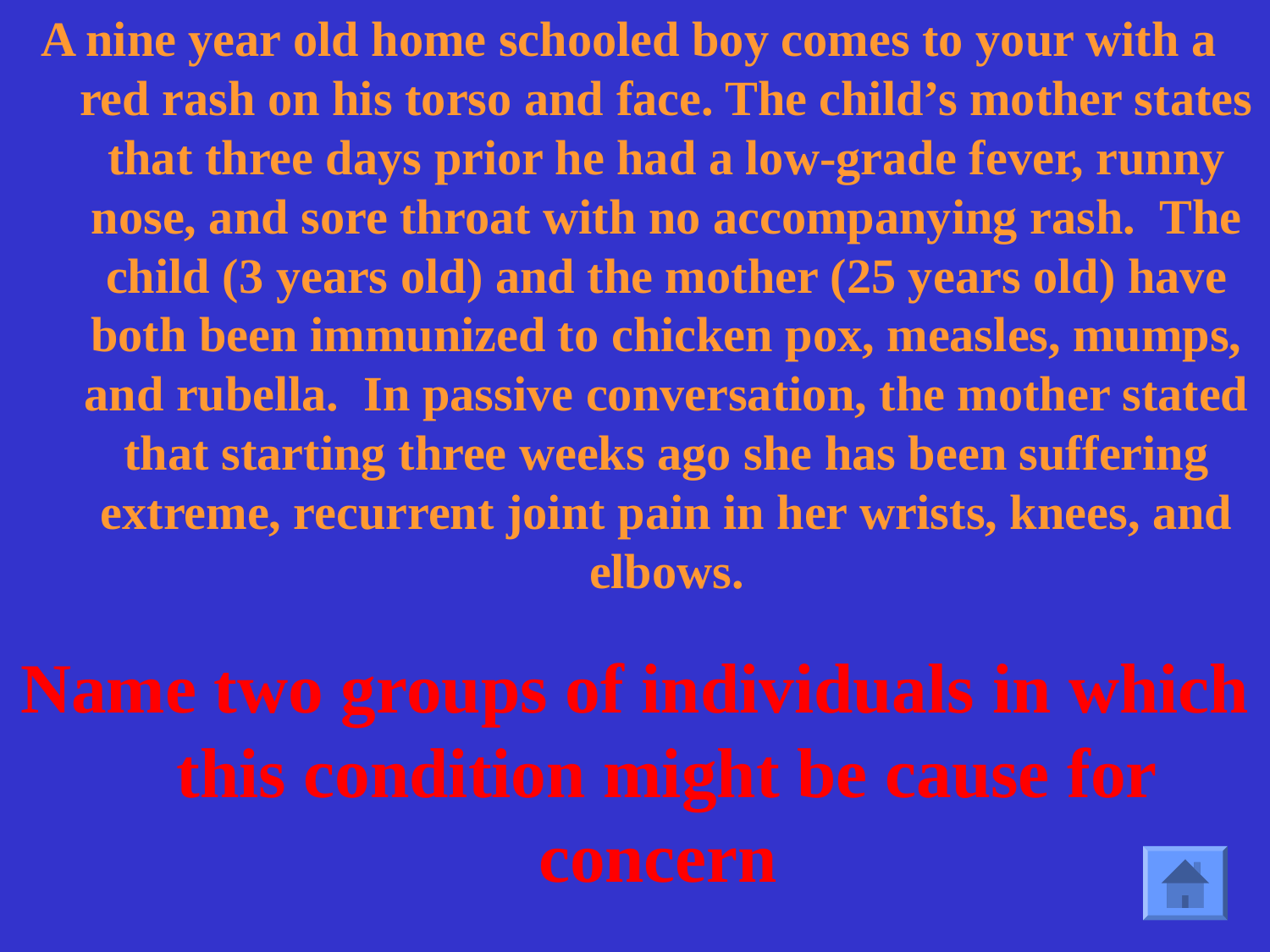

A nine year old home schooled boy comes to your with a red rash on his torso and face. The child’s mother states that three days prior he had a low-grade fever, runny nose, and sore throat with no accompanying rash. The child (3 years old) and the mother (25 years old) have both been immunized to chicken pox, measles, mumps, and rubella. In passive conversation, the mother stated that starting three weeks ago she has been suffering extreme, recurrent joint pain in her wrists, knees, and elbows.
Name two groups of individuals in which this condition might be cause for concern

## Slide 48
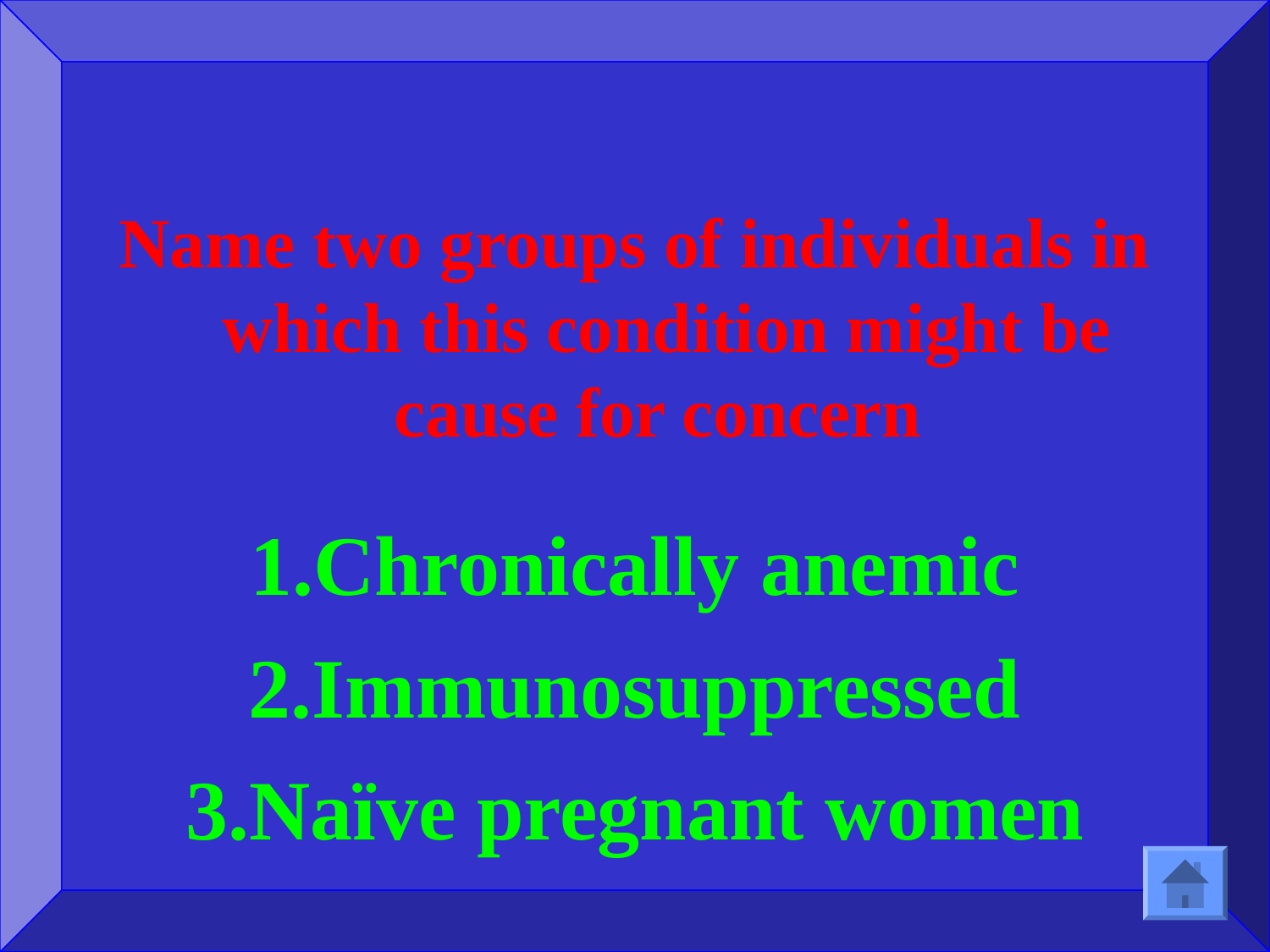

Name two groups of individuals in which this condition might be cause for concern
Chronically anemic
Immunosuppressed
Naïve pregnant women

## Slide 49
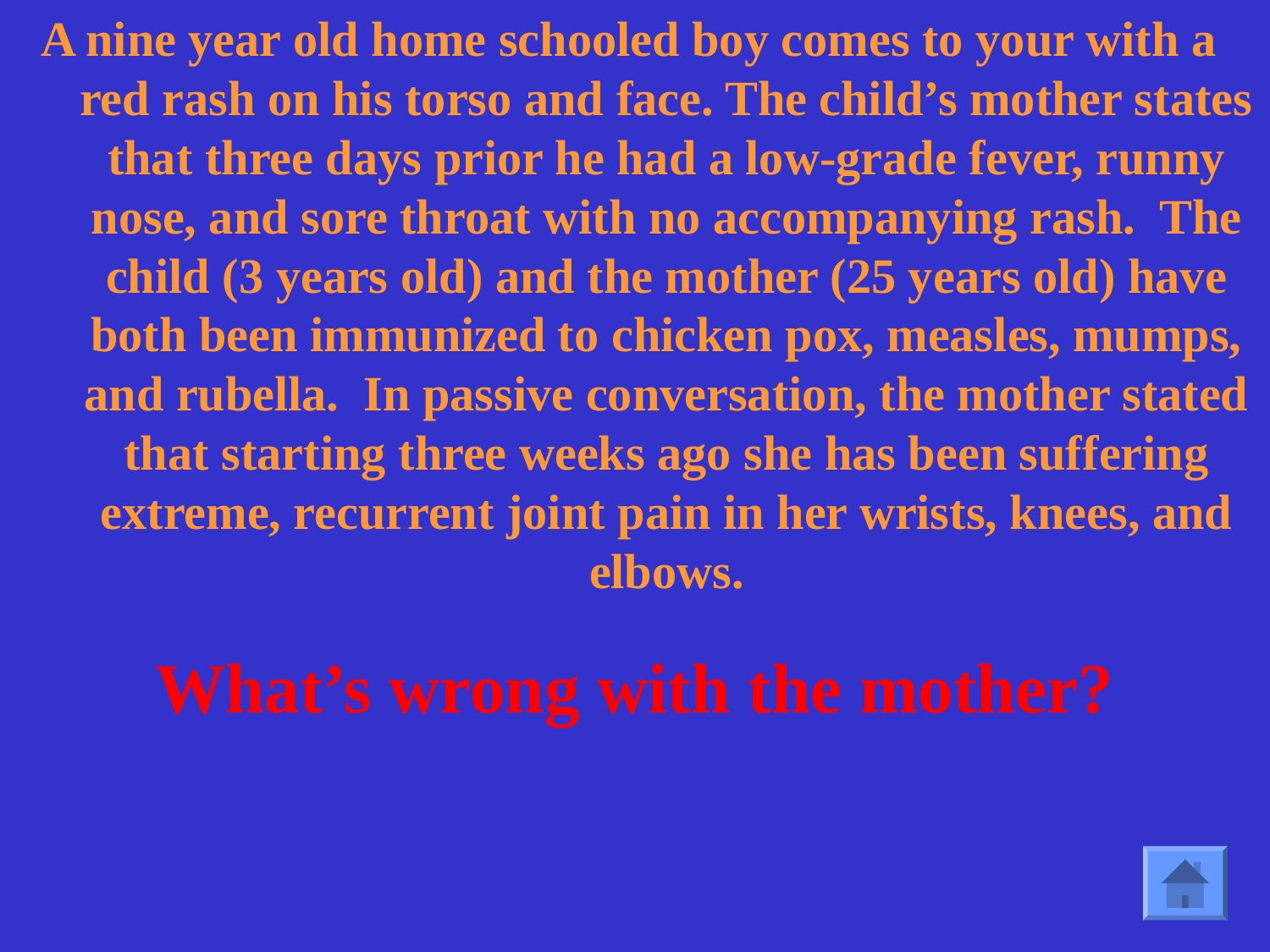

A nine year old home schooled boy comes to your with a red rash on his torso and face. The child’s mother states that three days prior he had a low-grade fever, runny nose, and sore throat with no accompanying rash. The child (3 years old) and the mother (25 years old) have both been immunized to chicken pox, measles, mumps, and rubella. In passive conversation, the mother stated that starting three weeks ago she has been suffering extreme, recurrent joint pain in her wrists, knees, and elbows.
What’s wrong with the mother?

## Slide 50
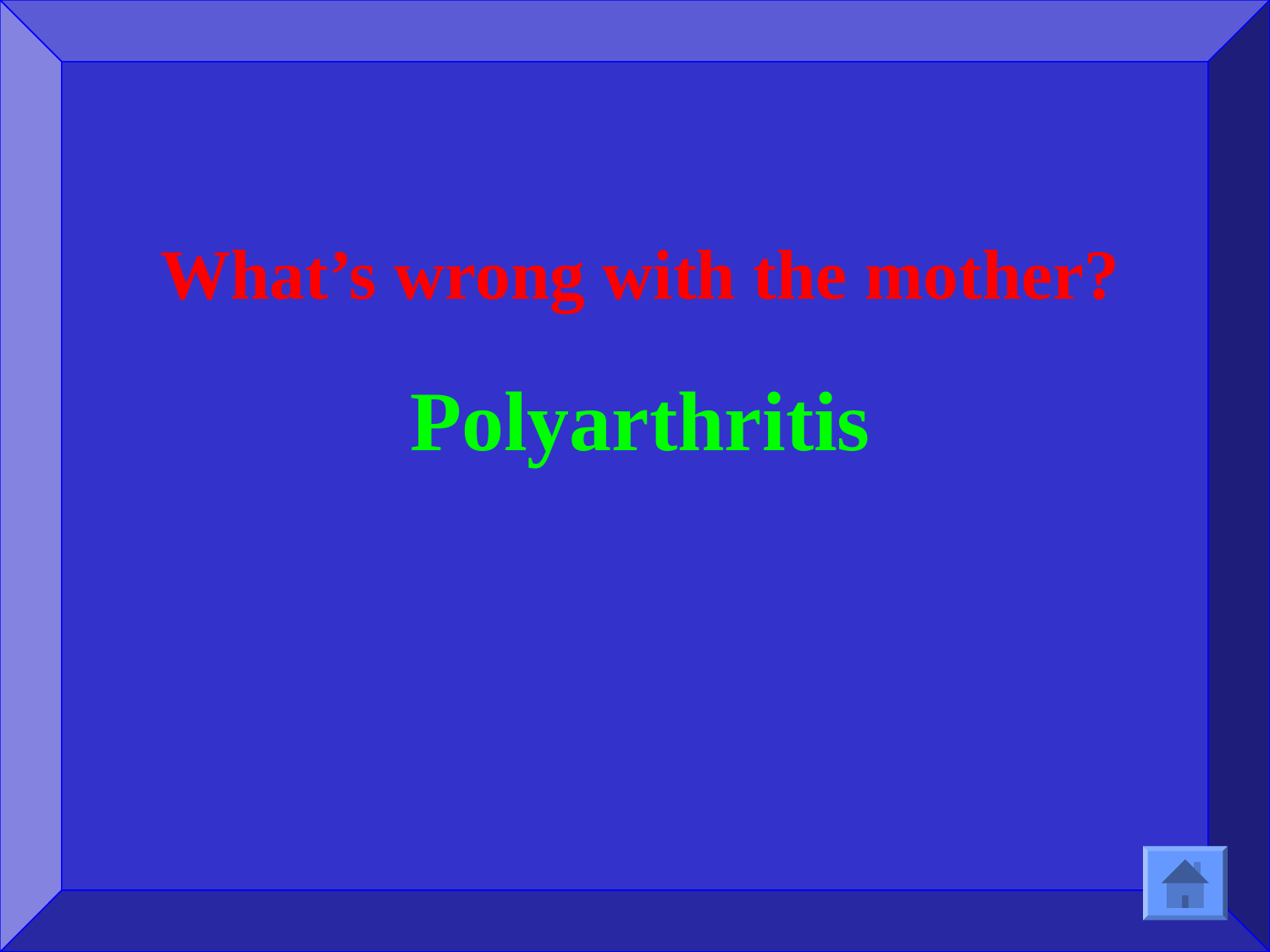

What’s wrong with the mother?
Polyarthritis

## Slide 51
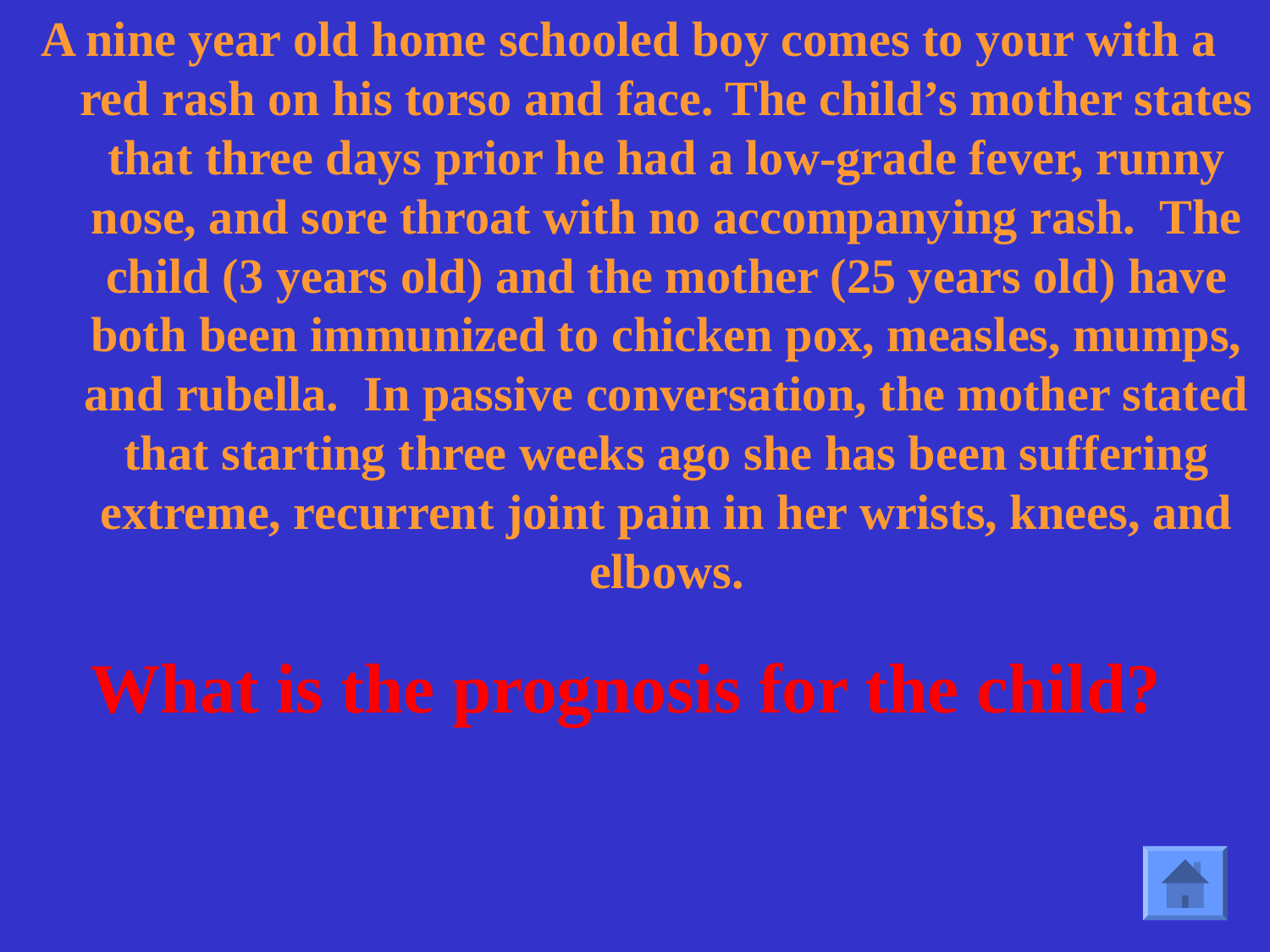

A nine year old home schooled boy comes to your with a red rash on his torso and face. The child’s mother states that three days prior he had a low-grade fever, runny nose, and sore throat with no accompanying rash. The child (3 years old) and the mother (25 years old) have both been immunized to chicken pox, measles, mumps, and rubella. In passive conversation, the mother stated that starting three weeks ago she has been suffering extreme, recurrent joint pain in her wrists, knees, and elbows.
What is the prognosis for the child?

## Slide 52
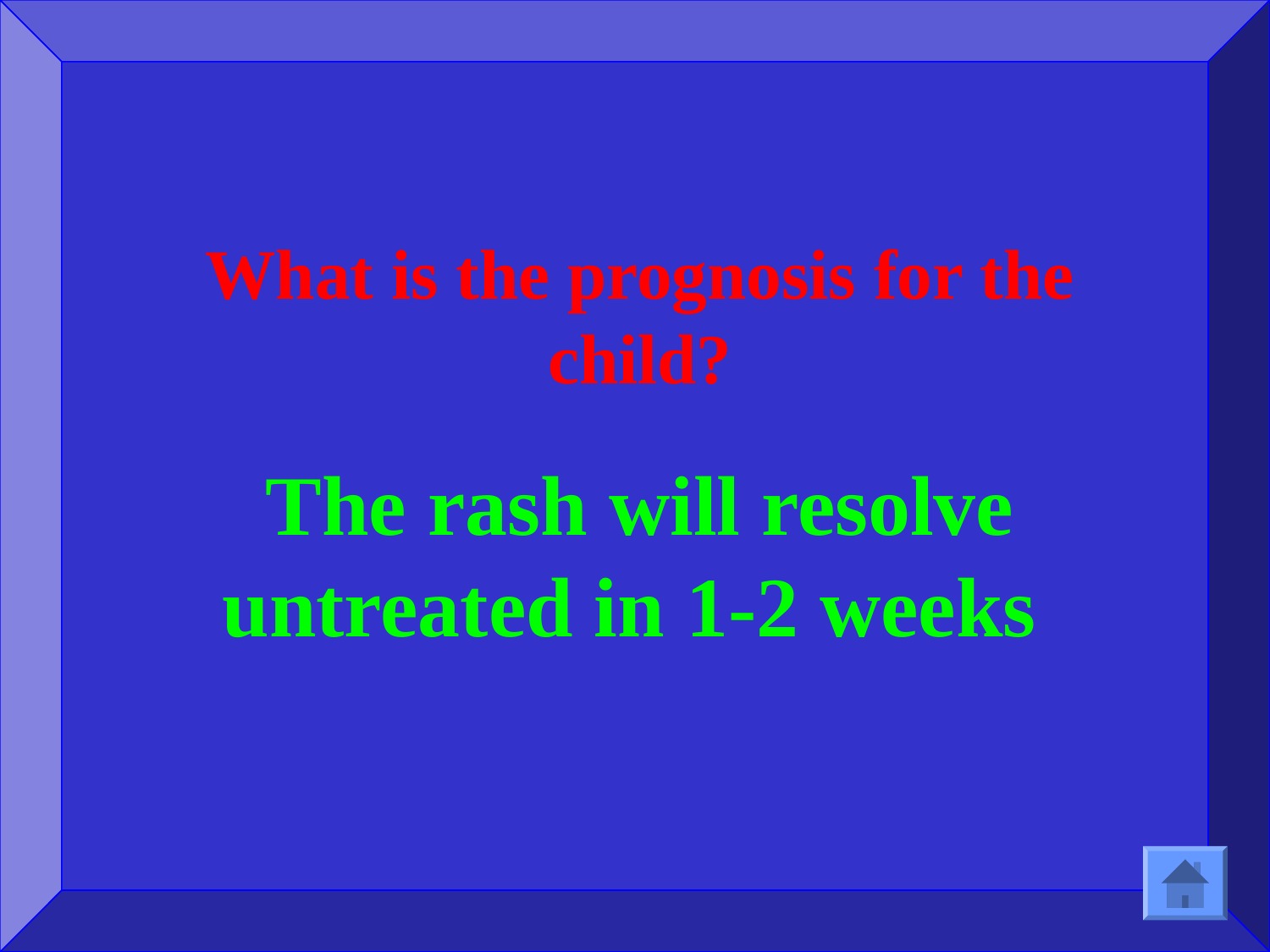

What is the prognosis for the child?
The rash will resolve untreated in 1-2 weeks

## Slide 53
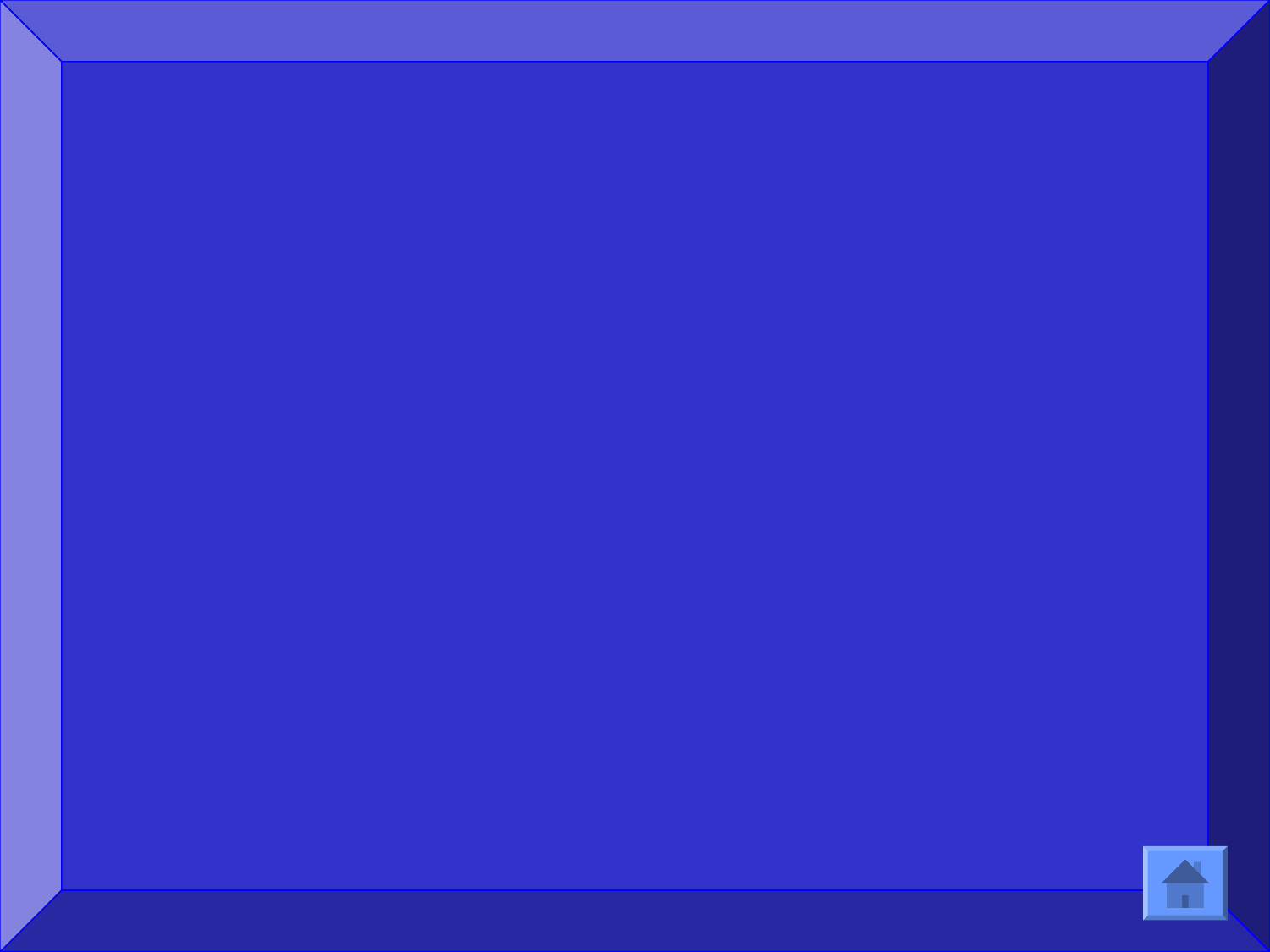

## Slide 54
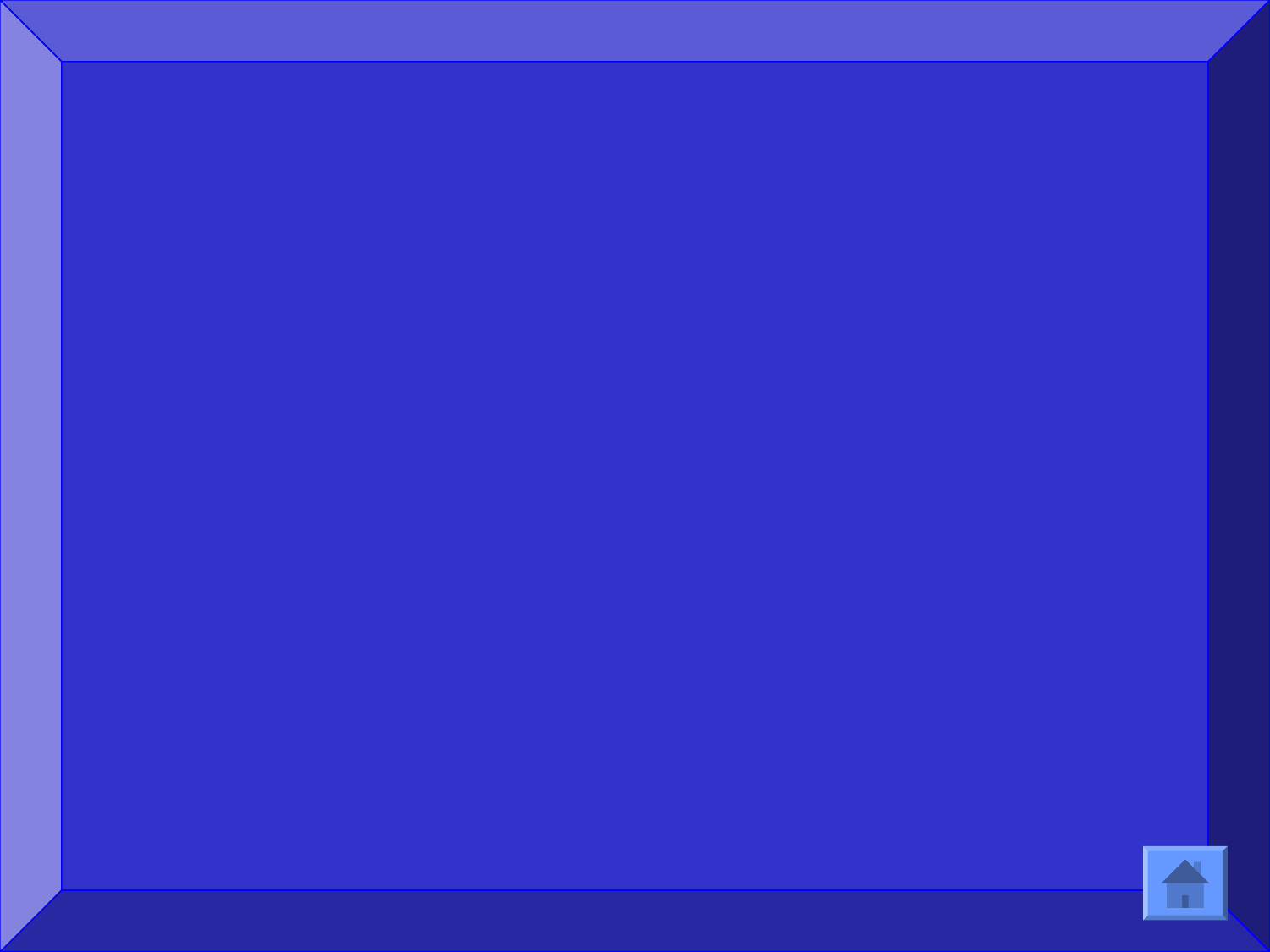

## Slide 55
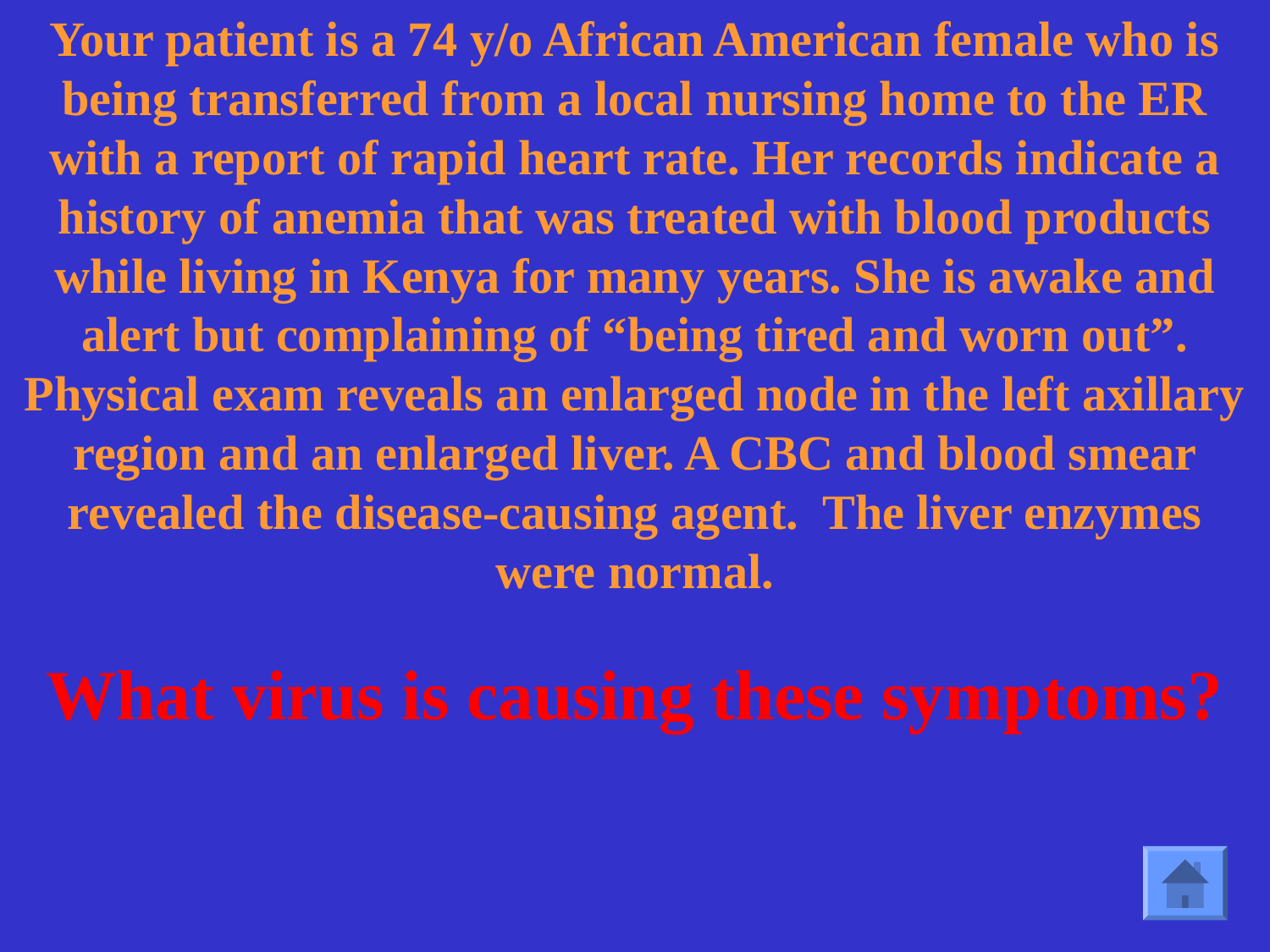

Your patient is a 74 y/o African American female who is being transferred from a local nursing home to the ER with a report of rapid heart rate. Her records indicate a history of anemia that was treated with blood products while living in Kenya for many years. She is awake and alert but complaining of “being tired and worn out”. Physical exam reveals an enlarged node in the left axillary region and an enlarged liver. A CBC and blood smear revealed the disease-causing agent. The liver enzymes were normal.
What virus is causing these symptoms?

## Slide 56
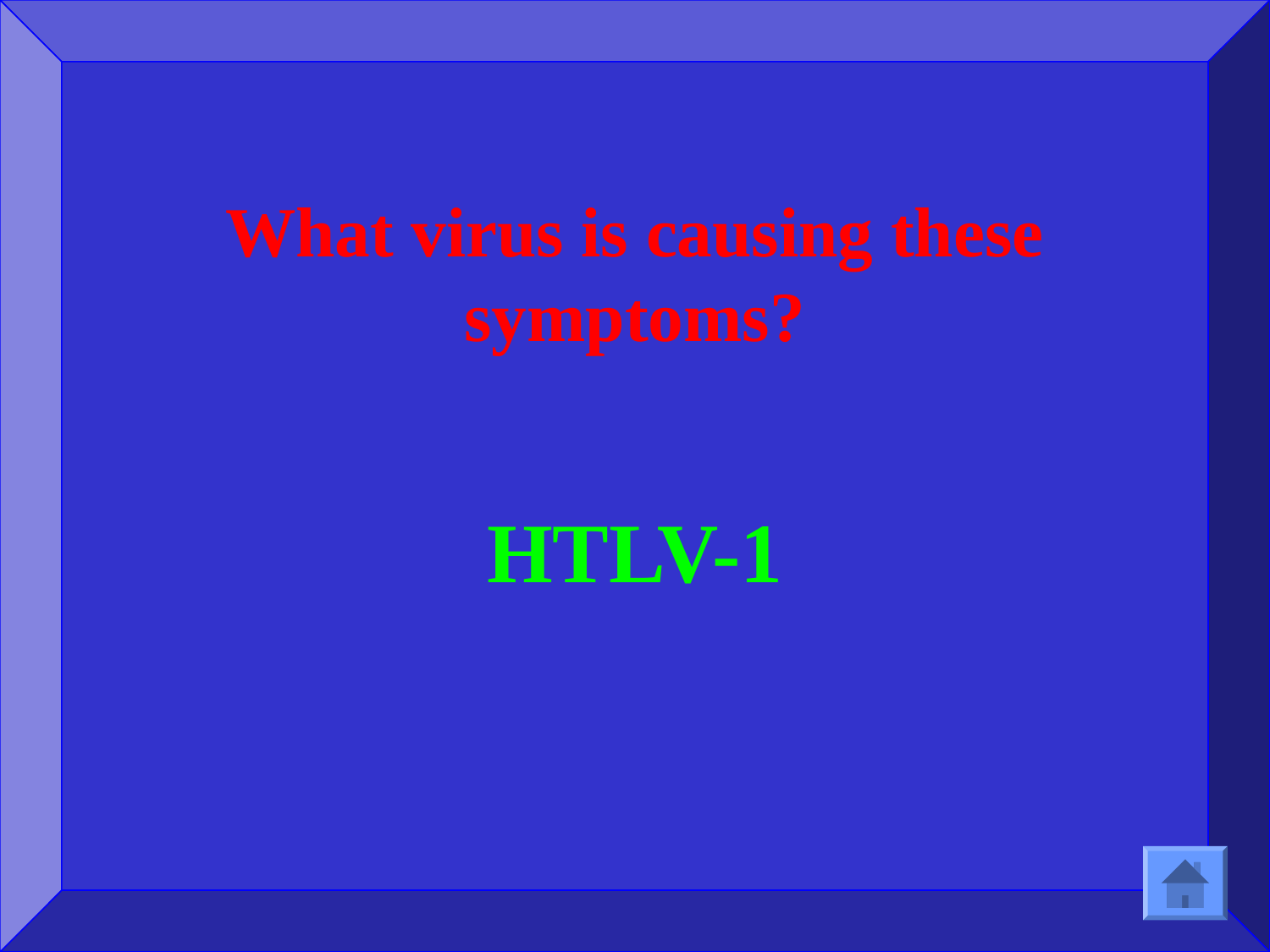

What virus is causing these symptoms?
HTLV-1

## Slide 57
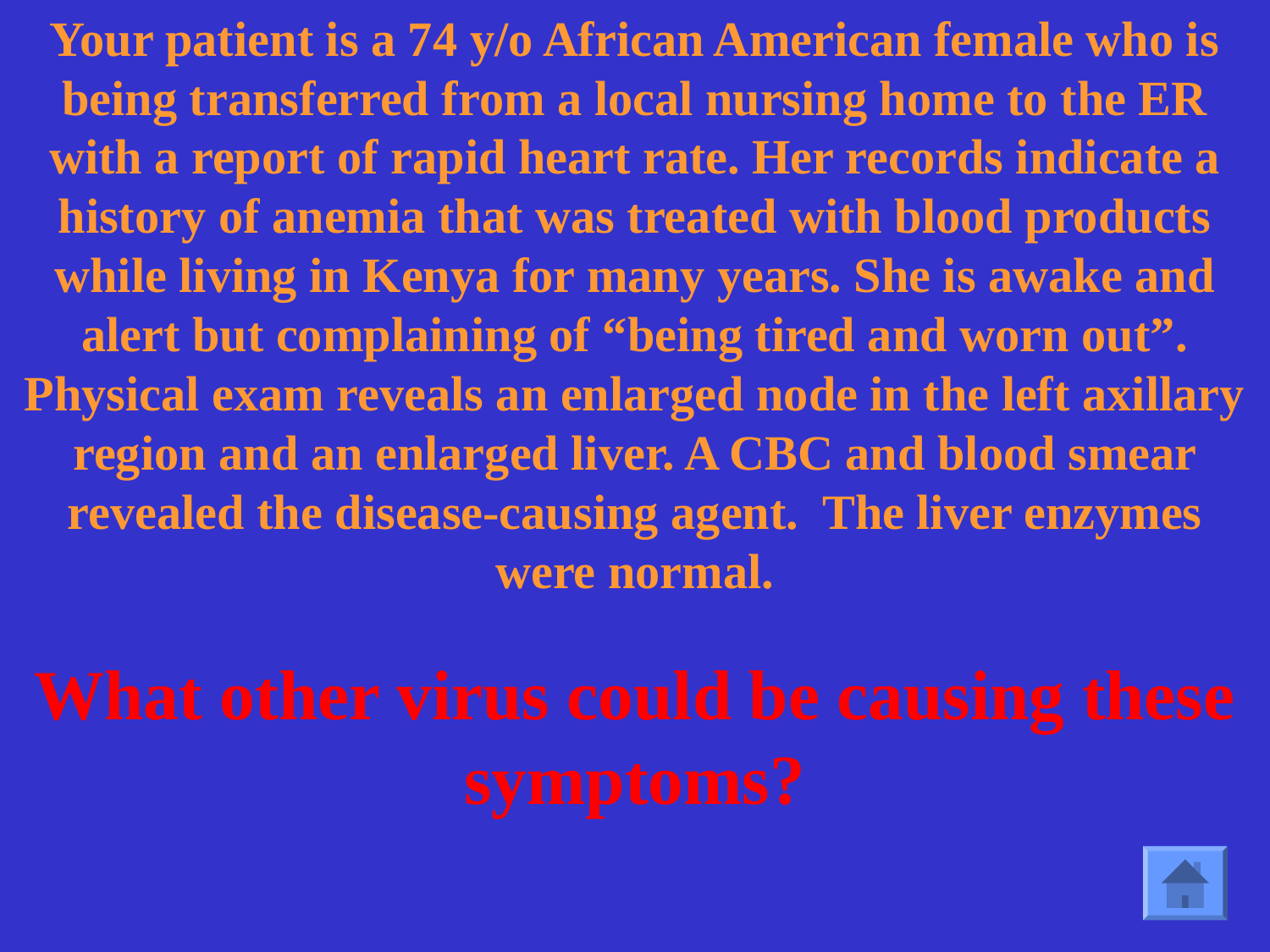

Your patient is a 74 y/o African American female who is being transferred from a local nursing home to the ER with a report of rapid heart rate. Her records indicate a history of anemia that was treated with blood products while living in Kenya for many years. She is awake and alert but complaining of “being tired and worn out”. Physical exam reveals an enlarged node in the left axillary region and an enlarged liver. A CBC and blood smear revealed the disease-causing agent. The liver enzymes were normal.
What other virus could be causing these symptoms?

## Slide 58
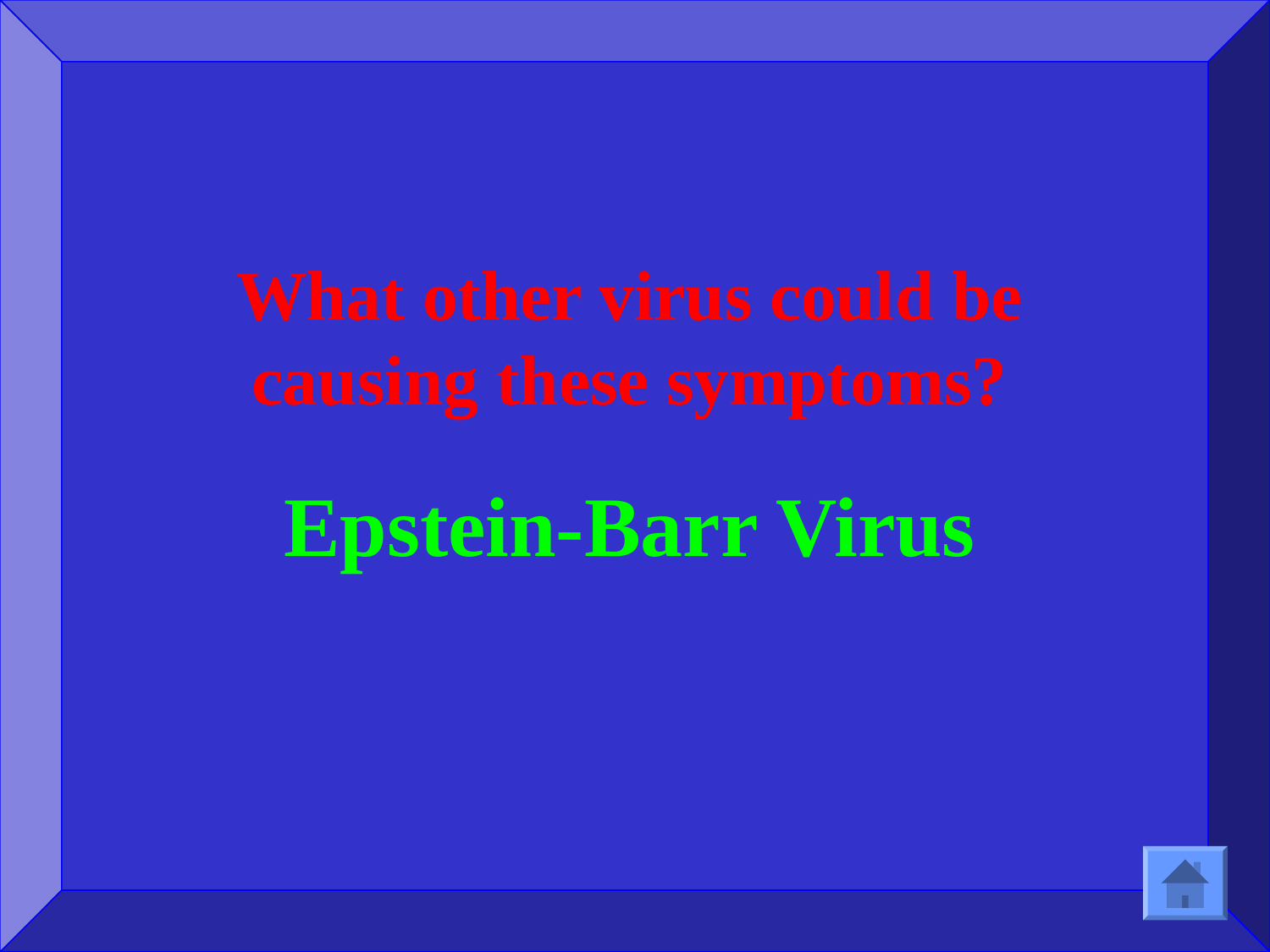

What other virus could be causing these symptoms?
Epstein-Barr Virus

## Slide 59
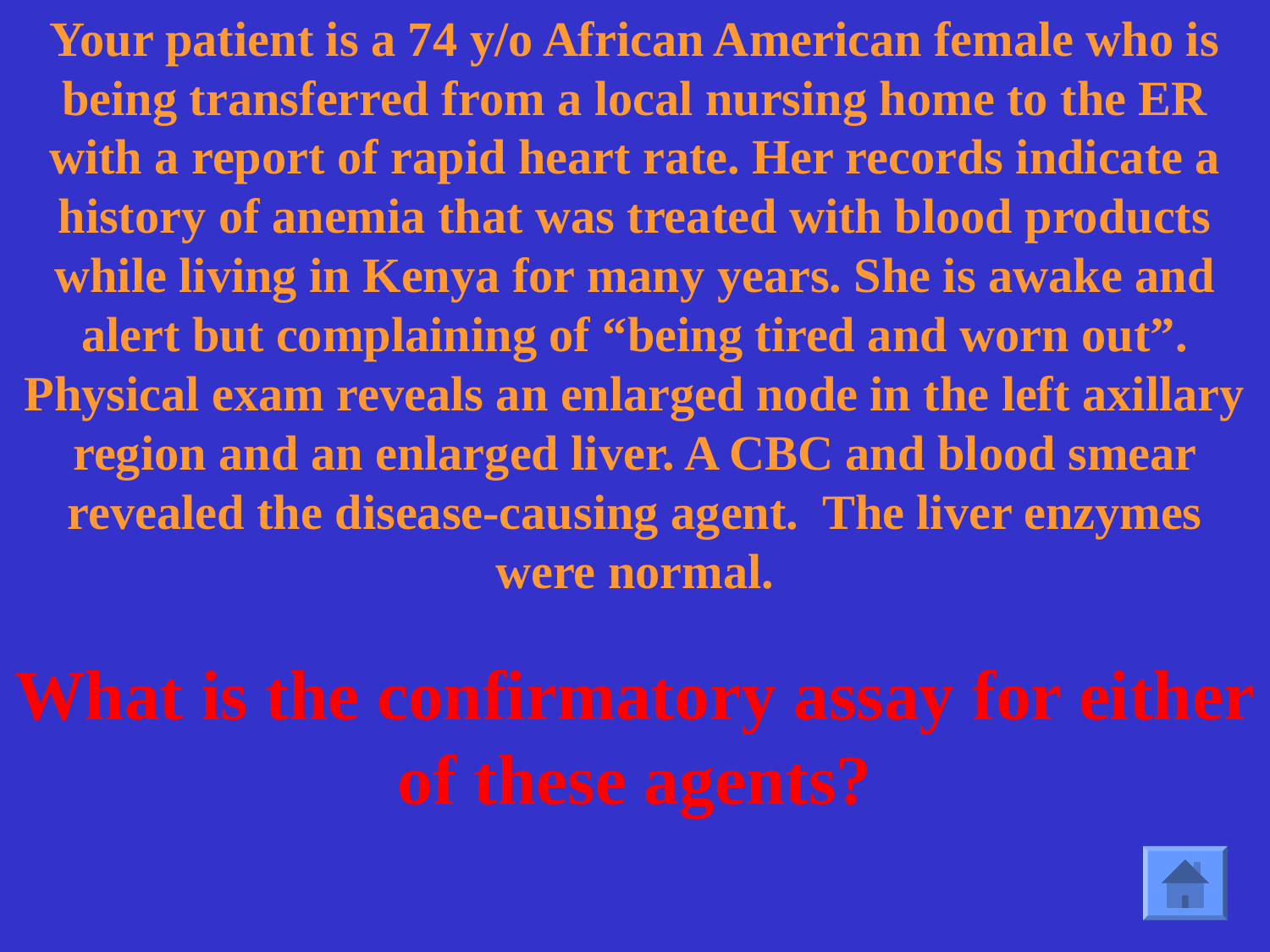

Your patient is a 74 y/o African American female who is being transferred from a local nursing home to the ER with a report of rapid heart rate. Her records indicate a history of anemia that was treated with blood products while living in Kenya for many years. She is awake and alert but complaining of “being tired and worn out”. Physical exam reveals an enlarged node in the left axillary region and an enlarged liver. A CBC and blood smear revealed the disease-causing agent. The liver enzymes were normal.
What is the confirmatory assay for either of these agents?

## Slide 60
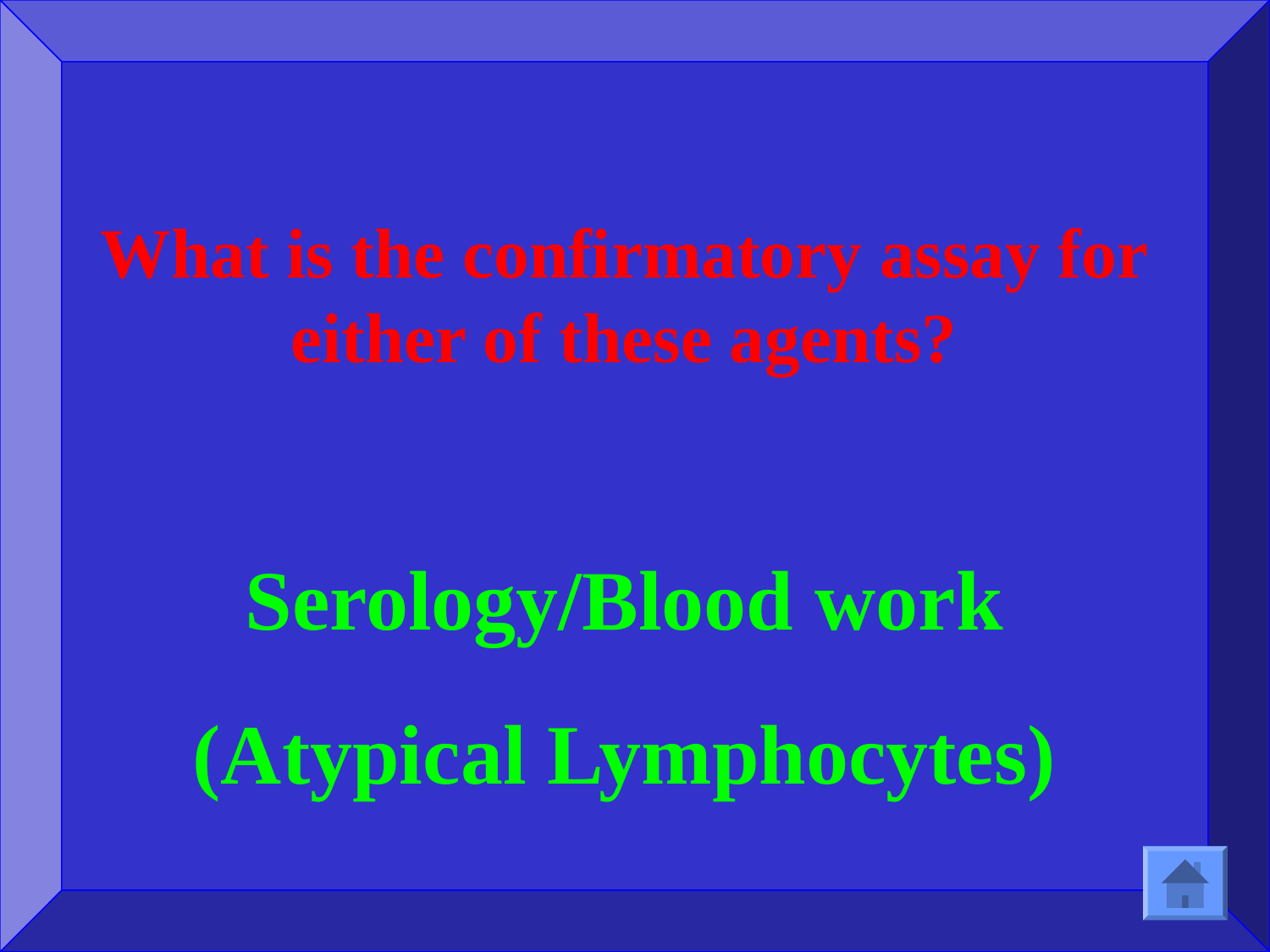

What is the confirmatory assay for either of these agents?
Serology/Blood work
(Atypical Lymphocytes)

## Slide 61
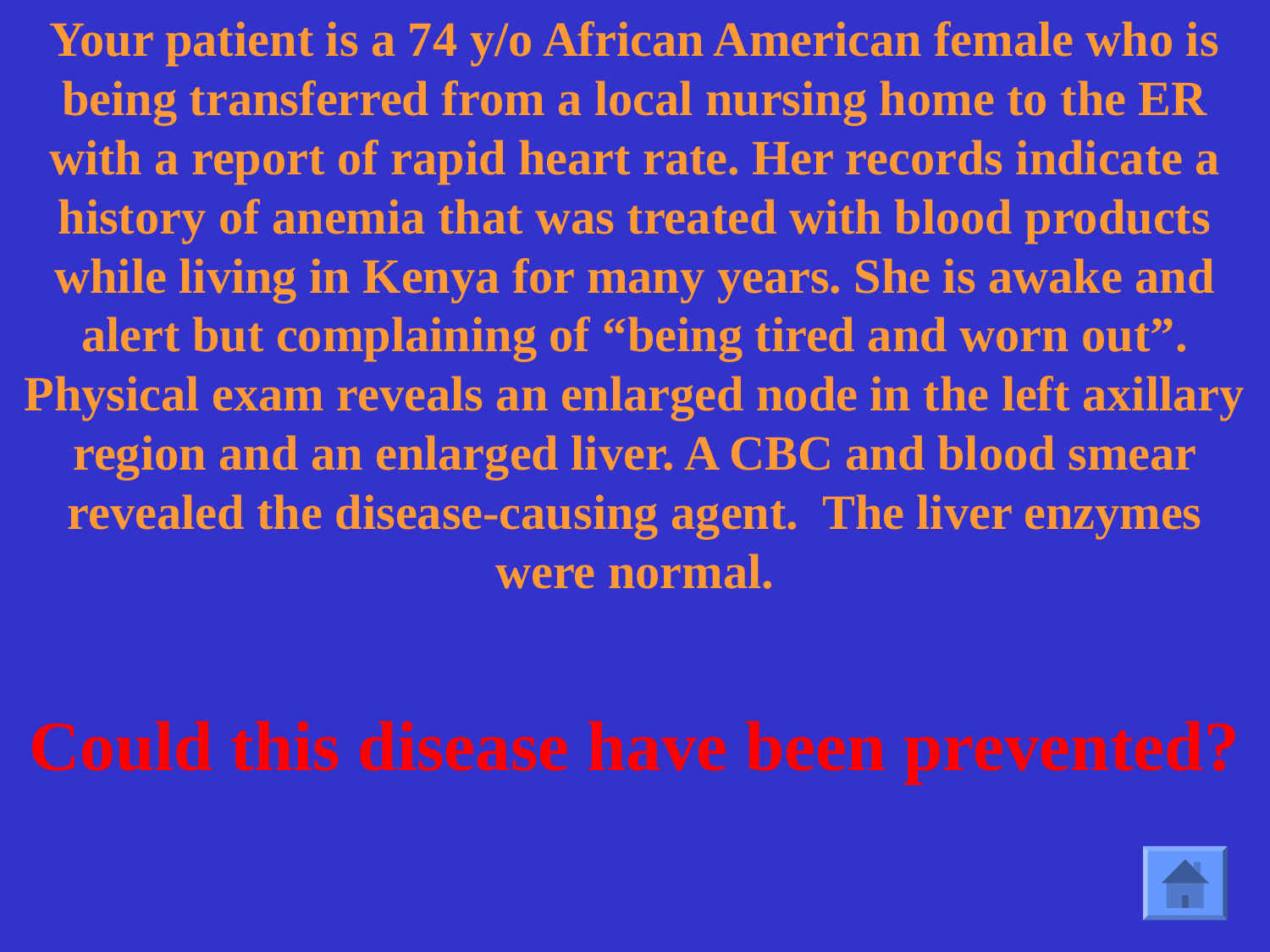

Your patient is a 74 y/o African American female who is being transferred from a local nursing home to the ER with a report of rapid heart rate. Her records indicate a history of anemia that was treated with blood products while living in Kenya for many years. She is awake and alert but complaining of “being tired and worn out”. Physical exam reveals an enlarged node in the left axillary region and an enlarged liver. A CBC and blood smear revealed the disease-causing agent. The liver enzymes were normal.
Could this disease have been prevented?

## Slide 62
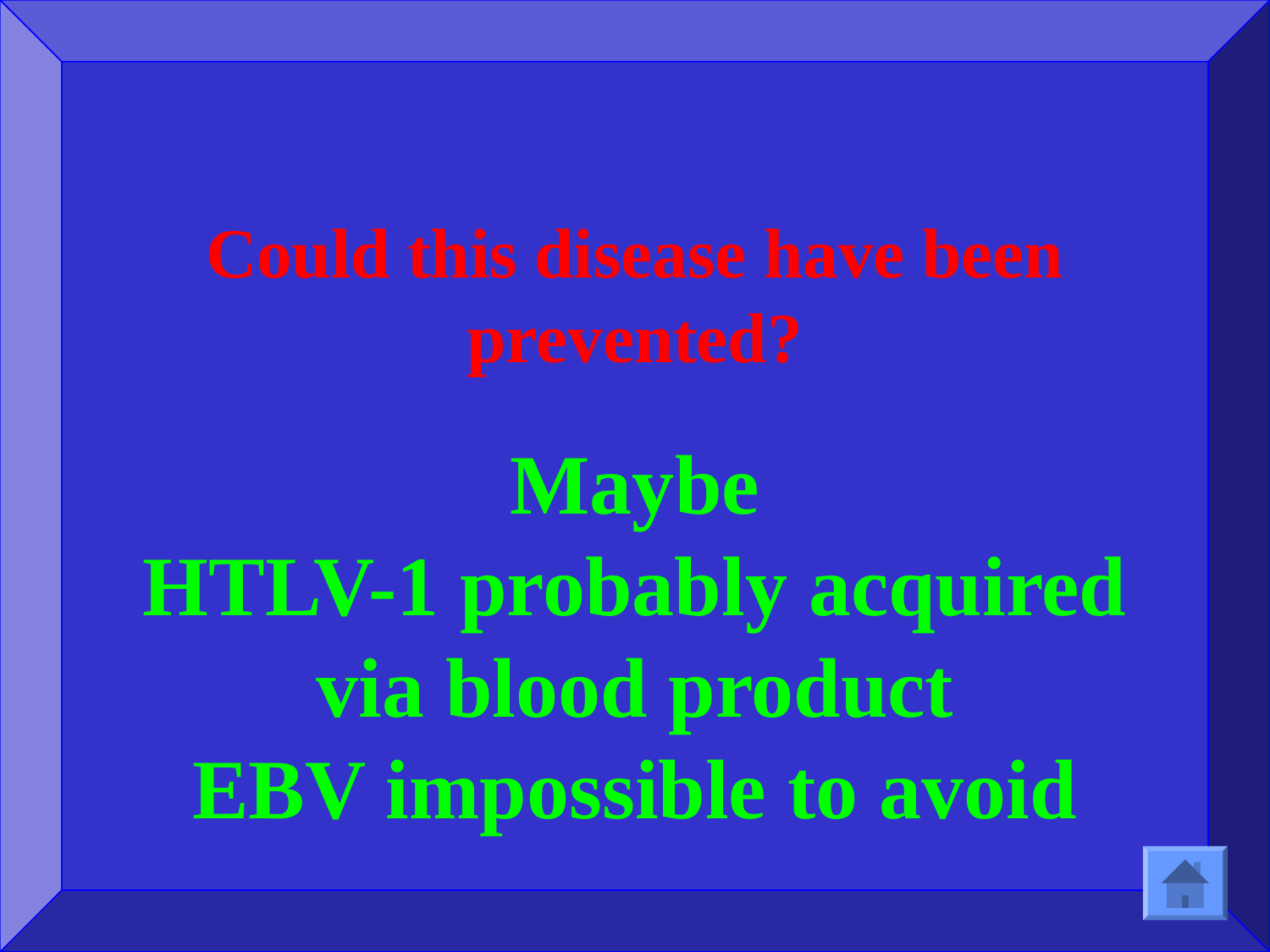

Could this disease have been prevented?
Maybe
HTLV-1 probably acquired via blood product
EBV impossible to avoid

## Slide 63
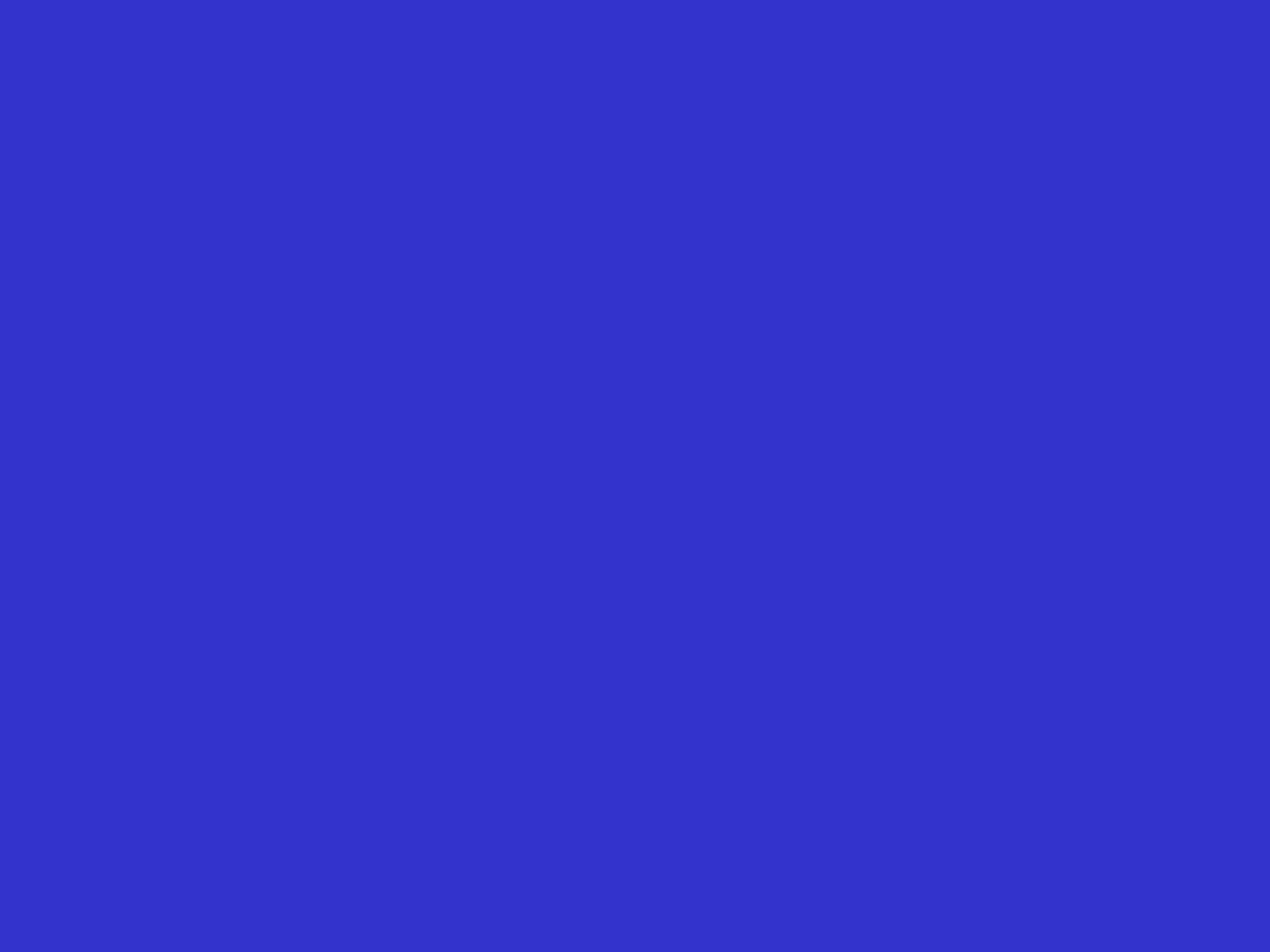

## Slide 64
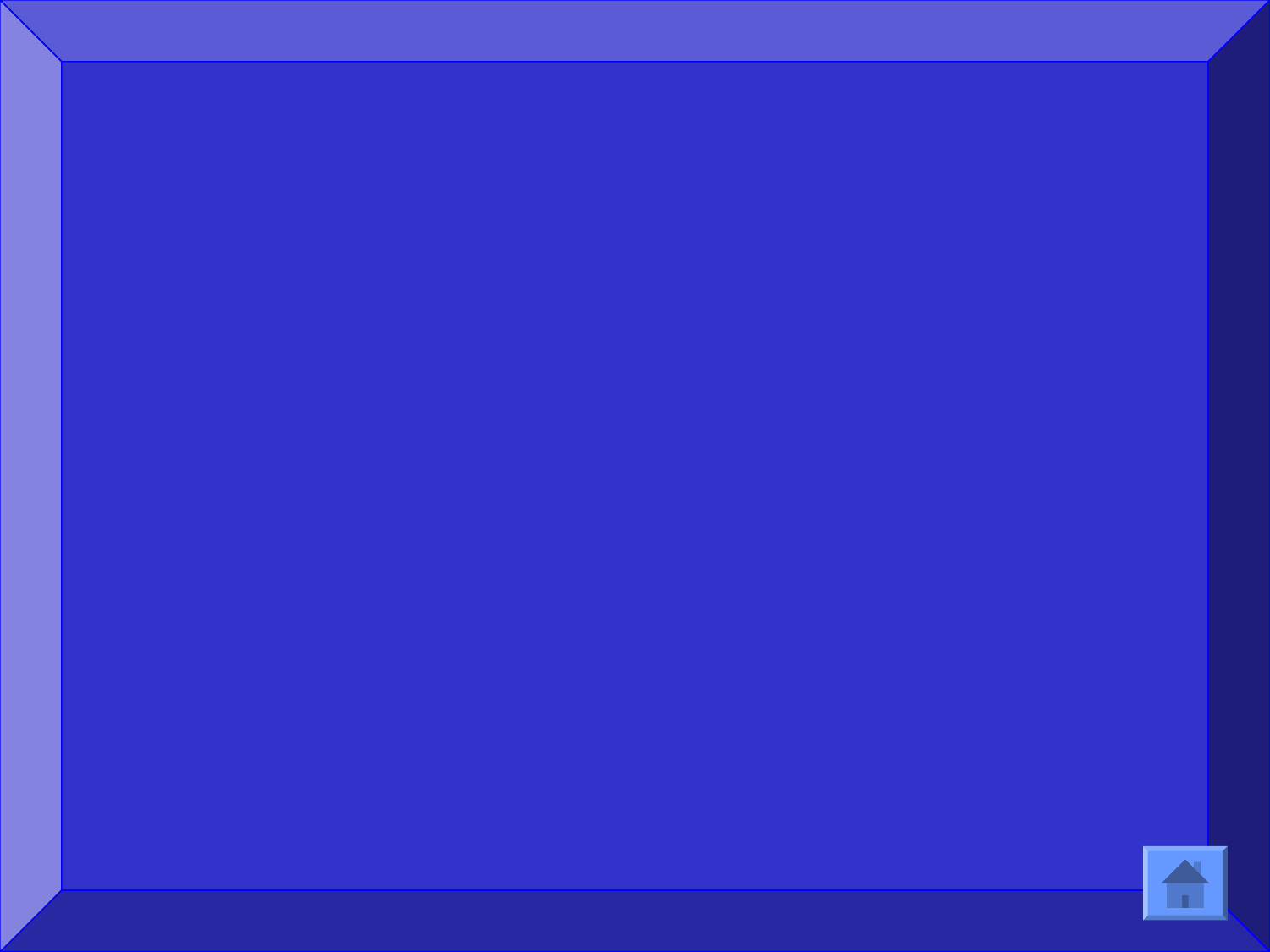

## Slide 65
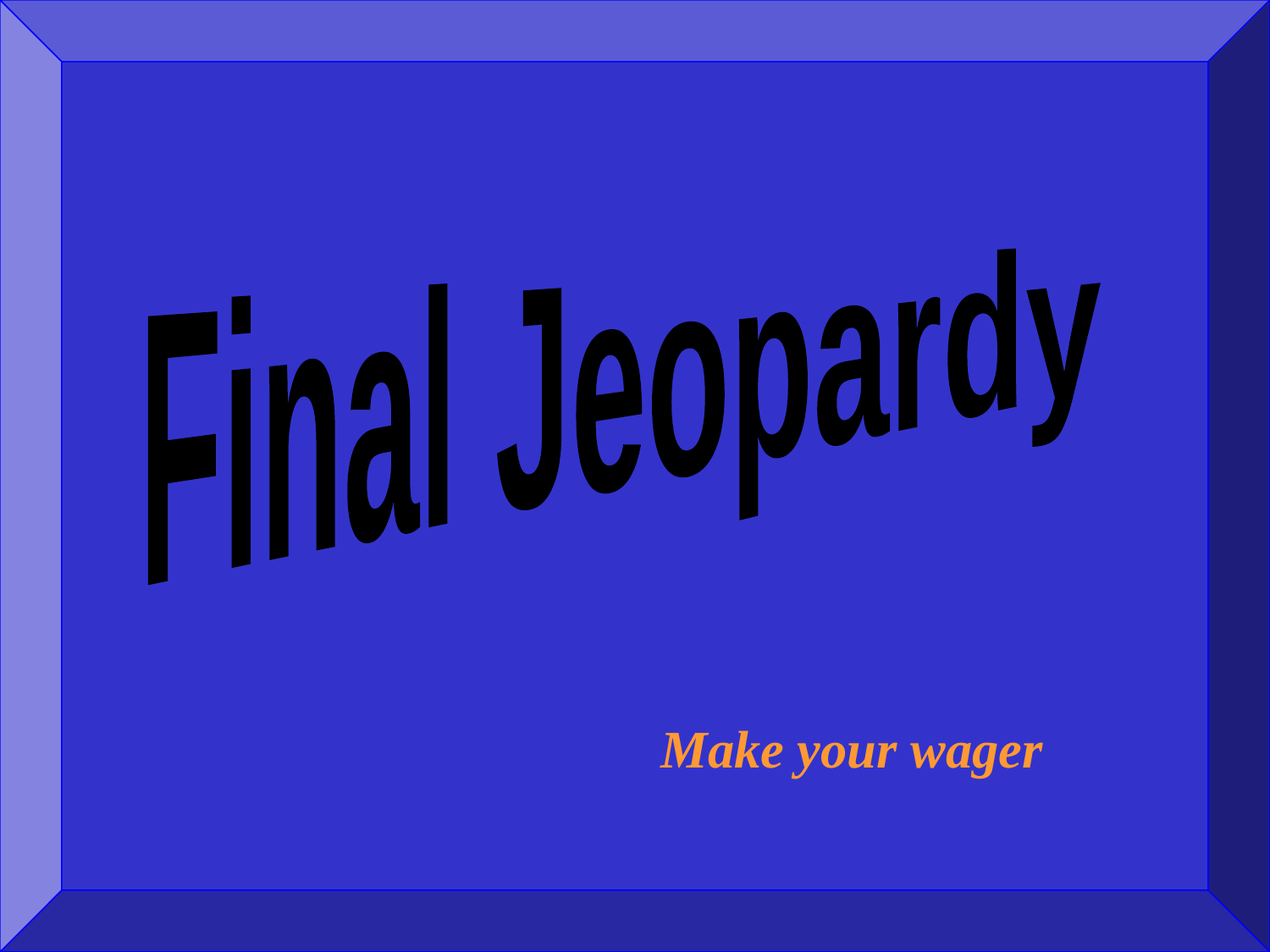

Final Jeopardy
Make your wager

## Slide 66
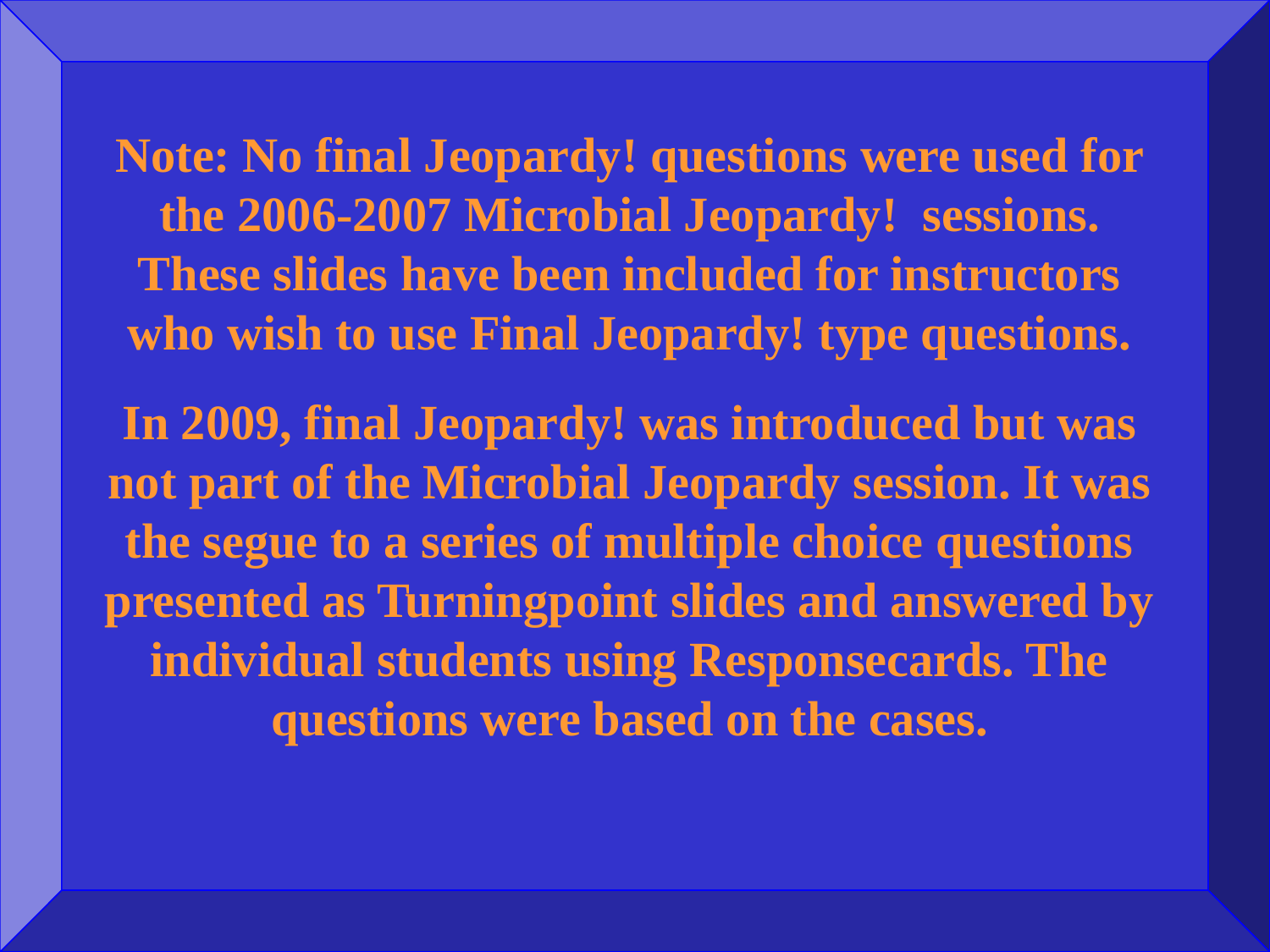

Note: No final Jeopardy! questions were used for the 2006-2007 Microbial Jeopardy! sessions. These slides have been included for instructors who wish to use Final Jeopardy! type questions.
In 2009, final Jeopardy! was introduced but was not part of the Microbial Jeopardy session. It was the segue to a series of multiple choice questions presented as Turningpoint slides and answered by individual students using Responsecards. The questions were based on the cases.

## Slide 67
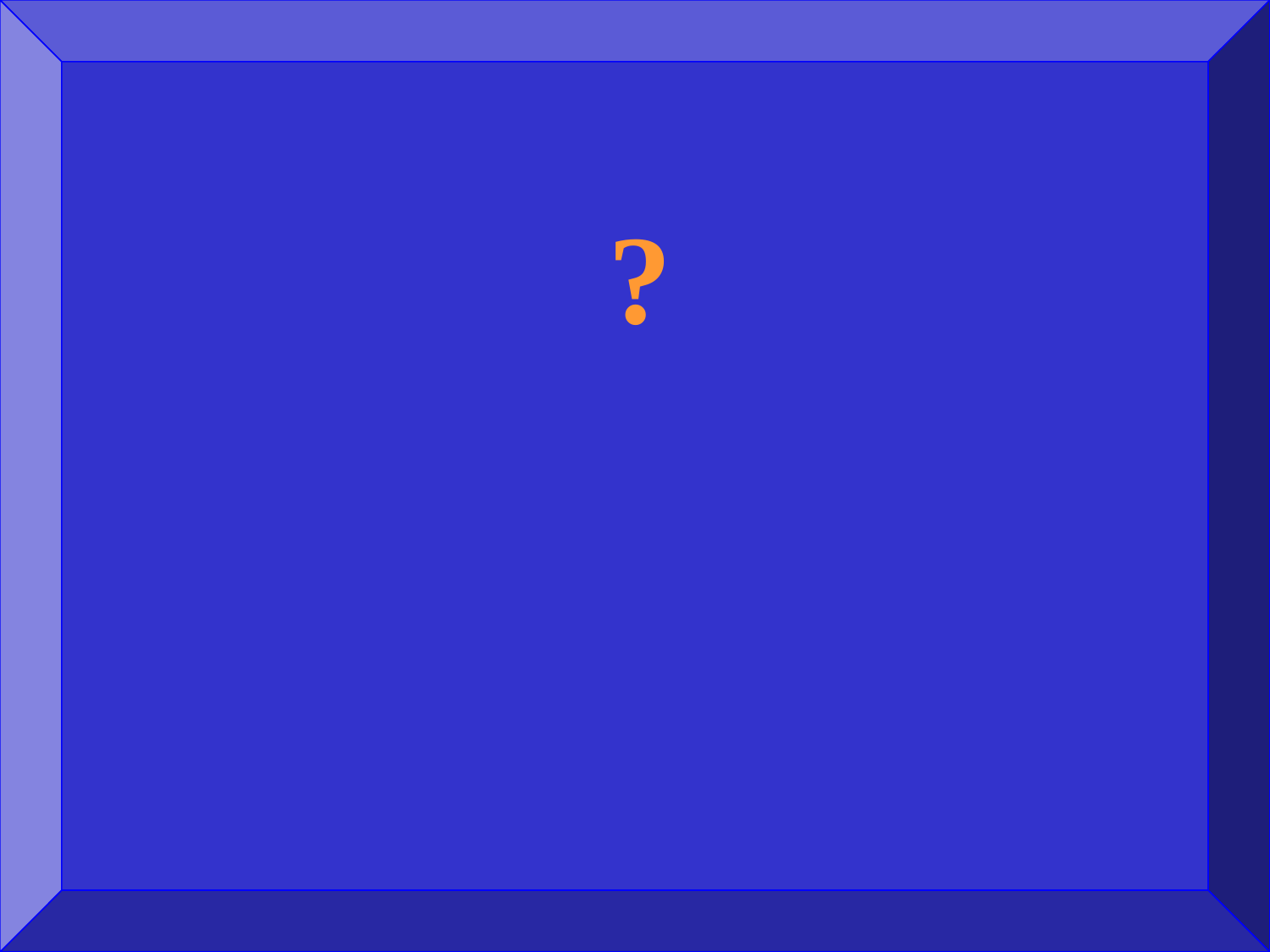

?
